# Cuproptosis-associated genes and immune microenvironment characterization in breast cancer

**DOI:** 10.1097/MD.0000000000032301

**Published:** 2022-12-16

**Authors:** Lijuan Shen, Youwu He, Chunhui Fang, Haiyan Qiu, Qing Chen, Fang Huang, Zhengyuan Wu

**Affiliations:** a Department of Hand Plastic Surgery, The First People’s Hospital of Linping District, Hangzhou, China; b Department of Orthopedics, The First People’s Hospital of Linping District, Hangzhou, China.

**Keywords:** breast cancer, cuproptosis, gene signature, immune microenvironment, immunotherapy, prognosis

## Abstract

Excess Cu can cause cell death as a cofactor for essential enzymes. The relationship between cuproptosis-associated genes (CAGs) and breast cancer (BR) is not completely investigated. Here, the transcriptome expression and mutation profile data of BR samples from the Cancer Genome Atlas database were retrieved to identify CAGs. Patients with BR were clustered using consensus clustering. A least absolute shrinkage and selection operator analysis was then performed to construct a CAGs risk signature. As a result, all 13 cuproptosis regulators were significantly differentially expressed between BR and normal samples; among them, 9 cuproptosis genes were correlated with prognoses. Patients with BR were separated into 2 clusters that were associated with patient survival, clinical phenotypes, and immune infiltration, Based on the components of cuproptosis. Subsequently, genes differentially expressed between clusters were obtained, and 11 CAGs were ultimately incorporated into the risk signature. Functional analyses revealed that the risk signature correlated with patient outcomes, ER, PR, HER2 expression, and BR IHC subtypes. Additionally, immune microenvironment analyses showed that CAGs-high-risk patients exhibited lower immune cell infiltration and immune functions. Furthermore, high-risk BR patients had higher TMB, lower immune checkpoint expression, higher m6A gene expression, and higher tumor stemness. Finally, the immunophenoscore analysis revealed that the risk signature could potentially predict the immune response in BR and help guide the application of various immunotherapeutic drugs. Overall, the newly constructed CAGs risk signature presented a predictive value for the prognosis and tumor microenvironment of BR patients and can be further used in the guidance of immunotherapy for BR.

## 1. Introduction

Breast cancer (BR) is considered as the most frequently diagonalized cancer and the major cause of death in women worldwide.^[1]^ Recently, researchers have attempted to identify novel biomarkers that can be used for prognostic prediction and for the personalized therapy of patients, and as a result, the 5-year survivorship of BR patients has improved to 90%.^[2]^ However, owing to its heterogeneity, different types of BR may present with completely different prognosis.^[3,4]^ Therefore, the identification of new biomarkers for prognosis and therapeutic sensitivity predictions of BR patients is urgently needed.

As a novel uncharacterized cell death mechanism, cuproptosis can result in the cytotoxicity of human cells via protein lipoylation, a mechanism distinct from all other known cell death regulation mechanisms, including ferroptosis, apoptosis, necroptosis, and pyroptosis. Lipoylation is necessary for enzymatic function in the tricarboxylic acid (TCA) cycle; thus, most lipoylated proteins are concentrated around this process.^[5,6]^ During respiration, the levels of lipoylated TCA enzymes, particularly those associated with the pyruvate dehydrogenase (PDH) complex, are increased in TCA-cycle activated cells. These moieties can act as copper binders, resulting in the aggregation of lipoylated proteins, a loss of Fe-S cluster-containing proteins, and HSP70 induction, ultimately leading to acute proteotoxic stress.^[7]^ Targeting this specific mechanism, copper ionophore elesclomol displayed effective anti-tumor activity in cancer patients, such as melanoma, and this effect was especially associated with lower plasma lactate dehydrogenase levels, which reflect a higher mitochondrial metabolism and increased TCA enzyme lipoylation levels.^[8]^ Therefore, the cuproptosis approach has potential applications in cancer treatment. However, a little exploitation of cuproptosis has been successfully undertaken in cancer cells, and the role of cuproptosis in cancer immunity is still unknown.

Herein, to investigate the role of cuproptosis genes in BR prognosis and patient clusters, various bioinformatic analyses were conducted. We focused on the potential role of cuproptosis-associated genes (CAGs) in BR and screened several hub prognostic CAGs to construct a risk signature. The prognostic value and correlation with the immune microenvironment, tumor mutational burden (TMB), m6A-related genes, tumor stemness, and immune therapy response of this signature were further explored in patients with BR. Currently, the risk signature generated according to the expression of CAGs in the BR has not yet been performed. Thus, to the best of our knowledge, the present study is the first to construct a CAGs risk signature for the prediction of BR prognosis.

## 2. Materials and Methods

### 2.1. Raw data acquisition

Transcriptomic data, clinical information, and somatic mutation data for BR tissues were collected from The Cancer Genome Atlas (TCGA) database (https://portal.gdc.cancer.gov). Log2-transformations were performed using the “sva” package in R to remove batch effects.^[9,10]^ In accordance with previously published articles, 13 protein domains for specific cuproptosis genes (FDX1, LIPT1, LIAS, DLD, DBT, GCSH, DLST, DLAT, PDHA1, PDHB, SLC31A1, ATP7A, and ATP7B) were collected for further analysis.

The tumor mutation burden (TMB) of BR patients was calculated using Varscan software,^[11]^ and the mutational status of the cuproptosis genes in TCGA-BR patients was explored. Then, the R package “limma” was applied to identify the differentially expressed genes which associated with cuproptosis in BR and normal samples. Additionally, the R package “survival” was further conducted to explore the association between cuproptosis genes and BR overall survival.

### 2.2. Cuproptosis genes clustering analysis

To clarify the biological properties of the 13 cuproptosis genes in BR patients’ classification, a consensus clustering analysis using the “Consensus ClusterPlus” R package was performed to divide BR patients into different clusters with 80% resampling rate, repeated 1000 times.^[12]^ Thereafter, we performed t-SNE and PCA analyses using R packages “Rtsne” and “ggplot2” respectively to explore the distribution of curoptosis gene clusters. The R package “survival” was further employed to compare the overall survival between BR patient clusters. To further quantify the relative abundance of the 23 human immune cell subtypes in different cuproptosis gene clusters, a single-sample gene set enrichment analysis was performed to obtain the score of immune signatures in each BR sample. Moreover, the “GSVA” package in R was utilized to assess the potential role of cuproptosis gene clusters among different biological processes and pathways.

### 2.3. Differentially expressed genes (DEGs) identification

After constructing cuproptosis gene clusters, DEGs among BR clusters were further acquired by the “limma” package in R with |log2 fold change| > 1 and a false discovery rate (FDR) of < 0.001. Gene ontology (GO) and Kyoto Encyclopedia of Genes and Genomes (KEGG) enrichment analyses are 2 widely applied bioinformatics tools for annotating gene functions and related pathways. In this study, GO and KEGG enrichment analyses were also utilized by the “clusterProfiler” package in R to confirm the potential mechanism of DEGs. Statistical significance was defined as false discovery rate (FDR) < 0.05.

### 2.4. Prognostic DEGs identification

The R package “survival” was employed to perform the Univariate Cox regression analysis for prognostic DEGs identification among different cuproptosis gene clusters with a cutoff of *P* < .001. Similar to the cuproptosis gene clusters, BR patients were also divided into different clusters according to the identified prognostic DEGs expression. Subsequently, the correlation between prognostic DEGs clusters, patients’ overall survival, and clinical features (including age, gender, T stage, N stage, M stage, AJCC stages, PR expression, ER expression, HER2 expression, and BR IHC subtypes) were tested and visualized by the “survival” and “pheatmap” packages in R, respectively. Additionally, the connection between cuproptosis genes and patient clusters was also explored.

### 2.5. Hub CAGs identification and risk signature establishment

After assessing the prognostic DEGs in patients with BR, these genes were further integrated into the least absolute shrinkage and selection operator analysis to identify the hub CAGs in BR and to generate the CAGs risk signature. Further, BR patients in the TCGA database were randomly separated into a training set and a testing set for internal validation according to a ratio of 1:1 via the “caret” package in R. The median risk score was employed as the threshold to divided the patients with BR into 2 groups with high- and low-risk scores, according to the expression of identified CAGs, BR patients were categorized into low- and high-risk groups using the median risk score as a threshold. The risk score was calculated as follows:

risk score=ΣexpCAGsi*βi

where exp CAGsi is the relative expression of the hub CAGs i and β is the regression coefficient.^[13]^

### 2.6. Predictive value of the risk signature in BR

To show the relationship between clusters, risk signature, and patients’ survival status, an alluvial map was drawn using the “ggalluvial” package in R. The “survival” package was applied to compare overall survival between risk subgroups and clinical grouping information. The R package “timeROC” was applied to the risk signature for the verification of their predictive accuracy. Univariate and multivariate Cox regression analyses were performed to evaluate the relationship between the risk score and the clinical characteristics. The mutation landscape of hub CAGs in BR patients was presented by the “maftools” package in R. The correlation between the TMB and the risk score was also explored. To show the potential mechanism of hub CAGs, GO and KEGG enrichment analyses were performed as described above. Finally, a nomogram based on the levels of calculated risk scores was constructed to predict the outcomes of BR patients between 1-, 3-, and 5-years using the “rms” package in R. The calibration curves constructed by the Hosmer-Lemeshow test were applied to illustrate the consistency of the nomogram.

### 2.7. Immune microenvironment assessment

The analysis of Spearman correlation was performed to evaluate the relationship of the risk score and the ESTIMATE, stromal, and immune scores. Single-sample gene set enrichment analysis was conducted to compare the infiltration of immune cells in the 2 risk subgroups and to tevaluate the immune functions. Moreover, the CIBERSORT algorithm (https://cibersortx.stanford.edu)^[14]^ with 1000 permutations was used to calculate the correlation between the relative proportion of 22 immune cells, the risk score, and hub CAGs. Additionally, immune checkpoint molecules and m6A genes retrieved from a previous study were used to explore the connection between immune-related checkpoints, m6A genes, and risk signatures.^[15]^ Spearman correlation analysis was used to measure the relationship between the risk signature and tumor stemness.

### 2.8. Immunotherapeutic exploration

As a reliable predictor of anti-PD-1 and anticytotoxic T-lymphocyte antigen-4 (CTLA-4) antibody responses,^[16]^ the immunophenoscore (IPS) was obtained from The Cancer Immunome Atlas (https://tcia.at/home) database to predict the response of immune checkpoint blockade in BR patients. Meanwhile, the R package “prophetic” was applied to evaluate the drug sensitivity of BR samples from 2 risk subgroups by determining the half-maximal inhibitory concentration (IC50).^[17]^

## 3. Results

### 3.1. Genetic variation prognoses of cuproptosis genes in BR

**Figure 1.**
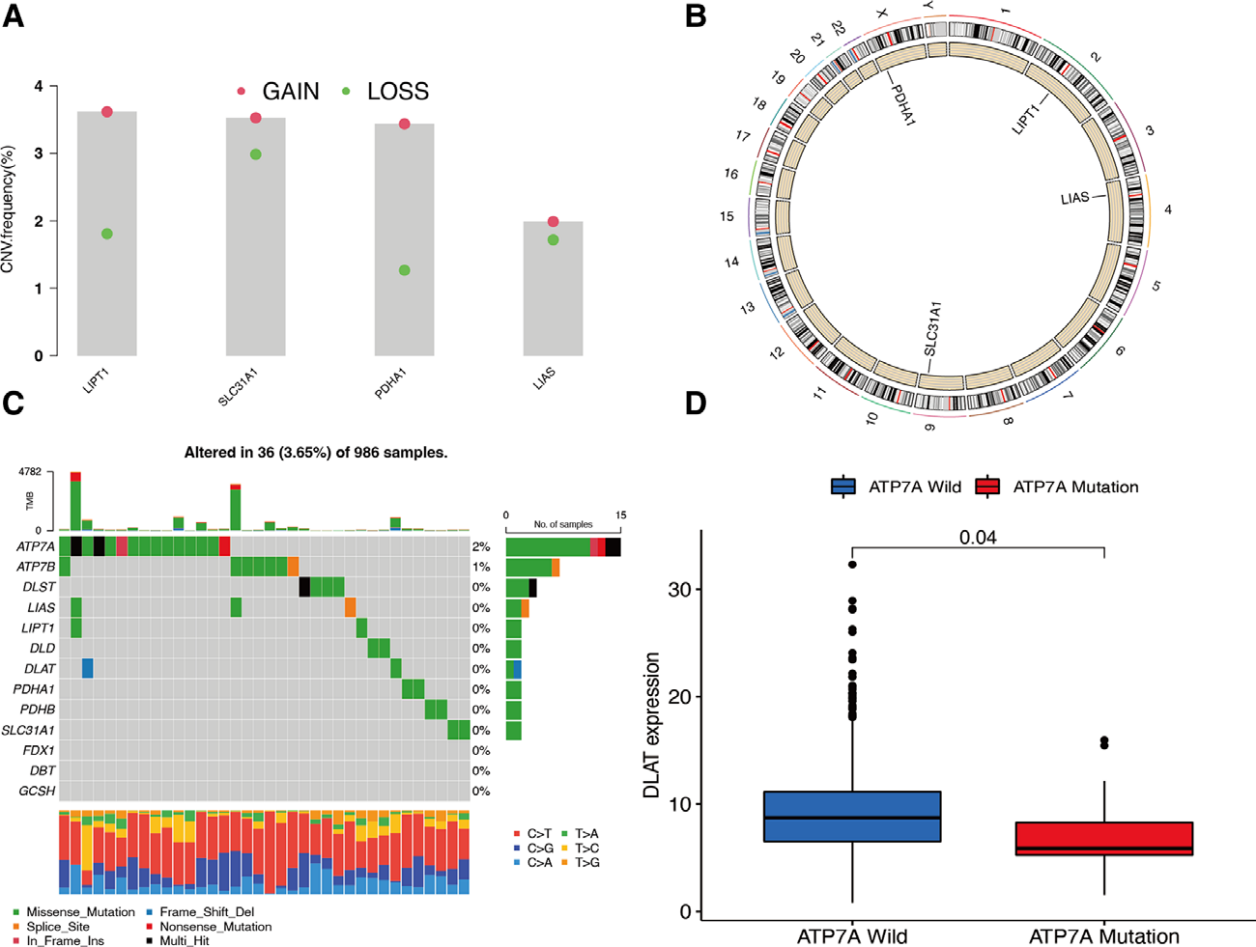
Genetic variation of cuproptosis genes in BR samples. (A) The CNV variation frequency of cuproptosis genes; red circle: amplified frequency; green circle: missing frequency. (B) The location of CNV alteration of cuproptosis genes on 23 chromosomes. (C) The mutation frequency of 13 cuproptosis genes for 986 BR samples. (D) The connection between the ATP7A mutation and cuproptosis gene DLAT expression. BR = breast cancer, CNV = copy number variation.

While exploring the changes of copy number variation (CNV) in the frequency of cuproptosis genes in BR, it was observed that CNV changes were excited in genes LIPT1, SLC31A1, PDHA1, and LIAS. Meanwhile, all the latter genes were concentrated in the gain of copy number (Fig. 1A). The CNV changes in the cuproptosis genes on the chromosome are shown in Figure 1B. Based on the mutation frequency analysis, 36 BR samples with a mutation rate of approximately 3.65% were confirmed to have cuproptosis gene mutations (Fig. 1C). Additionally, only ATP7A, ATP7B, DLST, LIAS, LIPT1, DLD, DLAT, PDHA1, PDHB, and SLC31A1 had mutations in BR patients, and ATP7A had the most frequent mutation rate (2%). While determining the connection between the ATP7A mutation and the expression of cuproptosis genes, it was discovered that DLAT was significantly lower in ATP7A mutation samples than in the ATP7A wild group (*P* < .05; Fig. 1D).

**Figure 2.**
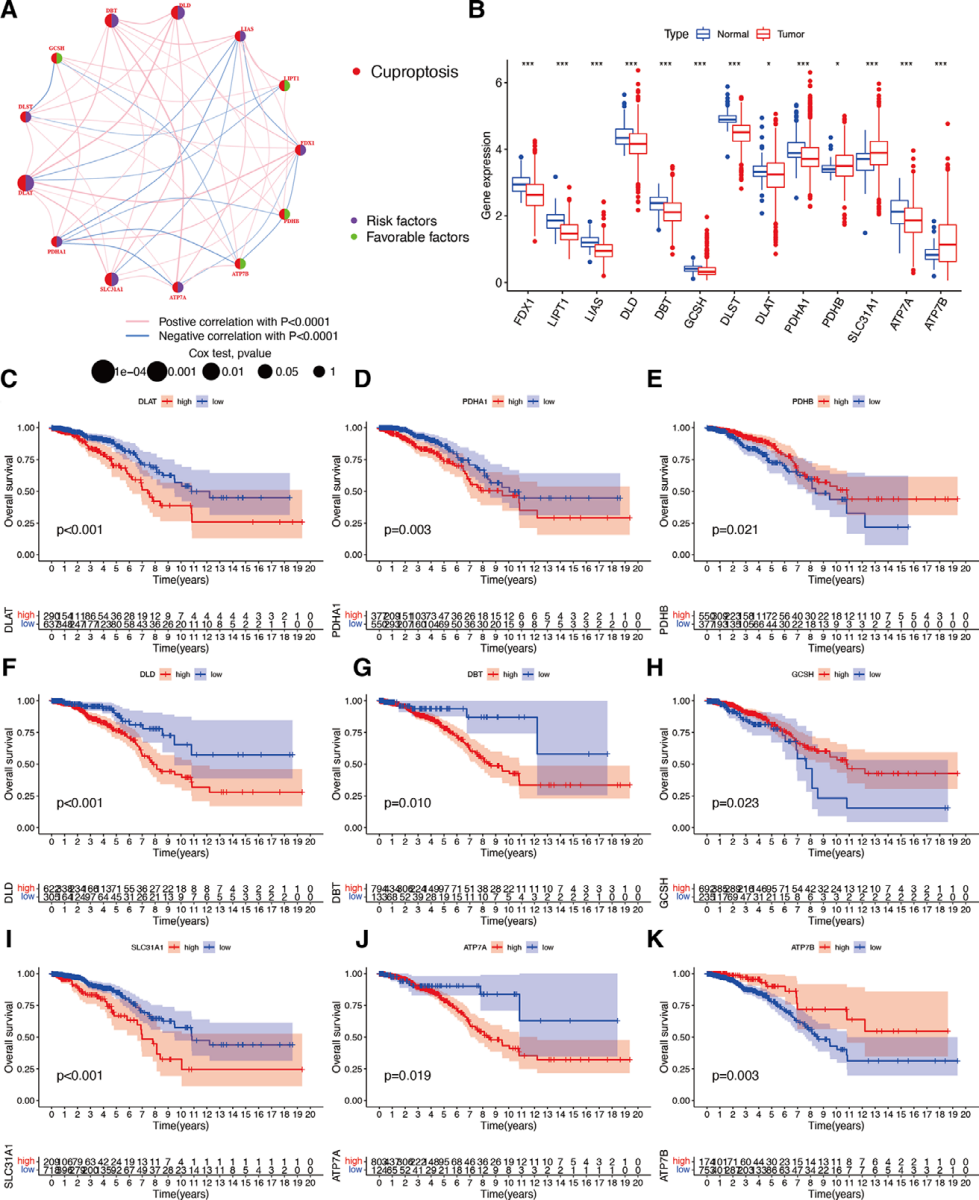
Expression and prognostic value of cuproptosis genes. (A) Correlation network of cuproptosis genes in BR patients. (B) The expression difference of 13 cuproptosis genes between normal and BR samples. The asterisk represents the statistical *P* value (*P < .05; ***P < .001). The KM curve of cuproptosis genes: DLAT (C), PDHA1 (D), PDHB (E), DLD (F), DBT (G), GCSH (H), SLC31A1 (I), ATP7A (J), and ATP7B (K) in BR. BR = breast cancer.

While exploring the associations between cuproptosis genes and overall survival of BR patients, the results indicated that GCSH, LIPT1, PDHB, and ATP7B were favorable factors in BR patients, whereas DLST, DLAT, PDHA1, SLC31A1, ATP7A, FDX1, LIAS, DLD, and DBT were risk factors (Fig. 2A). These results were confirmed by the survival analysis, which showed that PDHB (Fig. 2E), GCSH (Fig. 2H), and ATP7B (Fig. 2K) were significantly and positively correlated with BR patient prognosis, whereas DLAT (Fig. 2C), PDHA1 (Fig. 2D), DLD (Fig. 2F), DBT (Fig. 2G), SLC31A1 (Fig. 2I), and ATP7A (Fig. 2J) were negatively associated with the overall survival of BR patients. Regarding the expression level of cuproptosis genes, it was discovered that most genes were expressed at significantly lower levels in BR samples, whereas PDHB, SLC31A1, and ATP7B were highly expressed in BR patients (Fig. 2B).

### 3.2. Consensus clustering of cuproptosis genes for BR patients

**Figure 3.**
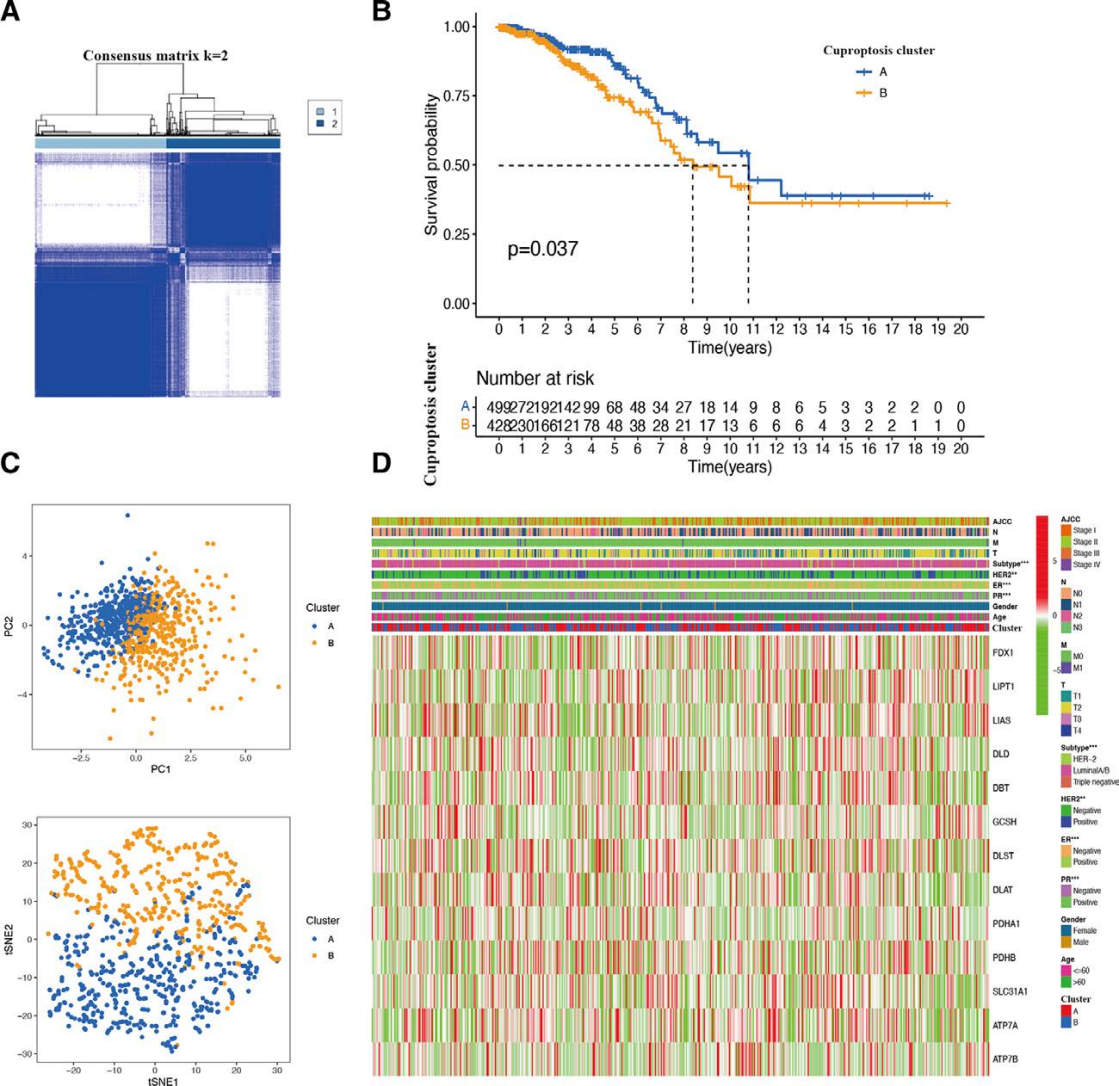
Identification of cuproptosis gene clusters in BR patients. (A) Sample distribution of different clusters. (B) The KM curve of different clusters in BR patients. (C) PCA plot and t-SNE analysis of clusters. (D) The clinical correlation and the expression of the gene associated with cuproptosis among different clusters of BR patients were showed in heatmap. The asterisk represents the statistical *P* value (**P < .01; ***P < .001). BR = breast cancer.

According to 13 identified cuproptosis genes expression, BR patients were classified into 2 clusters (cluster A and cluster B) with optimal K = 2 (Fig. 3A). KM survival curves indicated that, in comparison to the patients of cluster B, the patients of cluster A exhibited significantly increased survival probability (*P* < .05; Fig. 3B). The PCA and t-SNE (Fig. 3C) analyses clearly distinguished the BR samples in the 2 clusters. In addition, the heatmap of clinical characteristics and curoptosis gene clusters confirmed that the constructed BR clusters were significantly related to PR expression, ER expression, HER2 expression, and BR IHC subtypes (Fig. 3D), which revealed the protective value of cuproptosis gene clusters in BR clinical features.

**Figure 4.**
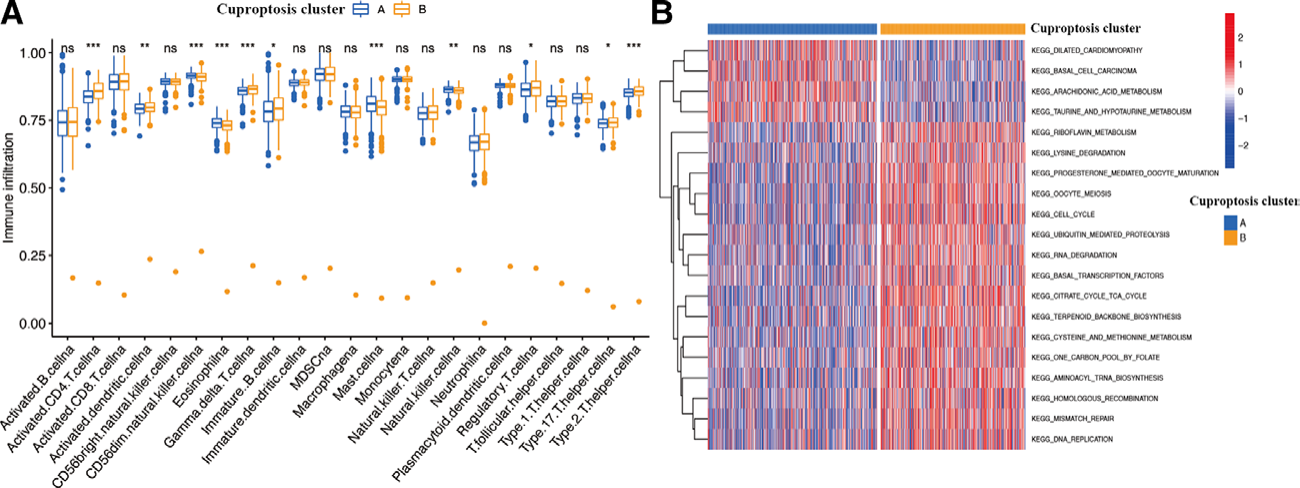
Functional analysis of cuproptosis gene clusters. (A) Differential expression of immune cells among the cuproptosis gene clusters. (B) GSVA enrichment analysis among different clusters.

Due to the latter result, we also attempted to detect the potential mechanism of cuproptosis gene clusters in BR patients. Immune infiltration analyses of the 2 clusters proved that several immune cells, such as type2 T helper cells, type17 T helper cells, regulatory T cells, immature B cells, gamma delta T cells, activated dendritic cells, and activated CD4 T cells, which were significantly less infiltrated in Cluster A (*P* < .05, Fig. 4A). However, other immune cells, including natural killer cells, mast cells, eosinophils, and CD56dim nature killer cells, exhibited a significantly higher infiltration in cluster A (*P* < .05). The GSVA analysis indicated that pathways associated with dilated cardiomyopathy, basal cell carcinoma, arachidonic acid metabolism, and taurine and hypotaurine metabolism were significantly enriched in Cluster A (*P* < .05), whereas riboflavin metabolism, progesterone-mediated oocyte maturation, lysine degradation, oocyte meiosis, and so on, were obviously enriched in Cluster B (*P* < .05, Fig. 4B).

### 3.3. GO and KEGG enrichment analyses of DEGs between clusters

**Figure 5.**
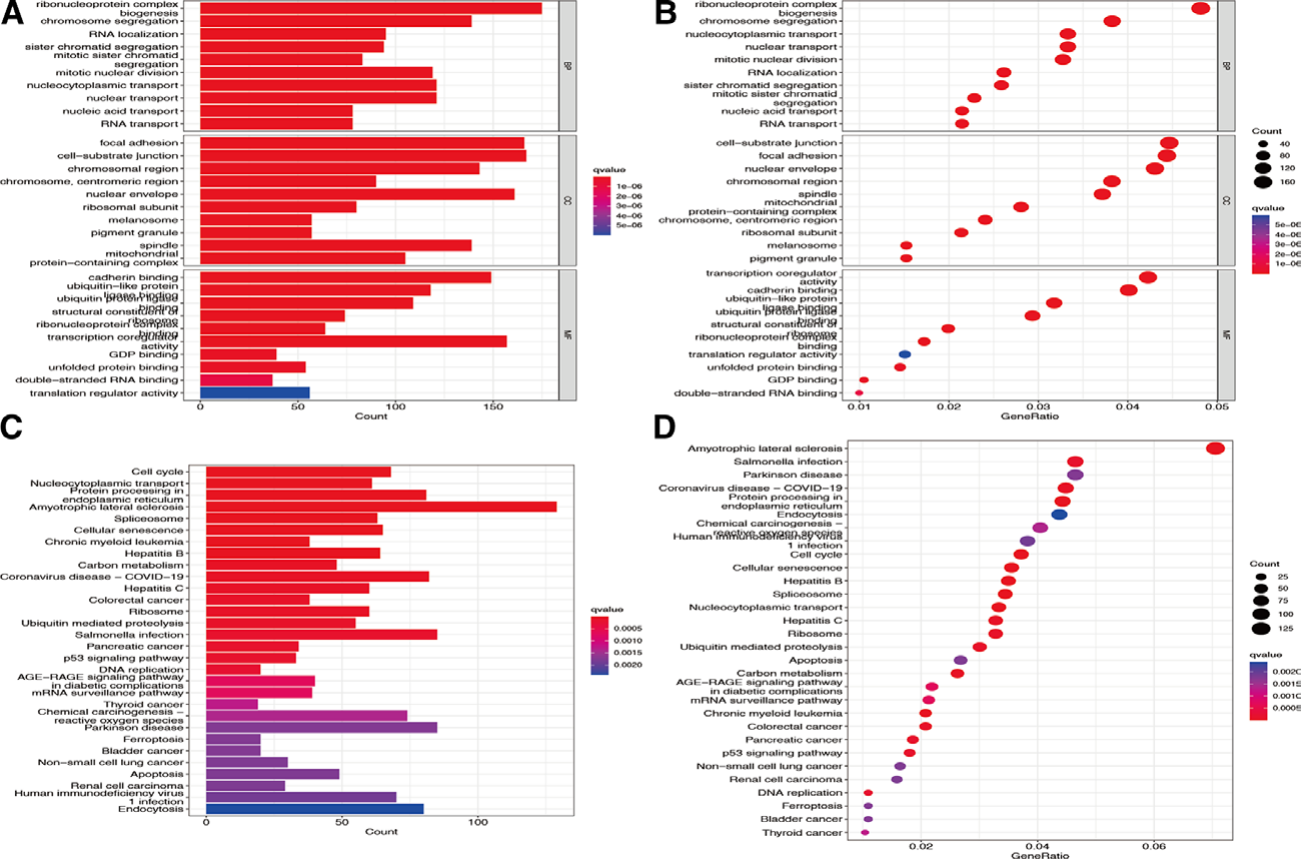
Functional enrichment analysis of DEGs. The GO enrichment terms of hub DEGs in CC, BP, and MF are shown by a bar plot (A) and bubble chart (B). KEGG enrichment terms of DEGs are shown by a bar plot (C) and bubble chart (D). The size of the dot depicts the number of augmented genes. BR = breast cancer, DEGs = differentially expressed genes, GO = Gene ontology, KEGG = Kyoto Encyclopedia of Genes and Genomes.

After screening DEGs between the 2 cuproptosis gene clusters according to FDR < 0.001 standards (Supplementary Table 1, http://links.lww.com/MD/I132), the DEGs identified were constructed for functional analyses. As shown in Figure 5A and B, primary involvement of DEGs in certain critical BPs, such as ribonucleoprotein complex biogenesis, chromosome segregation, and RNA localization, was found. DEGs were also found to be enriched in focal adhesion, cell-substrate junction, chromosomal region, etc in the CC category; ubiquitin-protein ligase binding, ubiquitin-like protein ligase binding, and cadherin binding, etc, in the MF category. Moreover, we also observed tightly connections of DEGs to numerous pathways, such as the cell cycle, nucleocytoplasmic transport, and protein processing in the endoplasmic reticulum, from our KEGG enrichment analysis (Fig. 5C and D).

### 3.4. Prognostic DEGs clusters screening

**Figure 6.**
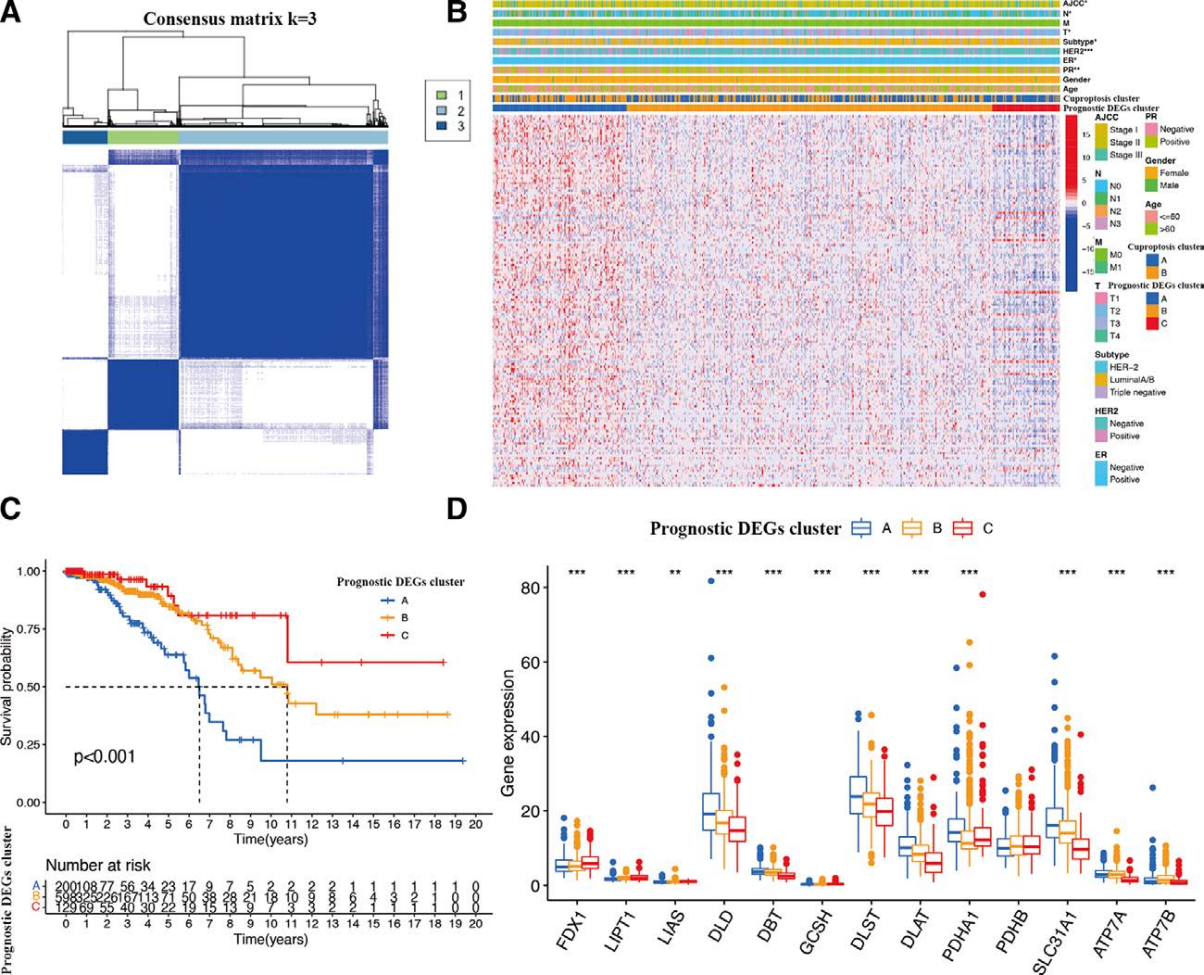
Identification of prognostic DEGs clusters in the patients with BR. (A) Sample distribution of different clusters. (B) The clinical correlation and gene expression among different clusters of BR patients were showed in heatmap. (C) The KM curve of different clusters in BR patients. (D) Differential expression of cuproptosis genes among the prognostic DEGs clusters. The asterisk represents the statistical *P* value (*P < .05; **P < .01; ***P < .001). BR = breast cancer, DEGs = differentially expressed genes.

Based on the univariate Cox regression, 150 prognostic DEGs were identified with a cutoff of *P* < .001 (Supplementary Table 2, http://links.lww.com/MD/I133), and BR patients were classified into 3 clusters (Clusters 1, 2, and 3) by the prognostic DEGs expression levels (Fig. 6A). We observed significant differences in BR AJCC stage, N stage, T stage, IHC subtype, HER2 expression, ER expression, PR expression, and overall survival among the 3 clusters using clinical correlation analyses (Fig. 6B and C). Moreover, cuproptosis genes were significantly and differentially expressed among the 3 clusters, but only PDHB did not show a significant difference (Fig. 6D).

### 3.5. Construction of CAGs risk signature for BR

**Figure 7.**
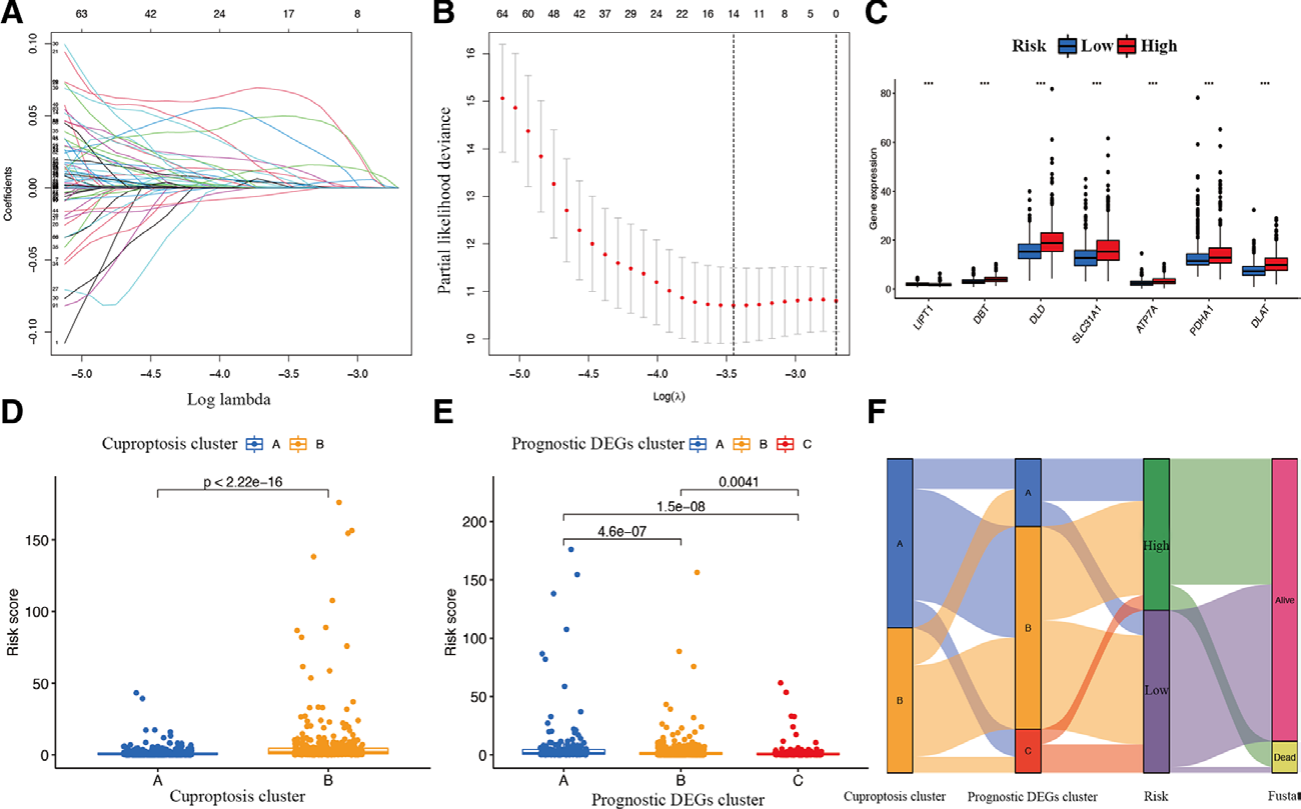
Construction of CAGs risk signature. (A-B) LASSO analysis to determine factors and construct the model. (C) The cuproptosis genes differential expression in the risk signature. (D) The connection between the risk signature and cuproptosis gene clusters. (E) The connection between the risk signature and DEGs clusters. (F) Representative alluvial diagram showing the fustate, signature, and clusters changes. CAGs = cuproptosis-associated genes, DEGs = differentially expressed genes, LASSO = least absolute shrinkage and selection operator.

Prognostic DEGs were further analyzed by the least absolute shrinkage and selection operator analysis (Fig. 7A and B), and 11 hub CAGs, including COPB2, MRPL39, PGK1, PRDX1, PCMT1, MPZL3, LACTB2, HSPH1, DIP2B, DLG3, and NFKBIA, were used to construct the risk signature (Supplementary Table 3, http://links.lww.com/MD/I134). The risk score of each patient with BR was calculated based on the expression levels of the identified CAGs. The connection between the risk signature, cuproptosis gene expression, cuproptosis gene clusters, and DEG clusters is shown in Figure 7C–E. The attribute changes of each BR patient were also visualized in an alluvial diagram (Fig. 7F).

**Figure 8.**
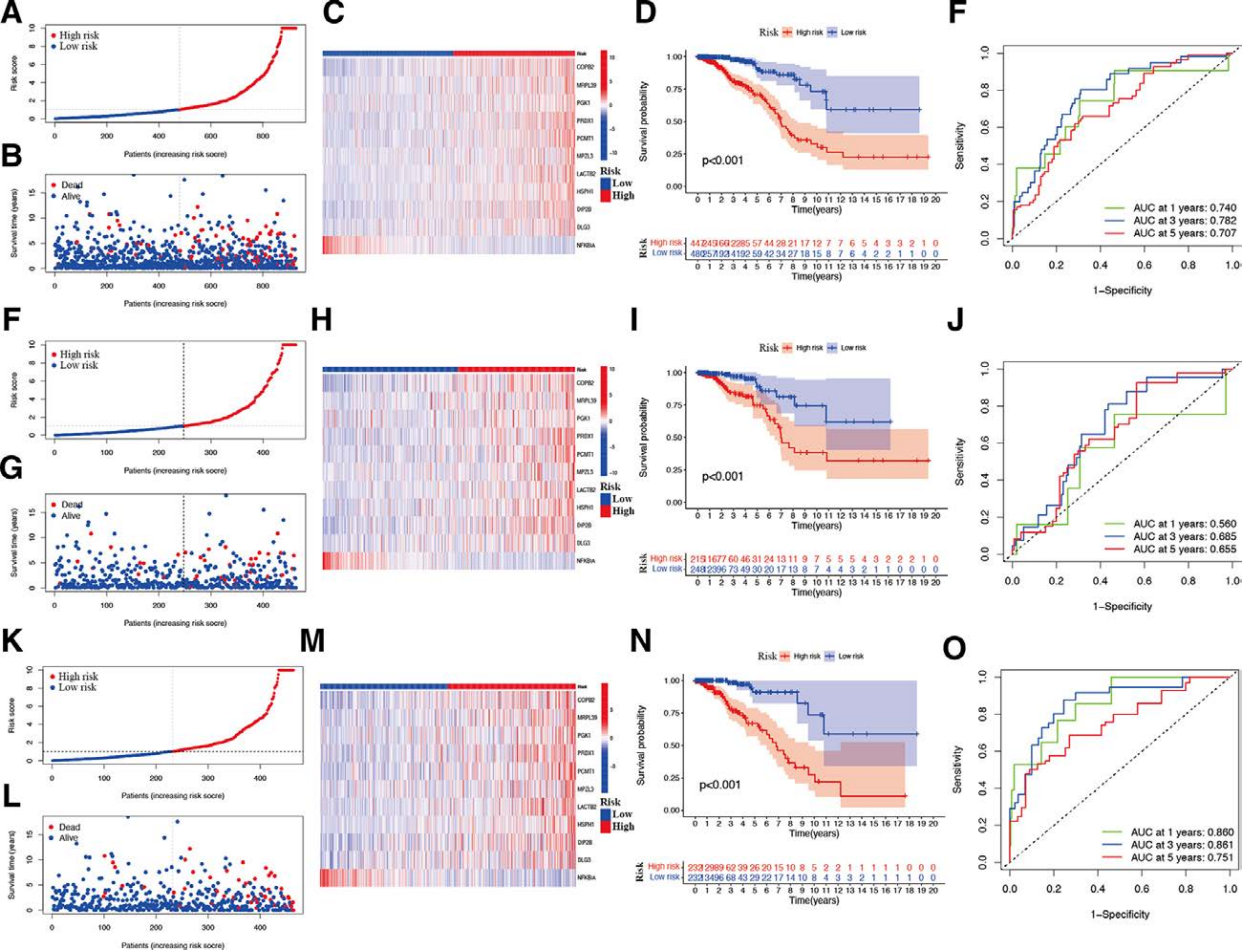
Validation of the risk signature in the overall set, training set, and test set. (A–E) The ROC curve, survival status, and risk score distributions in the overall set. (F–J) The ROC curve, survival status, and risk score distributions in the training set. (K–O) The ROC curve, survival status, and risk score distributions in the training set.

**Figure 9.**
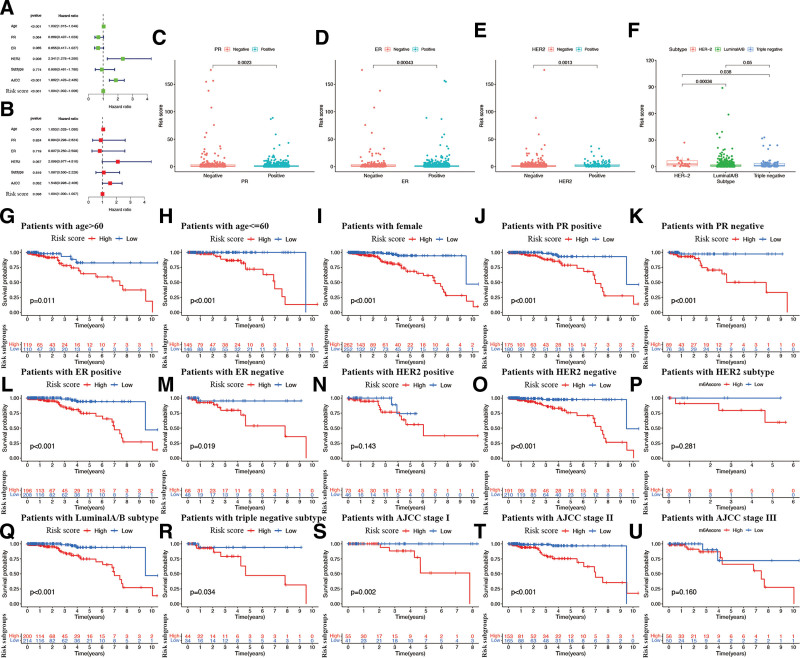
Associations between risk signature and clinicopathological factors. Univariate (A) and multivariate Cox (B) regression of clinicopathological features in BR. Correlations between risk scores and PR expression (C), ER expression (D), HER2 expression (E), and IHC subtype (F). The prognosis of risk signature under the stratifications of (G, H) age > 60 and age ≤ 60; (I) female; (J, K) PR expression; (L, M) ER expression; (N, O) HER2 expression; (P-R) IHC subtype; (S-U) AJCC stage. BR = breast cancer.

As shown in Figure 8A and B, we used the median scores as the threshold to divide the BR patients into 2 groups with low- and high-risk scores. With respect to the relationship between the risk score and CAGs expression level, it was discovered that the patients in the subgroup with high-risk scores showed significantly increased CAGs COPB2, MRPL39, PGK1, PRDX1, PCMT1, MPZL3, LACTB2, HSPH1, DIP2B, and DLG3 expression, whereas only CAGs NFKBIA were expressed in the patients from the high-risk subgroup (Fig. 8C). While exploring the associations between the risk signature and clinical characteristics, the results indicated that, in comparison to the patients from low-risk subgroup, the patients from high-risk subgroup showed significantly low overall survival (*P* < .05; Fig. 8D). The analysis of receiver operating characteristic (ROC) curve indicated that the risk signature had strong predictive accuracy at 1 (ROC = 0.740), 3 (ROC = 0.782), and 5 (ROC = 0.707) years (Fig. 8E). Similar results were retrieved in both the training (Fig. 8F–J) and testing sets (Fig. 8K–O). The analyses of multivariate and univariate Cox regression revealed that the newly identified risk signature was an independent prognostic factor for BR patients (Fig. 9A and B). Interestingly, BR patients diagnosed with negative PR expression (*P* = .0023, Fig. 9C), negative ER expression (*P* = .00043, Fig. 9D), positive HER2 expression (*P* = .0013, Fig. 9E), or HER2 subtype (*P* < .05, Fig. 9F) had significantly higher risk scores. Furthermore, the risk signature’s value for prognosis in patients with BR with diverse clinical features was investigated. As a result, it was revealed that there existed critical significant differences among low- and high-risk signatures in patients aged > 60 years (Fig. 9G), aged ≤ 60 years (Fig. 9H), female patients (Fig. 9I), PR positivity (Fig. 9J), PR negativity (Fig. 9K), ER positivity (Fig. 9L), ER negativity (Fig. 9M), HER2 negativity (Fig. 9O), luminal subtype (Fig. 9Q), triple-negative subtype (Fig. 9R), AJCC stage I (Fig. 9S), and AJCC stage II (Fig. 9T) groups, but not in patients with HER2 positivity (Fig. 9N), HER2 subtype (Fig. 9P), and AJCC stage III (Fig. 9U). In these 2 subgroups, all high-risk signatures displayed a significant survival disadvantage when compared with the low-risk signature.

While exploring the correlation between the hub CAGs and clinical features of BR patients, it was found that CAGs DIP2B and DLG3 were significantly higher expressed in PR and ER positive samples (*P* < .05), whereas CAGs HSPH1, LACTB2, MPZL3, PRDX1, PGK1, and MRPL39 were significantly lower expressed in the same samples (*P* < .05, Supplementary figure 1A and B, http://links.lww.com/MD/I135). With respect to HER2 statue, it was clarified that the expression levels of CAGs COPB2, DIP2B, DLG3, HSPH1, PGK1, and PCMT1 were significantly up-regulated in ER positive samples (*P* < .05), but NFKBIA was significantly down-regulated (*P* < .05, Supplementary figure 1C, http://links.lww.com/MD/I135). Moreover, it was also discovered that CAGs DLG3, PCMT1, and PGK1 were related with BR AJCC stages, specifically, DLG3 and PGK1 were significantly higher expressed in stage IV BR tissues (*P* < .05), and PCMT1 was significantly lower expressed in stage I BR tissues (*P* < .05, Supplementary figure 1D, http://links.lww.com/MD/I135).

### 3.6. Associations with immune landscapes

**Figure 10.**
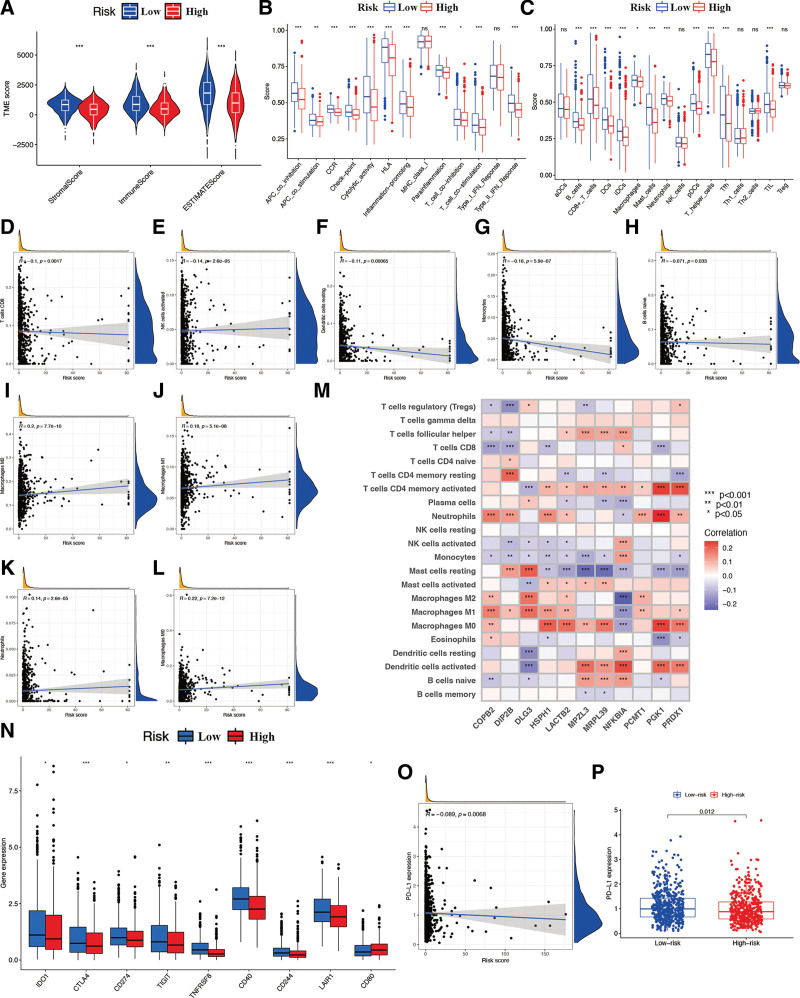
Immune characteristics of risk signature. (A) ESTIMATE analysis of risk signature. Boxplots of scores of immune-associated functions (B) and immune cells (C) in risk signature. CIBERSORT analysis of risk signature (D–L) and identified CAGs (M). (N) Expression of immune checkpoints among 2 risk subgroups in BR patients. Correlation analysis (O) and expression levels (P) of PD-L1 in risk signature. BR = breast cancer, CAGs = cuproptosis-associated genes.

While exploring the associations between the risk signature and cancer immunity, the results showed that stromal, immune, and ESTIMATE scores were all significantly lower in the high-risk subgroup than in the other subgroups (*P* < .05; Fig. 10A). Meanwhile, several components of immune-related pathways and functions, such as type II IFN response, T cell co-stimulation, T cell co-inhibition, parainflammation, inflammation-promoting, HLA, cytolytic activity, checkpoint, CCR, APC co-stimulation, and APC co-inhibition, were significantly inhibited in the patients from high-risk subgroup in comparison to those patients from low-risk subgroup (*P* < .05; Fig. 10B). Meanwhile, the proportions of several immune cell subpopulations, including B cells, CD8 + T cells, DCs, iDCs, macrophages, mast cells, neutrophils, pDCs, T helper cells, Tfh, and TIL, were significantly inhibited in the high-risk subgroup (*P* < .05; Fig. 10C). Furthermore, the CIBERSORT analysis also confirmed that the immune components of CD8 T cells, activated NK cells, resting dendritic cells, monocytes, and naïve B cells were significantly negatively correlated with the constructed risk score (*P* < .05; Fig. 10D–H), however, M0, M1, and M2 macrophages, and neutrophils were positively associated with the risk score (Fig. 10I–L). The association between immune cells and CAGs is shown in Figure 10M. With respect to immune checkpoints, the levels of various immune-associated checkpoints, such as IDO1, CTLA4, CD274, TIGIT, TNFRSF8, CD40, CD244, LAIR1, and CD80, were lower in the high-risk subgroup (Fig. 10N). Moreover, considering the critical role of the immune checkpoint protein PD-L1 in immune progression, we analyzed the correlation between these loci and the risk signature. The results of which indicated that PD-L1 was negatively related to the BR risk signature (Fig. 10O) and was significantly lower in the high-risk subgroup (Fig. 10P).

### 3.7. Mutation profile of risk signature

**Figure 11.**
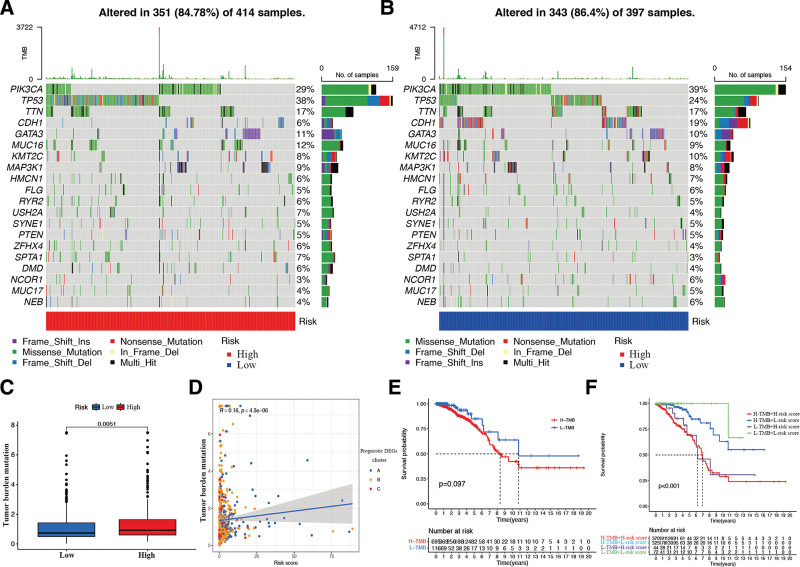
Relationship between risk signature and TMB. Waterfall plot of gene mutation in high (A) and low (B) risk subgroups. (C) The TMB level in risk signature. (D) The correlation between TMB and risk score. (E) The KM curve of high and low TMB subgroups. (F) The KM curve of the combination of risk signature and TMB. TMB = tumor mutational burden.

To further visualize the immunologic nature of the risk signature, the genetic mutation profile of BR patients was investigated. The top 20 genes, which had the highest mutation rates, were identified in the high- (Fig. 11A) and low-risk (Fig. 11B) subgroups. Interestingly, the top 20 mutated genes in both signatures were identical. Meanwhile, 351 and 343 BR samples were found to comprise gene mutations in the high- and low-risk subgroups, respectively. Figure 11C shows that TMB was significantly higher in the high-risk subgroup than in the low-risk subgroup and was positively associated with the calculated risk score (Fig. 11D). Furthermore, as shown in Figure 11D, most BR samples in DEGs Cluster 1 were distributed in the high-risk region, which was consistent with the prognostic value of DEGs clusters in the overall survival of BR patients. Although there was no significant difference between the high and low TMB subgroups in the survival outcome of BR patients (Fig. 11E), the combination of the risk signature and TMB showed significant differences in BR’s overall survival (Fig. 11F), and BR patients in the low TMB and low-risk score subgroups had the best prognosis.

### 3.8. Functional analysis and nomogram construction

**Figure 12.**
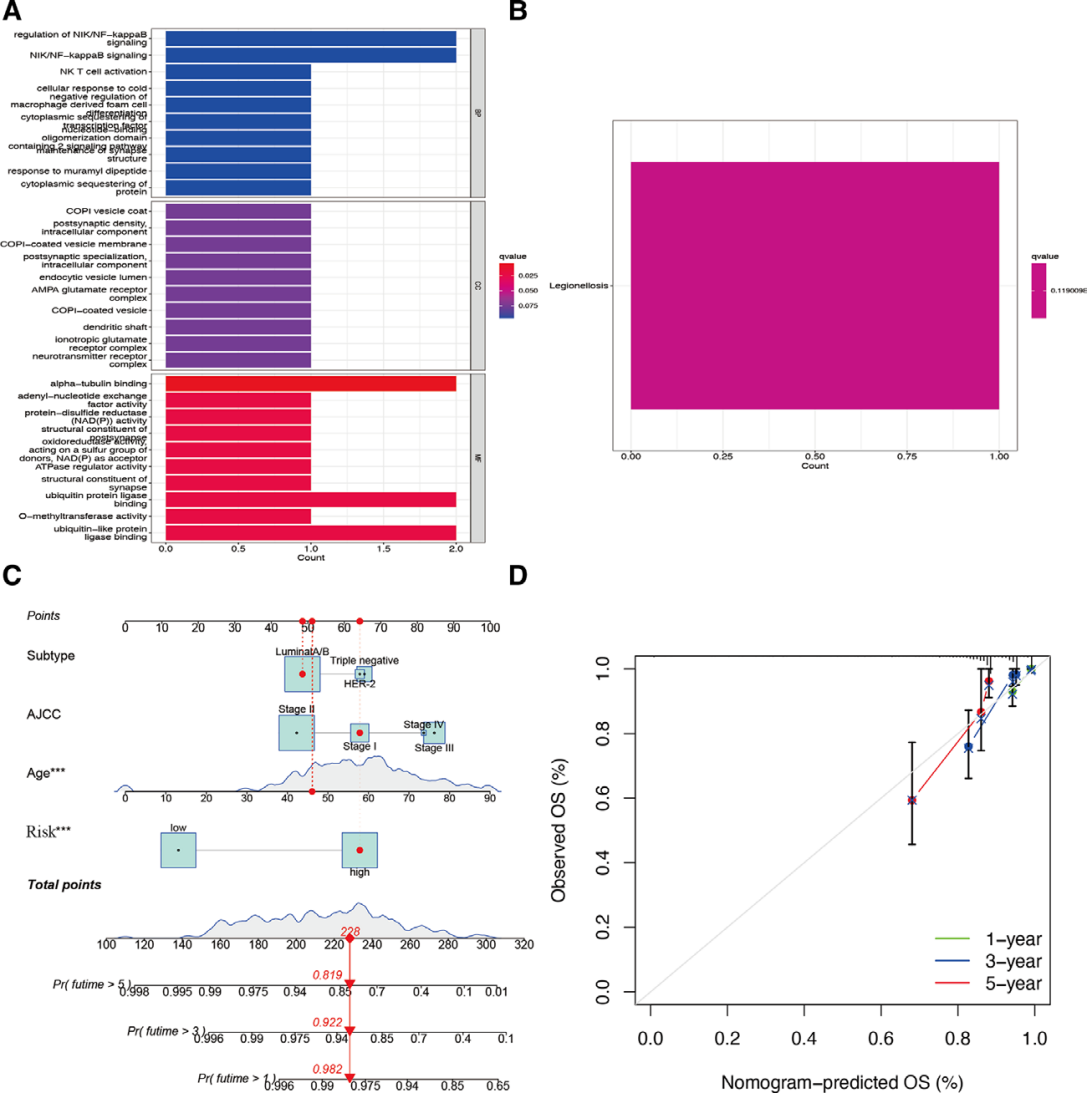
Functional enrichment analysis and nomogram construction. The GO (A) and KEGG (B) enrichment analyses of CAGs. (C) Decision curve analysis of risk signature and other BR clinicopathological features. (D) Nomogram for predicting BR 1-, 3-, and 5-year overall survival. BR = breast cancer, CAGs = cuproptosis-associated genes, GO = gene ontology, KEGG = Kyoto encyclopedia of genes and genomes,

The GO enrichment analysis revealed that hub CAGs found in the risk signature were predominantly augmented by several mechanisms, such as the regulation of NIK/NF-kappa B signaling, COPI vesicle coat, and alpha-tubulin binding (Fig. 12A). Additionally, the KEGG enrichment analysis indicated that CAGs were augmented in the legionellosis pathway (Fig. 12B). These results prompted us to further explore the specific mechanisms underlying hub CAGs. The risk signature was used to construct a nomogram to predict BR patient outcomes (Fig. 12C). Calibration plots indicated that our predictive signature had good conformity between the observed and predicted outcomes at 1, 3, and 5 years (Fig. 12D). Overall, our constructed risk signature was associated with BR prognosis and might prove to be a valuable tool for the clinical management of patients.

### 3.9. Drug sensitivity

**Figure 13.**
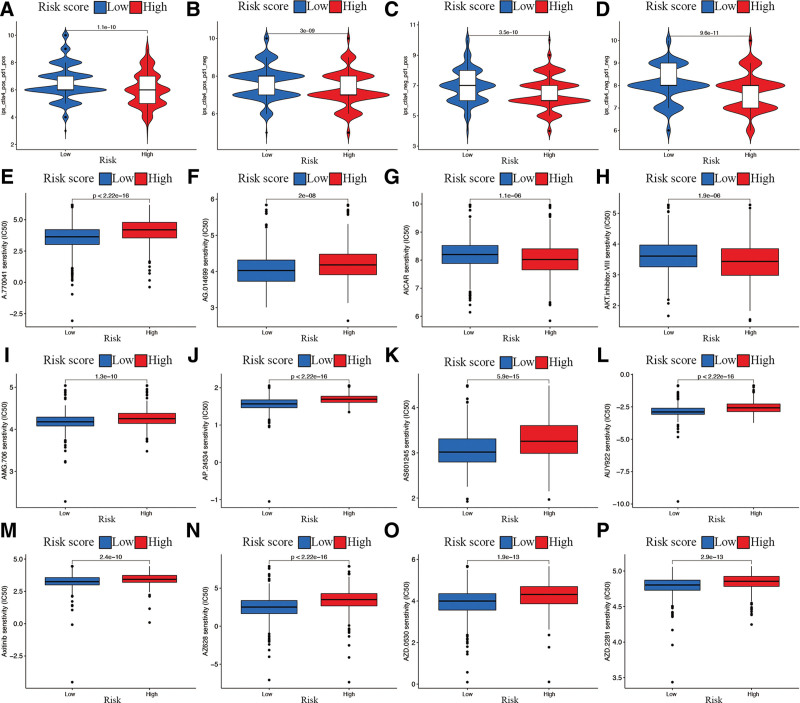
Drug sensitivity analysis. (A–D) Correlation analysis between IPS of anti- CTLA-4, anti-PD-1 blocker, and risk signature. (E–P) Drug sensitivity analysis of the top 12 immunotherapeutic drugs solely showed a significant IC50 difference among the 2 risk subgroups. IPS = immunophenoscore.

To identify the determinants of cancer immunogenicity, anti-CTLA-4 and anti-PD-1 antibody responses were predicted using the IPS.^[16]^ As a result, IPS, IPS-CTLA4, IPS- PD1, and IPS-PD1-CTLA4 blocker scores were all significantly lower in the high-risk subgroup (*P* < .05; Fig. 13A–D), which indicated a worse immunotherapeutic benefit for BR patients in the high-risk subgroup. The results of the drug sensitivity comparison of the top 12 immunotherapeutic drugs, which were most sensitive to the risk signature, are displayed in Figure 13E–P. These results indicate that our risk signature could potentially be applied in further immunotherapy response studies and for precise medication potentiation in patients with BR.

## 4. Discussion

Although next-generation sequencing technology has resulted in the discovery of various biomarkers for BR, there still exists a need for novel markers that are more closely associated with the early detection and prognosis of BR. Cuproptosis, a novel, uncharacterized cell death mechanism that is significantly correlated with human cell death, has potential therapeutic value for treating cancer.^[8]^ However, its role in BR is poorly understood and a CAGs risk signature has not been reported in BR.

In the present study, patients with BR were classified into different clusters based on the expression of 13 identified cuproptosis genes and DEGs. The survival analysis indicated that both types of gene clusters were significantly associated with the overall survival of patients with BR, which encouraged us to further explore the prognostic value of CAGs in BR patients. Next, 11 hub CAGs, including COPB2, MRPL39, PGK1, PRDX1, PCMT1, MPZL3, LACTB2, HSPH1, DIP2B, DLG3, and NFKBIA, were applied to construct a novel prognostic risk signature for BR. The prognostic value of BR was verified using various approaches. Meanwhile, it was confirmed that the identified signature was significantly correlated with PR expression, ER expression, HER2 expression, and IHC subtypes, all of which are considered primary factors affecting the survival rate of BR patients.^[18,19]^ These results revealed that the constructed risk signature not only showed higher accuracy for the prediction of prognosis but could also be used to predict BR IHC subtypes. The nomogram analysis revealed the efficacy of our risk signature in predicting the outcomes of patients with BR.

Based on GSEA, the risk signature was associated with immune-related processes such as NK T cell activation and the negative regulation of macrophage-derived foam cell differentiation. Thus, it is reasonable to assess the predictive value of the CAGs risk signature in immune landscapes. Interestingly, various immune functions (including type II IFN response, T cell co-stimulation, T cell co-inhibition, parainflammation, inflammation-promoting, HLA, cytolytic activity, checkpoint, CCR, APC co-stimulation, and APC co-inhibition) and immune cell subpopulations (including TIL, Tfh, T helper cells, pDCs, neutrophils, mast cells, macrophages, iDCs, DCs, CD8 + T cells, and B cells) were significantly inhibited and infiltrated into the high-risk subgroup. Given the critical roles of these immune cells in stimulating anti-tumor immunity,^[20]^ it is reasonable to conclude that the degree of anti-tumor immunity in patients with BR in the high-risk subgroup was substantially reduced. In addition, the ESTIMATE algorithm demonstrated that stromal, immune cell, and ESTIMATE scores were negatively correlated with the risk score, confirming that stromal and immune cell infiltration was poor in the high-risk subgroup. The CIBERSORT analysis concerning the association between the immune microenvironment and CAGs confirmed that both the risk signature and the hub CAGs identified were significantly connected with several immune cells. Considering the predictive value of the risk signature in overall survival, the immune microenvironment might comprise the potential mechanism of the risk signature in predicting BR progression.

Cancer immunotherapies targeting immune checkpoints have improved the outcomes of various cancers.^[21]^ PD-L1 is a key regulator of immune response.^[22]^ The PD-L1 expression is positively correlated with the clinical outcomes. Clinical trials have demonstrated that, by suppressing the activation of PD-L1 pathway and increasing the function of T cells, the presence of the monoclonal antibodies targeted to PD-1/PD-L1 pathway results in impressive patient outcomes.^[23,24]^ In this study, the significant and differential PD-L1 expression in the low- and high-risk subgroups was also verified and was found to be negatively correlated with the risk score. The levels of various immune checkpoints (including IDO1, CTLA4, CD274, TIGIT, TNFRSF8, CD40, CD244, LAIR1, and CD80) were significantly higher in the low-risk subgroup than in the other subgroups, suggesting that immune responses were dramatically altered in the high-risk group. This further indicated that our newly constructed risk signature holds the potential to guide chemotherapy drug treatment. This hypothesis was confirmed by the drug sensitivity analysis, which showed that IPS, IPS-CTLA4, IPS- PD1, and IPS-PD1-CTLA4 blockers and various immunotherapeutic drugs were significantly associated with the risk signature, although the specific mechanisms underlying these relationships require further exploration.

Despite the prognostic value of the current risk signature, this study had certain limitations. First, the results from our retrospective study require further confirmation through prospective studies. Second, additional experimental assays are needed to verify and validate our conclusions. In the future, functional studies should be performed to gain mechanistic insight into the role of CAGs in BR progression.

## 5. Conclusions

In the present study, a novel risk signature consisting of 11 CAGs was constructed with high predictive accuracy. This risk signature was shown to be valuable for predicting parameters related to the immune microenvironment and tumor mutations in BR patients. To the best of our knowledge, this is the first report of a CAGs signature of cancer. These results also provide a novel basis for understanding the specific effects of CAGs on BR. Therefore, this study contributes significantly to the literature and can contribute to improvements in outcomes and individualized treatments for patients with BR.

## Author contributions

**Conceptualization:** Lijuan Shen.

**Data curation:** Lijuan Shen.

**Formal analysis:** Youwu He.

**Methodology:** Chunhui Fang.

**Project administration:** Haiyan Qiu.

**Writing – original draft:** Qing Chen.

**Writing – review & editing:** Fang Huang and Zhengyuan Wu.

## Supplementary Material
