# Supplementary material for: Cuproptosis-associated genes and immune microenvironment characterization in breast cancer
Source: Medicine (Baltimore). 2022 Dec 16;101(50):e32301. doi: 10.1097/MD.0000000000032301 (PMC9771175; doi:10.1097/MD.0000000000032301)
Supplement: Supplementary file 1 [file medi-101-e32301-s001.pdf]

Table 1. DEGs between the two cuproptosis gene clusters.

| ID | logFC | adj.P.Val |
|---|---|---|
| DLD | 8.15459636 | 3.77E-90 |
| DLAT | 4.21188735 | 4.77E-58 |
| SLC31A1 | 6.9415259 | 1.31E-57 |
| RINT1 | 1.9722677 | 1.20E-51 |
| SLC25A13 | 3.73471871 | 1.16E-47 |
| LRPPRC | 8.40346399 | 1.46E-43 |
| MRPL19 | 2.18406974 | 1.05E-41 |
| DBF4 | 1.91064128 | 8.33E-41 |
| SRPK1 | 3.31559486 | 6.42E-40 |
| PSMC2 | 3.07730756 | 6.49E-40 |
| NUP205 | 5.25094067 | 7.78E-40 |
| GART | 4.32078361 | 1.53E-39 |
| ORC5 | 1.80747211 | 5.16E-39 |
| ZW10 | 3.42444187 | 7.81E-39 |
| PSME4 | 7.56177846 | 1.71E-38 |
| CENPN | 1.38522197 | 3.06E-37 |
| NAA50 | 10.2480257 | 1.00E-35 |
| ACTR2 | 24.3018901 | 1.27E-35 |
| FAM98A | 3.33148221 | 5.47E-35 |
| IMMT | 7.22464977 | 7.64E-35 |
| PNPT1 | 3.25580658 | 1.60E-34 |
| PALM | -4.3649634 | 2.18E-34 |
| PUS7 | 3.05229051 | 2.64E-34 |
| PI4K2B | 3.53962911 | 8.11E-34 |
| GMPS | 5.04326487 | 8.60E-34 |
| CTDSP1 | -8.3199596 | 2.24E-33 |
| PNO1 | 3.0799025 | 4.06E-33 |

| | | |
|---|---|---|
| MTIF2 | 3.74627356 | 5.28E-33 |
| UBE3C | 3.94463758 | 5.52E-33 |
| NRDC | 5.08691862 | 1.18E-32 |
| WDR43 | 4.57154201 | 1.52E-32 |
| NAA15 | 2.48653844 | 1.69E-32 |
| SCYL2 | 3.47915435 | 3.28E-32 |
| MSH2 | 1.767045 | 4.18E-32 |
| NCAPD3 | 1.61737813 | 5.02E-32 |
| EXO1 | 2.77306525 | 5.02E-32 |
| TMEM204 | -5.858242 | 5.33E-32 |
| NAE1 | 3.23720747 | 6.53E-32 |
| STIL | 1.70415188 | 7.97E-32 |
| ABTB1 | -2.1639769 | 1.02E-31 |
| URB2 | 1.82626968 | 1.04E-31 |
| XPOT | 8.00477331 | 1.10E-31 |
| PATL1 | 5.09811567 | 1.16E-31 |
| DNAJC2 | 1.58942262 | 2.60E-31 |
| HSPA8 | 91.6279784 | 2.63E-31 |
| CUL2 | 2.76304065 | 5.91E-31 |
| MSH6 | 3.33415067 | 2.57E-30 |
| SHCBP1 | 1.44767619 | 2.65E-30 |
| RDX | 3.66204852 | 3.12E-30 |
| CHEK1 | 2.2572832 | 3.47E-30 |
| ABCE1 | 3.71747231 | 5.35E-30 |
| NUP155 | 3.0214292 | 5.35E-30 |
| TNS2 | -4.3205263 | 6.06E-30 |
| NIPA2 | 2.0778612 | 6.85E-30 |
| XPO5 | 3.90120169 | 6.99E-30 |
| MTCH2 | 10.6372926 | 7.78E-30 |

| | | |
|---|---|---|
| CDK8 | 2.10322428 | 8.19E-30 |
| NCBP1 | 3.30100319 | 9.74E-30 |
| SUV39H2 | 1.9658342 | 1.30E-29 |
| UCHL5 | 2.56267894 | 1.75E-29 |
| MTPN | 14.4234615 | 1.91E-29 |
| DDX21 | 10.5933405 | 2.03E-29 |
| PEX13 | 2.40392748 | 2.63E-29 |
| MRS2 | 2.11367736 | 2.75E-29 |
| COA7 | 2.79604533 | 3.84E-29 |
| LOXL1 | -8.7629077 | 6.16E-29 |
| EPRS1 | 19.2266223 | 9.61E-29 |
| FBXO45 | 2.42270655 | 1.32E-28 |
| ECT2 | 5.83963847 | 1.54E-28 |
| EFEMP2 | -4.9121467 | 1.66E-28 |
| NCAPG2 | 2.82865377 | 2.01E-28 |
| RFWD3 | 2.90595656 | 2.47E-28 |
| ARCN1 | 14.5132586 | 3.70E-28 |
| CASP3 | 4.23175791 | 4.36E-28 |
| DDIAS | 1.09492706 | 5.49E-28 |
| CIRBP | -19.591151 | 5.86E-28 |
| MELK | 5.61346694 | 6.03E-28 |
| GDI2 | 28.1818154 | 6.80E-28 |
| SLC30A6 | 2.33452881 | 6.84E-28 |
| NOL10 | 2.3998472 | 8.72E-28 |
| TGFB1I1 | -2.7138373 | 9.41E-28 |
| GTPBP4 | 6.12367699 | 9.89E-28 |
| FYTTD1 | 3.46641268 | 9.89E-28 |
| DHTKD1 | 8.74960971 | 9.89E-28 |
| SMC2 | 2.83716428 | 1.08E-27 |

| | | |
|---|---|---|
| CDC25A | 1.5146468 | 1.22E-27 |
| EHD2 | -11.612971 | 1.38E-27 |
| TOMM70 | 6.78605156 | 1.55E-27 |
| CHAC2 | 1.29852721 | 1.90E-27 |
| NCAPG | 2.43202136 | 2.21E-27 |
| KCMF1 | 2.22751304 | 2.37E-27 |
| RAD23B | 13.9347312 | 2.45E-27 |
| MTHFD2 | 3.28743609 | 2.54E-27 |
| CEBPZ | 3.4343909 | 2.86E-27 |
| TTK | 2.9502044 | 2.88E-27 |
| HMMR | 2.1614195 | 3.56E-27 |
| BAZ1B | 6.01327444 | 3.68E-27 |
| PLCH1 | 1.8731569 | 3.89E-27 |
| MCCC1 | 3.41782563 | 3.92E-27 |
| CUL1 | 4.64046563 | 4.07E-27 |
| GINS1 | 2.76553107 | 5.77E-27 |
| KIF18A | 1.27069307 | 1.52E-26 |
| PITRM1 | 2.09061324 | 1.65E-26 |
| SYNCRIP | 6.40276427 | 1.80E-26 |
| LRRC40 | 1.87357261 | 1.91E-26 |
| TRMT6 | 1.95392981 | 2.12E-26 |
| NUDCD1 | 1.52290187 | 2.14E-26 |
| PSMD7 | 3.26066036 | 2.31E-26 |
| CMPK1 | 11.4887151 | 2.43E-26 |
| TMEM123 | 42.1319611 | 2.45E-26 |
| PDIA6 | 17.3940179 | 2.62E-26 |
| TUBA1C | 6.02212101 | 3.43E-26 |
| FAM83D | 6.18306472 | 3.81E-26 |
| DDX10 | 1.18371831 | 5.28E-26 |

| | | |
|---|---|---|
| COG5 | 1.84468676 | 6.96E-26 |
| PSMD14 | 2.73896631 | 7.71E-26 |
| MTFR2 | 1.20597004 | 8.10E-26 |
| VTA1 | 2.46620356 | 1.04E-25 |
| RAB1A | 11.1509831 | 1.04E-25 |
| ORC1 | 1.5131546 | 1.31E-25 |
| YES1 | 6.98161036 | 1.31E-25 |
| CSE1L | 18.0740507 | 1.31E-25 |
| XPO1 | 2.14172928 | 1.37E-25 |
| CHCHD3 | 4.07864239 | 1.79E-25 |
| ANLN | 5.76245508 | 1.79E-25 |
| BUB1 | 3.09322239 | 1.94E-25 |
| KIF4A | 3.62487404 | 2.25E-25 |
| CBX7 | -1.7791098 | 2.51E-25 |
| ME2 | 1.08199919 | 2.59E-25 |
| CENPL | 1.04331975 | 3.06E-25 |
| USP39 | 3.9029356 | 3.20E-25 |
| UBE2A | 3.24852679 | 3.20E-25 |
| FIGNL1 | 1.43193466 | 3.20E-25 |
| HSPA12B | -1.9308111 | 3.59E-25 |
| KPNA3 | 3.54352902 | 3.97E-25 |
| CROCC | -1.4539771 | 3.97E-25 |
| SGO2 | 1.08924659 | 4.15E-25 |
| TSR1 | 2.4833066 | 4.26E-25 |
| HDAC7 | -2.5004178 | 4.28E-25 |
| TFB2M | 4.90146514 | 4.31E-25 |
| FH | 14.5352851 | 4.50E-25 |
| MAPRE1 | 16.734843 | 4.57E-25 |
| NAA25 | 1.21213853 | 4.61E-25 |

| | | |
|---|---|---|
| DHX9 | 8.54188388 | 5.05E-25 |
| ZC3H15 | 5.75548383 | 5.68E-25 |
| TPX2 | 14.4231599 | 6.30E-25 |
| R3HDM1 | 1.65483609 | 6.40E-25 |
| GCC1 | 1.89454348 | 6.50E-25 |
| DEPDC1 | 1.64700373 | 6.50E-25 |
| IGFBP4 | -165.07868 | 8.06E-25 |
| CCT4 | 14.6308766 | 8.06E-25 |
| ASPM | 2.17177981 | 8.30E-25 |
| LIN9 | 1.29691247 | 1.09E-24 |
| SMC1A | 5.22900369 | 1.12E-24 |
| PHF6 | 1.73438721 | 1.24E-24 |
| ZWILCH | 1.41318516 | 1.24E-24 |
| WDR3 | 1.74044742 | 1.33E-24 |
| USP16 | 2.32309314 | 1.58E-24 |
| CUL5 | 1.80114443 | 1.96E-24 |
| DDX1 | 2.66930228 | 2.18E-24 |
| RHEB | 6.05202533 | 2.18E-24 |
| EIF2S2 | 11.4171768 | 2.21E-24 |
| ATP2C1 | 4.7956329 | 3.08E-24 |
| MCM10 | 2.31317644 | 3.16E-24 |
| BUB1B | 2.11813703 | 3.24E-24 |
| MAGT1 | 5.99508783 | 3.24E-24 |
| DDX18 | 3.10669937 | 3.38E-24 |
| CCNA2 | 5.53212981 | 3.38E-24 |
| MAD2L1 | 2.70929654 | 3.63E-24 |
| TNPO3 | 4.67273545 | 3.98E-24 |
| RAB10 | 11.6359168 | 3.98E-24 |
| PODN | -6.1662707 | 4.32E-24 |

| | | |
|---|---|---|
| KIF23 | 2.37780729 | 4.42E-24 |
| PDHA1 | 4.86238471 | 4.80E-24 |
| DLGAP5 | 3.23009043 | 5.34E-24 |
| CKAP2 | 3.99229632 | 5.42E-24 |
| TARS1 | 8.48314014 | 6.02E-24 |
| ORC6 | 1.26016939 | 7.58E-24 |
| MASTL | 1.82568034 | 8.51E-24 |
| GOLT1B | 4.83821555 | 9.88E-24 |
| YME1L1 | 7.12224881 | 1.07E-23 |
| AHCTF1 | 2.9061539 | 1.19E-23 |
| DSCC1 | 2.91364274 | 1.21E-23 |
| MOB1A | 5.98260453 | 1.31E-23 |
| AUNIP | 1.16403199 | 1.32E-23 |
| NOP53 | -23.25727 | 1.55E-23 |
| DCUN1D1 | 1.57436007 | 1.56E-23 |
| USP6NL | 1.15101033 | 1.56E-23 |
| ACTR3 | 5.21588903 | 1.76E-23 |
| ARHGEF40 | -1.9270204 | 1.77E-23 |
| CNOT1 | 5.49944382 | 1.94E-23 |
| MARS1 | 3.63257542 | 1.97E-23 |
| MORC4 | 2.51706382 | 2.04E-23 |
| RRM2 | 4.29459265 | 2.17E-23 |
| SKA3 | 2.35935247 | 2.17E-23 |
| PNPLA8 | 1.83010483 | 2.46E-23 |
| PHF1 | -4.8180662 | 2.46E-23 |
| UBQLN1 | 6.2989369 | 2.57E-23 |
| GPR180 | 1.12714233 | 2.79E-23 |
| TRIM8 | -6.22335 | 3.19E-23 |
| CYCS | 7.75435696 | 3.19E-23 |

| | | |
|---|---|---|
| YWHAG | 18.9941926 | 3.19E-23 |
| FOXS1 | -1.4333263 | 3.29E-23 |
| RRP36 | 5.84443656 | 4.10E-23 |
| ANKIB1 | 3.20609622 | 4.69E-23 |
| DNA2 | 1.12331268 | 4.80E-23 |
| KIF14 | 1.25981582 | 5.31E-23 |
| COPB2 | 3.88033091 | 5.31E-23 |
| GFM1 | 1.80075035 | 6.28E-23 |
| USP9X | 4.56578461 | 6.63E-23 |
| STARD7 | 9.59727766 | 6.78E-23 |
| RACGAP1 | 5.09190457 | 8.13E-23 |
| CARD19 | -4.1306383 | 9.10E-23 |
| PDSS1 | 1.5272171 | 9.60E-23 |
| MED14 | 2.33420344 | 9.62E-23 |
| VPS54 | 2.28501417 | 9.64E-23 |
| AFG3L2 | 2.30001542 | 1.20E-22 |
| SEC24A | 2.20635228 | 1.20E-22 |
| MAPK14 | 2.2554601 | 1.24E-22 |
| CCT8 | 14.3806594 | 1.24E-22 |
| ESRP1 | 13.1356786 | 1.25E-22 |
| CENPE | 1.26901074 | 1.29E-22 |
| SPDL1 | 1.0877379 | 1.35E-22 |
| NUS1 | 2.5613046 | 1.40E-22 |
| STRN | 1.55568073 | 1.65E-22 |
| PSMD1 | 1.83754974 | 1.65E-22 |
| PRKCI | 3.19457765 | 2.33E-22 |
| ATP13A3 | 3.55005867 | 2.38E-22 |
| LBR | 4.06461389 | 2.69E-22 |
| CPSF3 | 3.68103074 | 2.95E-22 |

| | | |
|---|---|---|
| CEP55 | 5.07787982 | 3.75E-22 |
| API5 | 5.30796122 | 3.89E-22 |
| NIP7 | 1.79541745 | 3.91E-22 |
| CALCOCO1 | -2.803558 | 4.08E-22 |
| KIF15 | 1.40872819 | 4.25E-22 |
| SERBP1 | 10.5010393 | 4.34E-22 |
| NBN | 6.34664516 | 5.40E-22 |
| VBP1 | 6.88411673 | 5.58E-22 |
| MRPL39 | 5.10984205 | 6.37E-22 |
| PSMD10 | 5.09216139 | 7.03E-22 |
| PTCD3 | 1.24493738 | 7.71E-22 |
| C1QTNF6 | -3.0024771 | 7.82E-22 |
| NCKAP1 | 2.47452503 | 7.87E-22 |
| KIF11 | 3.89316141 | 7.89E-22 |
| DONSON | 2.07197499 | 8.23E-22 |
| ACTR6 | 2.16134837 | 8.27E-22 |
| CENPA | 2.9900622 | 8.72E-22 |
| NUP160 | 2.72720967 | 9.45E-22 |
| TCP1 | 12.3887655 | 9.67E-22 |
| CKAP2L | 1.22184417 | 9.78E-22 |
| EZH2 | 3.29482666 | 1.04E-21 |
| TMPO | 5.00725459 | 1.05E-21 |
| CKAP5 | 6.84239345 | 1.26E-21 |
| DESI2 | 5.52643665 | 1.28E-21 |
| NOP58 | 5.06666655 | 1.32E-21 |
| TDG | 1.58215165 | 1.32E-21 |
| GARS1 | 2.53106759 | 1.51E-21 |
| MAIP1 | 1.92937273 | 1.57E-21 |
| C5orf22 | 2.19379697 | 1.74E-21 |

| | | |
|---|---|---|
| MPHOSPH10 | 2.84440844 | 1.86E-21 |
| CBLL1 | 2.02805514 | 1.87E-21 |
| USP28 | 1.48677109 | 1.98E-21 |
| TAF2 | 3.34438247 | 2.01E-21 |
| FANCI | 1.71167638 | 2.10E-21 |
| RAD51AP1 | 3.73340888 | 2.30E-21 |
| POLR2B | 6.47731472 | 2.52E-21 |
| HAUS6 | 1.32379317 | 2.56E-21 |
| MARCHF2 | -3.9727022 | 2.67E-21 |
| LARP4B | 2.38393411 | 2.85E-21 |
| NSUN2 | 3.86799067 | 3.03E-21 |
| ATP11C | 1.49526141 | 3.25E-21 |
| TXNDC9 | 2.11032959 | 3.64E-21 |
| FAF1 | 1.92536345 | 3.65E-21 |
| LRRC58 | 2.74171061 | 3.65E-21 |
| POLL | -1.2052022 | 4.25E-21 |
| MFSD14B | 4.22332209 | 4.35E-21 |
| UBA2 | 7.05095474 | 4.66E-21 |
| IDE | 1.0062181 | 5.50E-21 |
| NDC1 | 4.40258713 | 5.63E-21 |
| FBXO5 | 1.72558308 | 5.67E-21 |
| CDCA8 | 6.26486929 | 7.49E-21 |
| PEX3 | 1.30283485 | 8.13E-21 |
| LAMB2 | -16.123383 | 8.26E-21 |
| DPM1 | 10.6116134 | 8.34E-21 |
| FAM136A | 4.5494568 | 8.70E-21 |
| PSMD2 | 14.6770037 | 9.16E-21 |
| COX4I2 | -1.6492976 | 9.29E-21 |
| ETF1 | 6.21522598 | 1.07E-20 |

| | | |
|---|---|---|
| AKIRIN1 | 6.10599324 | 1.09E-20 |
| HTRA1 | -58.539812 | 1.15E-20 |
| PLAA | 2.84855875 | 1.21E-20 |
| TMEM115 | -5.2776344 | 1.22E-20 |
| EML4 | 3.11062254 | 1.37E-20 |
| CLINT1 | 3.28896363 | 1.39E-20 |
| FDX1 | 1.45128054 | 1.58E-20 |
| SKA1 | 1.94070475 | 1.76E-20 |
| RTCA | 2.60406648 | 1.77E-20 |
| MRPS10 | 6.92702834 | 1.80E-20 |
| GNG11 | -3.8119866 | 1.90E-20 |
| VPS35 | 5.73636176 | 1.94E-20 |
| DNM1L | 3.03493295 | 2.17E-20 |
| ASCC3 | 2.11019464 | 2.17E-20 |
| CRTC1 | -1.0250796 | 2.33E-20 |
| MCM6 | 7.20697995 | 2.42E-20 |
| PDCD10 | 3.51743626 | 2.61E-20 |
| UBE4A | 2.30479233 | 2.75E-20 |
| CCNC | 3.17787611 | 2.88E-20 |
| MKI67 | 4.97557978 | 3.11E-20 |
| TNK2 | -2.4304241 | 3.17E-20 |
| SMC6 | 1.31826753 | 3.24E-20 |
| CCNB2 | 5.40839392 | 3.40E-20 |
| RPA1 | 4.50788072 | 3.76E-20 |
| LCLAT1 | 1.01170273 | 3.84E-20 |
| COPA | 16.4051323 | 3.93E-20 |
| ZPR1 | 1.3016235 | 4.02E-20 |
| NCAPH | 1.49411029 | 4.13E-20 |
| SRP72 | 7.02137427 | 4.21E-20 |

| | | |
|---|---|---|
| LMBR1 | 1.59642846 | 4.22E-20 |
| TAF5L | 1.65119978 | 4.60E-20 |
| GNPNAT1 | 2.77749138 | 4.67E-20 |
| C12orf4 | 1.3585054 | 4.71E-20 |
| PTPN11 | 5.64080374 | 4.80E-20 |
| NDC80 | 3.03442976 | 4.96E-20 |
| UTP4 | 2.42150652 | 4.98E-20 |
| TRMT10C | 4.3185399 | 5.09E-20 |
| ADGRG6 | 1.83612722 | 5.34E-20 |
| ACBD4 | -1.5990407 | 5.49E-20 |
| TEX261 | 4.76788574 | 6.02E-20 |
| PLK4 | 1.28138683 | 6.26E-20 |
| ZFR | 3.98543906 | 6.44E-20 |
| CCT6A | 21.4707264 | 6.72E-20 |
| KIF21A | 1.35538688 | 6.93E-20 |
| PPAT | 1.83337034 | 7.07E-20 |
| BTF3L4 | 2.47539402 | 7.12E-20 |
| SLC27A1 | -1.6596283 | 7.32E-20 |
| POLR1B | 1.21707607 | 7.67E-20 |
| MVP | -9.5560021 | 7.75E-20 |
| MDH1 | 6.07262853 | 7.80E-20 |
| CALHM2 | -2.5898009 | 8.03E-20 |
| SSR1 | 5.06772085 | 8.06E-20 |
| IFITM2 | -32.998455 | 8.52E-20 |
| PRKAR2A | 3.23482243 | 8.75E-20 |
| NBL1 | -29.830491 | 8.83E-20 |
| ESF1 | 2.21318962 | 8.87E-20 |
| YWHAQ | 33.6545074 | 8.91E-20 |
| AIFM1 | 1.27006202 | 9.14E-20 |

| | | |
|---|---|---|
| AASDHPPT | 1.82656993 | 9.15E-20 |
| NKAPD1 | 1.36395697 | 9.19E-20 |
| USP10 | 3.78062443 | 9.77E-20 |
| SLC25A5 | 41.3030496 | 9.81E-20 |
| DKC1 | 8.13784087 | 1.05E-19 |
| FOXM1 | 6.18969334 | 1.08E-19 |
| CBFB | 3.17360973 | 1.12E-19 |
| KIF2C | 4.54053114 | 1.14E-19 |
| HYOU1 | 7.90268641 | 1.28E-19 |
| PACC1 | 1.86695377 | 1.29E-19 |
| SELENOI | 2.85568216 | 1.31E-19 |
| IDUA | -1.9210574 | 1.44E-19 |
| ATP2A2 | 5.31048147 | 1.57E-19 |
| MRPL3 | 8.38545507 | 1.65E-19 |
| CLEC14A | -4.340271 | 1.66E-19 |
| MCUR1 | 2.58248351 | 1.78E-19 |
| HEATR1 | 2.62391237 | 1.84E-19 |
| PROSER1 | 2.3900263 | 1.90E-19 |
| CDC123 | 8.87086947 | 1.94E-19 |
| NUDT21 | 4.93274949 | 1.95E-19 |
| UHRF1BP1 | 1.48347467 | 2.05E-19 |
| PPIL1 | 6.17018731 | 2.16E-19 |
| TFAM | 2.87622896 | 2.18E-19 |
| IARS2 | 14.5025523 | 2.24E-19 |
| MEIS3 | -2.3606168 | 2.36E-19 |
| ZC3HAV1 | 2.09306378 | 2.38E-19 |
| KIF20B | 1.20599561 | 2.38E-19 |
| STT3A | 7.30365264 | 2.40E-19 |
| DPAGT1 | 2.45996349 | 2.40E-19 |

| | | |
|---|---|---|
| PODNL1 | -1.5342803 | 2.50E-19 |
| MXRA8 | -22.449391 | 2.60E-19 |
| AGFG1 | 2.59095296 | 2.60E-19 |
| ARL5B | 2.38878072 | 2.69E-19 |
| ATG3 | 1.63015104 | 2.78E-19 |
| VPS26A | 3.49561453 | 2.79E-19 |
| PLK1 | 3.06602613 | 2.97E-19 |
| EI24 | 9.52108039 | 2.98E-19 |
| HSPA9 | 8.71090349 | 3.05E-19 |
| DARS2 | 2.38751927 | 3.16E-19 |
| ZBTB33 | 2.77730099 | 3.29E-19 |
| NMD3 | 3.21040733 | 3.40E-19 |
| DENR | 5.93198837 | 3.40E-19 |
| ERGIC2 | 2.58441586 | 3.40E-19 |
| PRIM2 | 1.17251499 | 3.51E-19 |
| KLF2 | -7.3395954 | 3.64E-19 |
| POLR1A | 1.22937865 | 3.84E-19 |
| HNRNPLL | 1.31436765 | 3.95E-19 |
| LAMP5 | -4.2305529 | 4.05E-19 |
| RRM1 | 7.0691907 | 4.05E-19 |
| SRSF1 | 2.69963424 | 4.13E-19 |
| FARSB | 1.98861933 | 4.50E-19 |
| USP1 | 8.71902776 | 4.50E-19 |
| SMARCC1 | 6.83095037 | 4.86E-19 |
| ALAS1 | 4.26235334 | 4.86E-19 |
| MXD4 | -4.1282076 | 4.95E-19 |
| CDK1 | 6.85574207 | 4.96E-19 |
| ENOX2 | 1.03659889 | 5.05E-19 |
| NISCH | -2.9215939 | 5.35E-19 |

| | | |
|---|---|---|
| UBE2G1 | 2.94727694 | 5.35E-19 |
| ZDHHC20 | 4.95013784 | 5.38E-19 |
| NDUFS1 | 1.98859486 | 5.46E-19 |
| ILF2 | 39.356956 | 5.81E-19 |
| RAD51 | 1.52721449 | 6.16E-19 |
| USO1 | 6.70530116 | 6.45E-19 |
| KNSTRN | 1.95227342 | 6.64E-19 |
| ABCB10 | 1.73309162 | 6.98E-19 |
| TIE1 | -1.7159943 | 8.72E-19 |
| ZNF146 | 9.27205829 | 8.82E-19 |
| PIK3CB | 1.97736775 | 9.00E-19 |
| RPAP3 | 1.48142118 | 9.00E-19 |
| RRP1B | 3.16431197 | 9.86E-19 |
| ITGB5 | -15.163097 | 1.07E-18 |
| GNAI3 | 2.61898538 | 1.10E-18 |
| HUWE1 | 5.81369725 | 1.14E-18 |
| TRIP13 | 3.83582951 | 1.24E-18 |
| STAM | 1.61976248 | 1.25E-18 |
| SUPV3L1 | 1.75590789 | 1.29E-18 |
| GNAI2 | -8.4855949 | 1.32E-18 |
| NUP93 | 1.35755799 | 1.42E-18 |
| CNOT11 | 4.51488008 | 1.42E-18 |
| ESAM | -3.9353706 | 1.43E-18 |
| THAP5 | 1.40365794 | 1.44E-18 |
| RNGTT | 1.17485382 | 1.44E-18 |
| TAX1BP1 | 4.12268743 | 1.47E-18 |
| ATP5F1C | 23.6849192 | 1.48E-18 |
| BBC3 | -1.8829185 | 1.50E-18 |
| LTBP3 | -4.4727658 | 1.55E-18 |

| | | |
|---|---|---|
| LRRC32 | -4.3645311 | 1.59E-18 |
| GTF3C3 | 1.30729237 | 1.64E-18 |
| MTM1 | 1.26391272 | 1.76E-18 |
| MIEF1 | 1.42100646 | 1.78E-18 |
| KCTD20 | 3.85295998 | 1.80E-18 |
| SH3GLB2 | -6.1101145 | 1.84E-18 |
| GMCL1 | 1.65983261 | 1.96E-18 |
| POLE3 | 6.73274874 | 2.01E-18 |
| APBB1 | -1.9702226 | 2.22E-18 |
| ATP6V1A | 5.5799282 | 2.24E-18 |
| CENPF | 5.59432494 | 2.27E-18 |
| MFAP4 | -18.554267 | 2.28E-18 |
| WSB2 | 3.78509381 | 2.39E-18 |
| SMC4 | 3.44690935 | 2.39E-18 |
| MMUT | 4.38528213 | 2.49E-18 |
| ZDHHC1 | -1.2340992 | 2.56E-18 |
| VPS26C | 1.11649904 | 2.63E-18 |
| FMR1 | 1.80086154 | 2.71E-18 |
| SNRNP35 | -1.2620392 | 2.78E-18 |
| TIGD2 | 1.62380816 | 2.97E-18 |
| EPCAM | 39.1621688 | 3.09E-18 |
| NDN | -3.3626921 | 3.21E-18 |
| TOPBP1 | 2.52475217 | 3.34E-18 |
| FBXW4 | -3.5394 | 3.44E-18 |
| NEK2 | 4.29820935 | 3.62E-18 |
| ZBTB7A | -1.9769581 | 3.62E-18 |
| PGK1 | 30.3709723 | 3.63E-18 |
| LHFPL6 | -3.5129806 | 3.77E-18 |
| TAMALIN | -1.4092365 | 3.95E-18 |

| | | |
|---|---|---|
| PHLDB3 | -1.5453668 | 4.01E-18 |
| TIPRL | 5.31338862 | 4.43E-18 |
| MCM4 | 11.2516328 | 4.46E-18 |
| TEX10 | 1.00013453 | 4.81E-18 |
| ZUP1 | 1.07320792 | 4.90E-18 |
| ROBO4 | -1.0557336 | 4.92E-18 |
| BRIX1 | 2.24542771 | 5.14E-18 |
| TFRC | 12.2378169 | 5.32E-18 |
| IGFBP7 | -32.729371 | 5.50E-18 |
| CCSAP | 1.41361162 | 5.53E-18 |
| SOS1 | 2.0830289 | 6.10E-18 |
| LAMP2 | 7.82551089 | 6.29E-18 |
| PAK1IP1 | 5.63088218 | 6.37E-18 |
| CDC5L | 3.46909834 | 6.54E-18 |
| TTC27 | 1.48580059 | 6.99E-18 |
| BIRC2 | 3.55613219 | 7.19E-18 |
| PLAGL2 | 1.5391862 | 7.49E-18 |
| DHX33 | 1.34194881 | 7.64E-18 |
| CAPZA1 | 10.4630337 | 8.24E-18 |
| SLC35A2 | 4.76470547 | 8.30E-18 |
| KPNB1 | 5.66503531 | 8.30E-18 |
| PPP2R5D | 2.82775683 | 8.38E-18 |
| ALG6 | 1.11564128 | 8.38E-18 |
| INTS8 | 2.16556805 | 8.66E-18 |
| NLN | 1.2011301 | 8.91E-18 |
| CBX2 | 3.83829698 | 9.32E-18 |
| CS | 5.39908078 | 9.53E-18 |
| RPP40 | 1.48720249 | 9.55E-18 |
| LTBP4 | -2.8899251 | 9.69E-18 |

| | | |
|---|---|---|
| COP1 | 3.26260523 | 9.96E-18 |
| CLDN5 | -3.0555947 | 1.08E-17 |
| LSG1 | 2.47051475 | 1.15E-17 |
| DNMT3B | 1.26858254 | 1.18E-17 |
| ATP5F1B | 51.7870884 | 1.20E-17 |
| NOLC1 | 9.59146816 | 1.24E-17 |
| SRSF5 | -9.771474 | 1.29E-17 |
| HINT3 | 4.12311691 | 1.29E-17 |
| MED16 | -3.8033299 | 1.30E-17 |
| KIF20A | 3.95591827 | 1.35E-17 |
| ERP44 | 3.12568376 | 1.42E-17 |
| TMED2 | 24.7281875 | 1.53E-17 |
| PRDX1 | 46.489659 | 1.54E-17 |
| UBA6 | 1.38610839 | 1.54E-17 |
| INCENP | 2.14526358 | 1.61E-17 |
| POLR2D | 1.54399909 | 1.61E-17 |
| PTBP3 | 6.11695329 | 1.63E-17 |
| E2F3 | 2.78303168 | 1.70E-17 |
| DTX3 | -2.6988849 | 1.83E-17 |
| GTF3C2 | 1.97815016 | 1.85E-17 |
| CDC7 | 2.09527091 | 1.95E-17 |
| CCNJ | 1.07629035 | 1.98E-17 |
| VRK1 | 2.03803843 | 2.00E-17 |
| CAVIN1 | -28.088677 | 2.03E-17 |
| EIF4G1 | 11.1097546 | 2.06E-17 |
| TXLNG | 2.37552208 | 2.07E-17 |
| CARNMT1 | 1.25710504 | 2.08E-17 |
| P4HA3 | -1.2172902 | 2.13E-17 |
| UGP2 | 4.97786468 | 2.30E-17 |

| | | |
|---|---|---|
| IPMK | 1.07747301 | 2.36E-17 |
| COQ4 | -3.2001641 | 2.36E-17 |
| CMTM3 | -6.5214453 | 2.42E-17 |
| PDHX | 3.82331791 | 2.47E-17 |
| KPNA2 | 11.1510628 | 2.50E-17 |
| RAD1 | 1.13949265 | 2.50E-17 |
| GADD45B | -13.441779 | 2.55E-17 |
| DHX36 | 1.45070032 | 2.57E-17 |
| STX6 | 2.39770601 | 2.58E-17 |
| HEATR3 | 1.47670771 | 2.64E-17 |
| E2F8 | 1.14913887 | 2.64E-17 |
| RPS15 | -48.268671 | 2.65E-17 |
| GAS6 | -6.6820602 | 2.79E-17 |
| ACBD3 | 6.6167824 | 2.81E-17 |
| XPO4 | 1.00930994 | 3.01E-17 |
| TM9SF3 | 7.60666009 | 3.09E-17 |
| CYRIB | 3.51576415 | 3.13E-17 |
| RFC4 | 3.51514214 | 3.18E-17 |
| UBE2D1 | 1.78580061 | 3.39E-17 |
| C6orf62 | 16.6248404 | 3.46E-17 |
| GGT5 | -3.8665087 | 3.46E-17 |
| CASP2 | 2.02570569 | 3.46E-17 |
| TMEM119 | -7.9670262 | 3.50E-17 |
| MIS18A | 3.2219913 | 3.51E-17 |
| SHC2 | -3.2856752 | 3.55E-17 |
| SSX2IP | 1.26642626 | 3.59E-17 |
| NUP153 | 4.88841237 | 3.76E-17 |
| NCK1 | 1.39317166 | 3.86E-17 |
| HIF1A | 10.4304845 | 3.86E-17 |

| | | |
|---|---|---|
| ZNF367 | 1.37995625 | 3.94E-17 |
| PAIP1 | 4.16651652 | 4.13E-17 |
| CYGB | -2.0014306 | 4.22E-17 |
| CKS1B | 6.30483933 | 4.29E-17 |
| CCNB1 | 8.87396945 | 4.32E-17 |
| RC3H2 | 1.64318342 | 4.36E-17 |
| RPS9 | -49.380157 | 4.39E-17 |
| CUL4B | 2.4751303 | 4.66E-17 |
| KIF5B | 11.2609666 | 4.96E-17 |
| HSD17B12 | 1.26911158 | 5.26E-17 |
| EXOC5 | 1.33691783 | 5.53E-17 |
| OSBPL11 | 1.1929389 | 5.57E-17 |
| ATL2 | 5.29194232 | 5.65E-17 |
| CLEC11A | -8.5042168 | 6.12E-17 |
| EIF2AK2 | 3.89734216 | 6.15E-17 |
| UBXN11 | -2.5794686 | 6.17E-17 |
| MOSPD1 | 1.22337611 | 6.20E-17 |
| STK11 | -1.8166122 | 6.33E-17 |
| PSAT1 | 10.1483305 | 6.35E-17 |
| JUND | -37.78333 | 6.99E-17 |
| C19orf25 | -1.610491 | 7.27E-17 |
| ABI1 | 4.24226559 | 7.75E-17 |
| PUM3 | 6.27422567 | 7.84E-17 |
| PBK | 3.75339434 | 8.01E-17 |
| RILPL2 | -1.261778 | 8.41E-17 |
| YPEL3 | -7.3004629 | 8.83E-17 |
| SFRP2 | -134.77026 | 8.85E-17 |
| CCDC24 | -2.2399257 | 8.94E-17 |
| DNALI1 | -4.9400446 | 8.94E-17 |

| | | |
|---|---|---|
| SLC30A7 | 1.45269252 | 9.79E-17 |
| HMGCS1 | 3.4227727 | 9.88E-17 |
| TGFB1 | -6.2254612 | 1.00E-16 |
| MMADHC | 6.47171073 | 1.03E-16 |
| VMA21 | 2.28700788 | 1.03E-16 |
| HTRA3 | -18.246881 | 1.04E-16 |
| MIS18BP1 | 1.3220575 | 1.07E-16 |
| CTPS1 | 1.70382414 | 1.07E-16 |
| COL1A1 | -433.41088 | 1.09E-16 |
| AEBP1 | -72.713321 | 1.09E-16 |
| SEC23B | 7.08940524 | 1.10E-16 |
| HSPD1 | 21.2011981 | 1.10E-16 |
| UTP20 | 1.50747579 | 1.11E-16 |
| NKD2 | -1.6399443 | 1.13E-16 |
| PDIA4 | 26.2738325 | 1.14E-16 |
| ALDH18A1 | 9.21502023 | 1.15E-16 |
| GPT2 | 4.48408599 | 1.16E-16 |
| RIPK2 | 4.48944546 | 1.17E-16 |
| PANX1 | 2.36046988 | 1.18E-16 |
| ACTL6A | 4.70584629 | 1.18E-16 |
| TM7SF3 | 4.92445052 | 1.20E-16 |
| SPC25 | 1.6545577 | 1.22E-16 |
| CIP2A | 1.32763461 | 1.27E-16 |
| THUMPD3 | 1.55719503 | 1.27E-16 |
| CXorf56 | 1.89791222 | 1.28E-16 |
| ACP1 | 4.15305153 | 1.28E-16 |
| FAM193B | -2.1599487 | 1.30E-16 |
| SSC5D | -2.4094287 | 1.30E-16 |
| C2orf69 | 1.35809664 | 1.33E-16 |

| | | |
|---|---|---|
| PPA1 | 8.27939437 | 1.36E-16 |
| SORBS3 | -4.7511616 | 1.42E-16 |
| TMEM183A | 2.62801645 | 1.44E-16 |
| IDI1 | 6.46795153 | 1.63E-16 |
| RAB11B | -9.6724369 | 1.66E-16 |
| IGBP1 | -6.2143949 | 1.69E-16 |
| NUF2 | 3.53997284 | 1.71E-16 |
| PLEKHH3 | -3.4989725 | 1.78E-16 |
| AURKA | 5.06924478 | 1.81E-16 |
| HJURP | 2.03310709 | 1.94E-16 |
| PTPN12 | 4.58578645 | 2.07E-16 |
| HCFC1R1 | -16.045339 | 2.09E-16 |
| RHOB | -81.653423 | 2.14E-16 |
| ABHD5 | 1.13624071 | 2.18E-16 |
| GLRX3 | 2.05414335 | 2.24E-16 |
| ATG4A | 1.14981114 | 2.26E-16 |
| SCRN2 | -3.8527248 | 2.31E-16 |
| SEH1L | 1.17896813 | 2.33E-16 |
| TBC1D17 | -2.2457305 | 2.42E-16 |
| DNAJC21 | 1.40455457 | 2.58E-16 |
| DLL4 | -1.0629528 | 2.58E-16 |
| TRIM24 | 1.53980663 | 2.58E-16 |
| CDC45 | 1.90775462 | 2.62E-16 |
| CRK | 3.56871105 | 2.69E-16 |
| LIMS2 | -1.0009041 | 2.69E-16 |
| ROCK2 | 1.93090501 | 2.73E-16 |
| DEK | 10.0134025 | 2.74E-16 |
| UBE2T | 7.12904148 | 2.74E-16 |
| OTULIN | 1.11800184 | 2.79E-16 |

| | | |
|---|---|---|
| UBXN1 | -9.0798622 | 2.90E-16 |
| FANCD2 | 1.0377045 | 2.99E-16 |
| CCNE2 | 1.00048263 | 3.04E-16 |
| SLC25A40 | 1.04724669 | 3.10E-16 |
| UGGT1 | 2.40611747 | 3.23E-16 |
| EIF3J | 4.63471334 | 3.23E-16 |
| CENPQ | 1.41216637 | 3.37E-16 |
| LARP4 | 2.53114605 | 3.47E-16 |
| CYTH2 | -2.4144256 | 3.63E-16 |
| TMEM65 | 1.92029526 | 3.76E-16 |
| GLIS2 | -2.6193396 | 3.80E-16 |
| UBXN2A | 1.02182405 | 3.84E-16 |
| CDCA7 | 2.52291313 | 4.02E-16 |
| CIAPIN1 | 3.0102045 | 4.10E-16 |
| MFN1 | 2.66118435 | 4.15E-16 |
| RPS14 | -33.816428 | 4.19E-16 |
| GRSF1 | 3.67101618 | 4.70E-16 |
| RNF138 | 2.0076142 | 4.79E-16 |
| PSMB2 | 6.68064468 | 4.97E-16 |
| RMI1 | 2.04911773 | 5.07E-16 |
| WBP1L | -2.8893648 | 5.16E-16 |
| ACAT2 | 2.87956141 | 5.28E-16 |
| RAB17 | -2.4746576 | 5.66E-16 |
| PLVAP | -17.342943 | 5.94E-16 |
| CDCA2 | 2.11052299 | 6.35E-16 |
| ORC3 | 2.44529146 | 6.36E-16 |
| PRR36 | -3.2507813 | 6.49E-16 |
| SPPL2B | -1.7929109 | 6.61E-16 |
| NT5C2 | 1.02684137 | 6.94E-16 |

| | | |
|---|---|---|
| PITPNA | 3.87548668 | 7.17E-16 |
| DCAF13 | 2.83546672 | 7.41E-16 |
| CACYBP | 5.80649232 | 7.70E-16 |
| CERS5 | -1.190449 | 7.71E-16 |
| MBTPS2 | 1.17898534 | 8.31E-16 |
| PWP1 | 3.21636332 | 8.38E-16 |
| SYPL1 | 13.5883955 | 8.40E-16 |
| PAFAH1B2 | 2.9343634 | 8.79E-16 |
| ZNF219 | -1.3183625 | 8.87E-16 |
| DCTN5 | 1.520141 | 9.07E-16 |
| GYG1 | 1.61218353 | 9.12E-16 |
| HS2ST1 | 1.26048203 | 9.21E-16 |
| SDHC | 10.4048248 | 9.24E-16 |
| SLC39A13 | -2.6494081 | 9.42E-16 |
| NUDT15 | 2.62386731 | 9.66E-16 |
| MAGOHB | 1.46161927 | 1.06E-15 |
| TMEM209 | 1.84082661 | 1.08E-15 |
| AGTPBP1 | 1.50415804 | 1.09E-15 |
| SNRNP70 | -13.098514 | 1.09E-15 |
| NUDT5 | 3.80149049 | 1.11E-15 |
| CHAF1B | 1.73047175 | 1.12E-15 |
| INTS7 | 2.58409191 | 1.14E-15 |
| FSTL3 | -3.6526377 | 1.14E-15 |
| SSBP4 | -3.5150377 | 1.17E-15 |
| PSMA5 | 3.85395983 | 1.20E-15 |
| OSBP | 4.35554211 | 1.24E-15 |
| TBC1D23 | 2.05468314 | 1.24E-15 |
| SUZ12 | 2.81784969 | 1.28E-15 |
| CHCHD5 | -2.2117915 | 1.35E-15 |

| | | |
|---|---|---|
| RNF25 | -2.4815935 | 1.36E-15 |
| GCH1 | 2.35491563 | 1.39E-15 |
| CGAS | 1.08551041 | 1.40E-15 |
| MMP2 | -46.669149 | 1.42E-15 |
| HIBADH | 6.47060291 | 1.50E-15 |
| ATMIN | 2.49229348 | 1.56E-15 |
| DDX6 | 3.31744938 | 1.57E-15 |
| MMGT1 | 2.30388209 | 1.61E-15 |
| PDGFRB | -10.568468 | 1.69E-15 |
| DTL | 2.70917867 | 1.69E-15 |
| PCNA | 27.4742881 | 1.72E-15 |
| MFHAS1 | 1.34283511 | 1.74E-15 |
| LRRC17 | -3.1007392 | 1.79E-15 |
| UBA3 | 2.12312145 | 1.80E-15 |
| SLC5A6 | 11.0659114 | 1.81E-15 |
| U2SURP | 2.09345857 | 1.81E-15 |
| ZYG11B | 1.16929213 | 1.91E-15 |
| BRD7 | 1.54090864 | 1.91E-15 |
| GHDC | -3.1950307 | 1.93E-15 |
| MGMT | -1.7651033 | 1.94E-15 |
| GTSE1 | 1.5935392 | 1.96E-15 |
| ISG20L2 | 3.10138549 | 1.97E-15 |
| PGM1 | 5.35141516 | 1.99E-15 |
| ASF1A | 2.75407276 | 2.00E-15 |
| TMED5 | 2.06028471 | 2.03E-15 |
| GALNT3 | 5.1893554 | 2.12E-15 |
| ABHD17A | -1.3216557 | 2.16E-15 |
| TMEM88 | -1.0435022 | 2.19E-15 |
| COPZ2 | -4.3561129 | 2.25E-15 |

| | | |
|---|---|---|
| RFC3 | 3.21881893 | 2.28E-15 |
| TNFRSF14 | -2.5290778 | 2.44E-15 |
| PRPF4 | 2.45815551 | 2.45E-15 |
| GLB1 | 3.88552385 | 2.45E-15 |
| GNB4 | 2.73660774 | 2.55E-15 |
| MAPK6 | 2.46536416 | 2.56E-15 |
| CNOT9 | 2.39862575 | 2.56E-15 |
| DBR1 | 1.11825454 | 2.58E-15 |
| SIKE1 | 1.6795805 | 2.59E-15 |
| RPL3 | -121.97258 | 2.59E-15 |
| DSN1 | 3.30385084 | 2.61E-15 |
| INTS13 | 3.92657236 | 2.67E-15 |
| DNAJA1 | 21.9831245 | 2.68E-15 |
| UNG | 6.62906683 | 2.69E-15 |
| SMNDC1 | 1.0783957 | 2.71E-15 |
| ACVRL1 | -1.5630439 | 2.91E-15 |
| PMPCB | 1.61832365 | 2.98E-15 |
| ZHX1 | 2.51600336 | 3.00E-15 |
| BTBD3 | 2.35681243 | 3.07E-15 |
| AP1G1 | 2.86489203 | 3.12E-15 |
| TTLL4 | 2.36599557 | 3.30E-15 |
| PRELID3B | 11.2069875 | 3.40E-15 |
| UPF2 | 3.04173678 | 3.42E-15 |
| PTDSS1 | 5.90839849 | 3.42E-15 |
| SEPHS1 | 6.22933715 | 3.48E-15 |
| LTBP2 | -8.2917357 | 3.62E-15 |
| NDUFAF4 | 1.77395881 | 3.80E-15 |
| SET | 16.8094123 | 3.83E-15 |
| SENP5 | 1.4920787 | 3.86E-15 |

| | | |
|---|---|---|
| COQ2 | 1.05826536 | 3.88E-15 |
| PKIG | -3.9688156 | 3.93E-15 |
| PNPLA2 | -7.142676 | 4.06E-15 |
| ITCH | 1.49542083 | 4.17E-15 |
| RFC2 | 4.59879739 | 4.19E-15 |
| EML3 | -1.82742 | 4.21E-15 |
| COQ3 | 1.85263479 | 4.24E-15 |
| RNF168 | 1.49511366 | 4.28E-15 |
| FAM91A1 | 4.08085545 | 4.28E-15 |
| HENMT1 | 3.32903131 | 4.31E-15 |
| RANBP9 | 4.27984873 | 4.37E-15 |
| PAICS | 7.57665086 | 4.56E-15 |
| BTBD2 | -3.0831336 | 4.56E-15 |
| TLE3 | -3.1877685 | 4.89E-15 |
| TIMM17A | 5.26620551 | 5.09E-15 |
| RAD18 | 1.00814809 | 5.09E-15 |
| GNL3L | 1.14350591 | 5.16E-15 |
| CCNF | 1.30629135 | 5.35E-15 |
| SPARCL1 | -33.507743 | 5.38E-15 |
| RAN | 11.2811136 | 5.50E-15 |
| STK35 | 2.17092691 | 5.67E-15 |
| CLP1 | 1.40004488 | 6.04E-15 |
| KIF1B | 1.2343186 | 6.35E-15 |
| TM9SF2 | 13.8597388 | 6.41E-15 |
| RPL29 | -73.020666 | 6.58E-15 |
| STRAP | 20.5567182 | 6.66E-15 |
| ANP32E | 12.0947308 | 6.93E-15 |
| KDELR2 | 18.9861541 | 6.96E-15 |
| MLPH | -23.259301 | 7.29E-15 |

| | | |
|---|---|---|
| PGAM1 | 2.90036414 | 7.45E-15 |
| G3BP1 | 2.01304022 | 7.45E-15 |
| RAMP2 | -10.143114 | 7.76E-15 |
| STX3 | 1.72652216 | 8.44E-15 |
| MYBL2 | 14.3806854 | 9.24E-15 |
| PLAAT1 | 1.84863051 | 9.32E-15 |
| TBX3 | -10.557626 | 9.39E-15 |
| CDYL | 1.88675604 | 9.49E-15 |
| DLG1 | 1.14723218 | 9.75E-15 |
| RAB9A | 3.68679925 | 9.89E-15 |
| C1GALT1C1 | 3.86994157 | 1.01E-14 |
| ATP5MC2 | -15.422657 | 1.01E-14 |
| CRY2 | -2.0568708 | 1.05E-14 |
| AARS1 | 4.83866434 | 1.06E-14 |
| SLC25A33 | 1.20055157 | 1.12E-14 |
| APBB3 | -1.2371228 | 1.15E-14 |
| PHLDA3 | -5.7976718 | 1.22E-14 |
| UBXN6 | -4.5854771 | 1.24E-14 |
| KDM1B | 2.41301876 | 1.25E-14 |
| SERPINF1 | -23.064493 | 1.25E-14 |
| COMMD2 | 1.86698912 | 1.27E-14 |
| CCDC12 | -2.1770901 | 1.30E-14 |
| COPB1 | 7.6025747 | 1.35E-14 |
| PGM3 | 1.68857542 | 1.36E-14 |
| PSMG1 | 4.75015891 | 1.36E-14 |
| MYO10 | 2.78062135 | 1.37E-14 |
| RBL1 | 1.26998591 | 1.37E-14 |
| FAM3C2P | 1.04898719 | 1.38E-14 |
| ARMC10 | 1.79434825 | 1.40E-14 |

| | | |
|---|---|---|
| SBNO1 | 1.58949409 | 1.49E-14 |
| G3BP2 | 3.02924382 | 1.52E-14 |
| NUP50 | 1.70120033 | 1.55E-14 |
| CILK1 | 2.0487404 | 1.61E-14 |
| GTF2H3 | 1.47891143 | 1.67E-14 |
| CMTR2 | 1.0139689 | 1.68E-14 |
| DNAJB11 | 1.59988949 | 1.73E-14 |
| STAMBP | 1.10116072 | 1.76E-14 |
| NXT2 | 2.2525452 | 1.80E-14 |
| ACAP2 | 1.49692934 | 1.87E-14 |
| POP1 | 1.27300266 | 1.91E-14 |
| ADSS2 | 5.71975155 | 2.02E-14 |
| RANBP2 | 2.75759808 | 2.05E-14 |
| B4GALT5 | 6.04942019 | 2.09E-14 |
| PHTF2 | 1.09661382 | 2.15E-14 |
| SSB | 2.92978113 | 2.22E-14 |
| ATAD2 | 6.06143255 | 2.33E-14 |
| GPI | 8.12160063 | 2.47E-14 |
| WAC | 2.65286856 | 2.49E-14 |
| DROSHA | 1.83226622 | 2.49E-14 |
| PAFAH1B1 | 1.70068878 | 2.59E-14 |
| NOC3L | 1.1018169 | 2.59E-14 |
| YJU2 | -3.0505593 | 2.71E-14 |
| EFR3A | 6.24366427 | 2.76E-14 |
| HBS1L | 1.92354368 | 2.90E-14 |
| GCLM | 1.70013185 | 2.93E-14 |
| GRHL1 | 2.32539426 | 2.95E-14 |
| TTC13 | 1.63335662 | 2.98E-14 |
| GOLGA4 | 4.21582894 | 3.08E-14 |

| | | |
|---|---|---|
| ZNF800 | 1.15763365 | 3.17E-14 |
| CCDC71 | -2.6385697 | 3.17E-14 |
| BTF3 | -20.194944 | 3.44E-14 |
| OAT | 16.6117104 | 3.55E-14 |
| MKLN1 | 1.14051943 | 3.59E-14 |
| CLEC3B | -1.9773657 | 3.63E-14 |
| IGFBP6 | -5.3113101 | 3.66E-14 |
| MRPL13 | 4.07555695 | 4.11E-14 |
| DHX15 | 3.47619361 | 4.19E-14 |
| ELOA | 1.75450735 | 4.24E-14 |
| TIMELESS | 2.7408483 | 4.24E-14 |
| CCT5 | 10.3833293 | 4.27E-14 |
| CCDC159 | -1.5669343 | 4.31E-14 |
| ICE1 | 1.7656245 | 4.31E-14 |
| RNF114 | 6.89983429 | 4.72E-14 |
| EIF2S1 | 3.13470457 | 4.93E-14 |
| SAFB2 | -1.5157249 | 4.98E-14 |
| HSP90B1 | 35.8663438 | 5.06E-14 |
| SNX4 | 3.03507325 | 5.21E-14 |
| PCMT1 | 5.37388704 | 5.38E-14 |
| C9orf40 | 1.14476866 | 5.54E-14 |
| CCDC106 | -2.8323904 | 5.83E-14 |
| PHPT1 | -10.477356 | 6.03E-14 |
| THNSL1 | 1.12496188 | 6.13E-14 |
| ZBED4 | 1.50119167 | 6.14E-14 |
| MZT2A | -4.5492257 | 6.18E-14 |
| CALU | 13.4830623 | 6.27E-14 |
| JUNB | -42.525401 | 6.48E-14 |
| SMARCA5 | 3.38097285 | 7.19E-14 |

| | | |
|---|---|---|
| RNF6 | 2.44920035 | 7.19E-14 |
| NEMP1 | 1.98043355 | 7.47E-14 |
| AIMP2 | 1.44369615 | 7.53E-14 |
| GRPEL2 | 1.05594361 | 7.60E-14 |
| NCAPD2 | 7.63370191 | 7.67E-14 |
| PGRMC1 | 21.3579504 | 7.86E-14 |
| MTDH | 9.13072178 | 7.89E-14 |
| UAP1 | 9.5371088 | 7.97E-14 |
| PCED1A | -4.0446314 | 8.16E-14 |
| GTF2F1 | -5.770347 | 8.20E-14 |
| NIFK | 2.18427667 | 8.42E-14 |
| GTF3C4 | 2.1210815 | 8.47E-14 |
| FBXO28 | 3.10016564 | 8.56E-14 |
| LRR1 | 1.13537084 | 8.56E-14 |
| LENG1 | -2.167013 | 8.62E-14 |
| SSBP1 | 2.94027382 | 9.16E-14 |
| MCM3 | 12.0450781 | 9.49E-14 |
| CPLX1 | -2.2584448 | 9.50E-14 |
| OXCT1 | 3.79407816 | 9.59E-14 |
| CHEK2 | 1.00048031 | 1.01E-13 |
| REPS1 | 1.35905498 | 1.01E-13 |
| AREL1 | 1.12239888 | 1.05E-13 |
| DEGS2 | -11.884442 | 1.05E-13 |
| MPZL3 | 1.60683761 | 1.06E-13 |
| SLC35F2 | 3.0926659 | 1.10E-13 |
| RIOK1 | 3.38017305 | 1.11E-13 |
| RIF1 | 1.17065573 | 1.12E-13 |
| FMNL2 | 2.14698394 | 1.12E-13 |
| TGS1 | 2.05799127 | 1.17E-13 |

| | | |
|---|---|---|
| CDCA5 | 4.00403786 | 1.20E-13 |
| CLASRP | -2.0720791 | 1.20E-13 |
| RBBP5 | 2.24067629 | 1.20E-13 |
| GOLPH3 | 10.7189518 | 1.38E-13 |
| PDGFRL | -4.545964 | 1.40E-13 |
| PTGES3 | 18.1986928 | 1.40E-13 |
| CCDC61 | -1.2876451 | 1.40E-13 |
| XPO6 | 3.07217289 | 1.46E-13 |
| METAP2 | 3.28462122 | 1.46E-13 |
| MTERF3 | 4.95261139 | 1.50E-13 |
| EIF2S3 | 18.3386179 | 1.50E-13 |
| CISD1 | 1.70312584 | 1.52E-13 |
| AMD1 | 3.9028032 | 1.55E-13 |
| CDC23 | 1.50756874 | 1.55E-13 |
| BMS1 | 1.82336415 | 1.57E-13 |
| UBXN4 | 6.16452646 | 1.57E-13 |
| PPM1M | -1.3651216 | 1.59E-13 |
| PSME3 | 4.53908392 | 1.61E-13 |
| FBXO11 | 1.03839888 | 1.66E-13 |
| SURF4 | 12.8366975 | 1.72E-13 |
| STK38 | 5.67127393 | 1.77E-13 |
| KARS1 | 7.26687126 | 1.83E-13 |
| SPRING1 | 2.37664792 | 1.89E-13 |
| ZNF414 | -1.1258571 | 1.90E-13 |
| LMNB1 | 6.63736757 | 1.90E-13 |
| SAE1 | 5.68392545 | 1.97E-13 |
| H1-10 | -16.354907 | 1.97E-13 |
| CD34 | -2.5384181 | 1.99E-13 |
| ADGRA2 | -1.7277265 | 2.01E-13 |

| | | |
|---|---|---|
| EIF1AX | 4.34498529 | 2.01E-13 |
| EIF2AK3 | 1.47992796 | 2.09E-13 |
| TMEM165 | 2.79320824 | 2.10E-13 |
| M6PR | 4.36377004 | 2.10E-13 |
| CHORDC1 | 1.06028668 | 2.11E-13 |
| ZBTB11 | 1.00455654 | 2.13E-13 |
| RLIM | 1.59575736 | 2.23E-13 |
| NUP133 | 3.00514299 | 2.28E-13 |
| CEBPG | 4.44861315 | 2.31E-13 |
| MEGF6 | -1.7446812 | 2.32E-13 |
| SH2D3C | -1.44867 | 2.33E-13 |
| PLCD3 | -2.2275959 | 2.33E-13 |
| DAP3 | 6.9889658 | 2.46E-13 |
| RLF | 1.19733291 | 2.48E-13 |
| SSRP1 | 7.11518562 | 2.50E-13 |
| SCAF4 | 1.41494062 | 2.69E-13 |
| POLM | -1.1385263 | 2.72E-13 |
| FBH1 | 1.90752705 | 2.76E-13 |
| CDC27 | 2.43679887 | 2.85E-13 |
| STT3B | 9.78751898 | 2.85E-13 |
| TMEM167A | 3.99612332 | 2.88E-13 |
| MFSD1 | 3.23062821 | 2.91E-13 |
| NUP98 | 2.33466807 | 2.93E-13 |
| IREB2 | 1.38489561 | 3.07E-13 |
| WDR76 | 1.28517027 | 3.07E-13 |
| DUSP1 | -45.960845 | 3.07E-13 |
| UBALD1 | -2.0565991 | 3.12E-13 |
| MEAK7 | 1.22552467 | 3.13E-13 |
| FUZ | -1.529381 | 3.21E-13 |

| | | |
|---|---|---|
| NINJ1 | -12.025252 | 3.22E-13 |
| C11orf68 | -5.3830476 | 3.25E-13 |
| INAVA | 1.79977088 | 3.28E-13 |
| NAA35 | 1.14309429 | 3.39E-13 |
| IPO8 | 1.9375102 | 3.43E-13 |
| FOXN2 | 1.11275603 | 3.46E-13 |
| CCNY | 3.06839754 | 3.49E-13 |
| PLD4 | -1.4517974 | 3.49E-13 |
| CBX3 | 11.8116216 | 3.49E-13 |
| LRRC8D | 3.13023668 | 3.57E-13 |
| DIAPH1 | 4.15353001 | 3.57E-13 |
| CST3 | -42.915728 | 3.74E-13 |
| IFIH1 | 5.7397799 | 3.76E-13 |
| SLC25A44 | 1.32033085 | 3.78E-13 |
| LMOD1 | -2.6836732 | 3.82E-13 |
| XPO7 | 2.78950681 | 3.91E-13 |
| ETFA | 4.26226323 | 3.98E-13 |
| MTFR1 | 3.45997717 | 3.99E-13 |
| PCCB | 1.64409734 | 3.99E-13 |
| LYRM2 | 1.03896994 | 4.06E-13 |
| ISLR | -24.372455 | 4.07E-13 |
| ZNF205 | -2.1326477 | 4.15E-13 |
| MYL9 | -33.770701 | 4.19E-13 |
| PITPNB | 2.74589763 | 4.22E-13 |
| CLK1 | -4.1986446 | 4.26E-13 |
| MZF1 | -1.0366759 | 4.27E-13 |
| HMGA1 | 27.8833538 | 4.28E-13 |
| GPR157 | 1.49936903 | 4.37E-13 |
| CHMP2B | 1.39477411 | 4.37E-13 |

| | | |
|---|---|---|
| NUP54 | 1.81495618 | 4.41E-13 |
| COPS3 | 4.32704687 | 4.41E-13 |
| CCDC9 | -1.9038608 | 4.56E-13 |
| LUC7L | -2.1448901 | 4.59E-13 |
| SLC4A1AP | 1.05817105 | 4.62E-13 |
| PPP2R3A | 1.37006769 | 4.62E-13 |
| IFI27L2 | -4.0198014 | 4.66E-13 |
| AMOT | 2.10877885 | 4.75E-13 |
| SSR3 | 7.77441731 | 4.75E-13 |
| ODC1 | 9.61734391 | 4.76E-13 |
| MCM7 | 10.0322465 | 4.76E-13 |
| GSN | -13.088433 | 4.82E-13 |
| CNOT10 | 1.14573272 | 4.83E-13 |
| OTUD6B | 1.55103906 | 5.00E-13 |
| MTMR1 | 1.37005152 | 5.06E-13 |
| CMAS | 7.00322126 | 5.19E-13 |
| PRDX4 | 16.4212891 | 5.26E-13 |
| EIF4G2 | 22.2101411 | 5.44E-13 |
| NRBF2 | 2.82872599 | 5.52E-13 |
| SLC7A5 | 24.2177898 | 5.69E-13 |
| RHOJ | -1.1691944 | 5.70E-13 |
| RABIF | 1.73340696 | 5.98E-13 |
| SPG21 | 3.83571006 | 6.20E-13 |
| CDC20 | 11.684999 | 6.47E-13 |
| PLCB4 | 1.52562718 | 6.47E-13 |
| NT5C | -2.7094231 | 6.51E-13 |
| PGM2 | 2.29094477 | 6.60E-13 |
| PPP2R1B | 2.09553984 | 6.64E-13 |
| URB1 | 1.0848345 | 6.79E-13 |

| | | |
|---|---|---|
| GSPT1 | 5.89159782 | 6.81E-13 |
| CPSF2 | 1.07030716 | 7.21E-13 |
| DARS1 | 2.95991729 | 7.27E-13 |
| UBQLN4 | 5.22925277 | 7.29E-13 |
| CMTM4 | 3.0187885 | 7.66E-13 |
| HTATSF1 | 7.02379689 | 7.66E-13 |
| TFG | 2.11190926 | 7.66E-13 |
| ARMC1 | 4.47377327 | 7.85E-13 |
| PIK3IP1 | -3.3575159 | 8.17E-13 |
| SRPK2 | 2.86599312 | 8.46E-13 |
| PLXNB1 | -3.4153331 | 8.52E-13 |
| SYNDIG1 | -1.7850762 | 8.62E-13 |
| SF3A3 | 4.85710767 | 8.78E-13 |
| CORO1C | 4.21658211 | 8.78E-13 |
| COL16A1 | -6.4278033 | 8.89E-13 |
| RNASEH1 | 1.01673994 | 9.00E-13 |
| EHF | 16.8031291 | 9.46E-13 |
| HSF2 | 1.58954182 | 9.59E-13 |
| NARS1 | 8.60458407 | 9.60E-13 |
| DIPK2B | -1.1859567 | 9.72E-13 |
| DMPK | -1.3259448 | 9.93E-13 |
| ECPAS | 3.53071991 | 1.01E-12 |
| DPP7 | -14.501123 | 1.02E-12 |
| HYAL2 | -2.3612647 | 1.03E-12 |
| FAAP20 | -1.1247216 | 1.03E-12 |
| CERCAM | -4.8883617 | 1.04E-12 |
| PEX14 | -1.3749169 | 1.04E-12 |
| QSER1 | 2.10212578 | 1.08E-12 |
| MRPS35 | 10.0057753 | 1.09E-12 |

| | | |
|---|---|---|
| RAP2A | 3.29879009 | 1.12E-12 |
| USB1 | 1.34619395 | 1.13E-12 |
| UBE2V2 | 3.17155924 | 1.14E-12 |
| RPLP1 | -117.24513 | 1.22E-12 |
| RARS1 | 3.4086533 | 1.23E-12 |
| TUBGCP6 | -1.5319513 | 1.34E-12 |
| ESYT2 | 5.33439264 | 1.34E-12 |
| STAT1 | 18.4212158 | 1.36E-12 |
| SINHCAF | 3.68069106 | 1.39E-12 |
| OCRL | 2.44047712 | 1.39E-12 |
| LMCD1 | -2.5152683 | 1.47E-12 |
| UTP6 | 1.66303211 | 1.51E-12 |
| MMRN2 | -2.6062221 | 1.51E-12 |
| YTHDF3 | 4.13690239 | 1.54E-12 |
| TCF19 | 4.52878659 | 1.56E-12 |
| COL8A2 | -7.1391861 | 1.57E-12 |
| OLFML2A | -2.2201329 | 1.58E-12 |
| CTSC | 2.08162917 | 1.59E-12 |
| DSC2 | 4.90486979 | 1.69E-12 |
| RNF26 | 1.94734599 | 1.70E-12 |
| CANX | 28.4661848 | 1.70E-12 |
| SNX6 | 3.32539918 | 1.71E-12 |
| HMGCR | 2.32361027 | 1.71E-12 |
| PTOV1 | -1.3521966 | 1.74E-12 |
| LACTB2 | 1.73489922 | 1.75E-12 |
| STAU1 | 10.896548 | 1.76E-12 |
| GAMT | -6.9545264 | 1.78E-12 |
| NPC1 | 1.18574571 | 1.87E-12 |
| PCYT1A | 1.57234476 | 1.91E-12 |

| | | |
|---|---|---|
| CLIP3 | -1.5666747 | 1.92E-12 |
| YWHAZ | 40.6335487 | 1.94E-12 |
| TNPO1 | 2.23859915 | 1.96E-12 |
| SLC25A43 | 1.98676064 | 1.97E-12 |
| PHYHD1 | -3.4260619 | 1.97E-12 |
| HPRT1 | 6.48998244 | 1.97E-12 |
| LRP1 | -6.7915567 | 2.00E-12 |
| RHOC | -10.565761 | 2.03E-12 |
| SLC35B3 | 1.34874359 | 2.05E-12 |
| ZNF318 | 1.12448034 | 2.12E-12 |
| CYB5B | 1.49757466 | 2.16E-12 |
| KLHDC10 | 2.79778476 | 2.17E-12 |
| CDKN3 | 2.29343867 | 2.17E-12 |
| MPZL2 | 3.06369165 | 2.18E-12 |
| UBR3 | 1.3857034 | 2.18E-12 |
| BPNT1 | 3.61284996 | 2.22E-12 |
| SOX11 | 2.17121897 | 2.23E-12 |
| COL18A1 | -6.927227 | 2.29E-12 |
| DNAJC11 | 1.0139589 | 2.30E-12 |
| BCLAF1 | 2.66491341 | 2.30E-12 |
| ZCCHC24 | -2.5593946 | 2.36E-12 |
| GATA3 | -71.189684 | 2.38E-12 |
| MAP2K1 | 2.21439283 | 2.50E-12 |
| WASHC4 | 1.58951936 | 2.58E-12 |
| ADNP2 | 1.39127303 | 2.69E-12 |
| PDS5A | 2.52622068 | 2.74E-12 |
| CCAR1 | 2.16398373 | 2.74E-12 |
| LTV1 | 2.31299868 | 2.79E-12 |
| DNAJC13 | 1.62126688 | 2.90E-12 |

| | | |
|---|---|---|
| FZR1 | -1.6867456 | 2.97E-12 |
| PRPF8 | 5.80645259 | 3.01E-12 |
| TDRKH | 1.80046357 | 3.03E-12 |
| CALR | 75.0564405 | 3.05E-12 |
| ZDHHC13 | 1.02899765 | 3.15E-12 |
| PRPS2 | 6.03616213 | 3.18E-12 |
| PHKA1 | 1.24119341 | 3.18E-12 |
| ADAR | 15.3158303 | 3.22E-12 |
| ADAT1 | 1.34285126 | 3.27E-12 |
| RARRES2 | -12.982829 | 3.41E-12 |
| LDHA | 28.7491544 | 3.55E-12 |
| RIOX2 | 1.02816307 | 3.59E-12 |
| GPN3 | 1.43324403 | 3.60E-12 |
| CDC73 | 1.40708301 | 3.62E-12 |
| DPT | -4.4187983 | 3.68E-12 |
| NUP88 | 1.17396588 | 3.68E-12 |
| CLPX | 1.43464729 | 3.75E-12 |
| CENPBD1 | 1.18287799 | 3.77E-12 |
| TGFB3 | -5.7258429 | 3.77E-12 |
| UTP11 | 2.21721382 | 3.81E-12 |
| CCSER2 | 1.51851986 | 3.88E-12 |
| PGPEP1 | -1.3892943 | 3.90E-12 |
| ENG | -7.4759644 | 3.91E-12 |
| ZWINT | 7.06828818 | 3.93E-12 |
| PRPF4B | 2.08059004 | 4.09E-12 |
| PARP1 | 3.60231472 | 4.09E-12 |
| PROCR | -1.9491137 | 4.13E-12 |
| ZBTB17 | -1.215174 | 4.13E-12 |
| IFT43 | -1.2861286 | 4.15E-12 |

| | | |
|---|---|---|
| TES | 4.94084827 | 4.16E-12 |
| FLYWCH1 | -1.0075597 | 4.16E-12 |
| OGFOD1 | 1.14305639 | 4.19E-12 |
| MCFD2 | 3.33926127 | 4.20E-12 |
| INO80E | -1.8902255 | 4.22E-12 |
| RMND5A | 2.64182369 | 4.25E-12 |
| YEATS2 | 1.33400747 | 4.31E-12 |
| ANKRD27 | 1.83876475 | 4.33E-12 |
| NXNL2 | -1.2860252 | 4.40E-12 |
| ZMPSTE24 | 4.91939842 | 4.44E-12 |
| CDK2 | 1.72184815 | 4.48E-12 |
| UBAP2 | 1.14220505 | 4.54E-12 |
| JAM2 | -1.0665842 | 4.54E-12 |
| INIP | 1.65297038 | 4.60E-12 |
| OSGIN2 | 3.63459061 | 4.62E-12 |
| SLC7A1 | 3.02885557 | 4.64E-12 |
| SUGP1 | -1.1201095 | 4.65E-12 |
| F11R | 7.33506741 | 4.75E-12 |
| XRN1 | 1.10941011 | 4.80E-12 |
| CCDC6 | 5.61921375 | 4.88E-12 |
| USP24 | 1.86989133 | 5.11E-12 |
| DYNC1LI2 | 2.52606472 | 5.18E-12 |
| HADHB | 4.57574284 | 5.19E-12 |
| ATP5PB | 6.04482448 | 5.42E-12 |
| SEC23IP | 2.07458359 | 5.45E-12 |
| TAB2 | 4.26823121 | 5.53E-12 |
| CILP2 | -2.4516256 | 5.62E-12 |
| YARS1 | 2.32631434 | 5.70E-12 |
| RAB3GAP2 | 1.64392329 | 5.74E-12 |

| | | |
|---|---|---|
| MIDN | -6.6887569 | 5.93E-12 |
| NUB1 | 2.47704569 | 6.06E-12 |
| FLOT1 | -14.618908 | 6.10E-12 |
| DTX4 | 2.23403624 | 6.19E-12 |
| LZTS2 | -2.6731814 | 6.28E-12 |
| MDH2 | 14.4359797 | 6.31E-12 |
| HLTF | 2.16938209 | 6.47E-12 |
| APOOL | 1.09556805 | 6.69E-12 |
| MRPL44 | 2.20527829 | 6.72E-12 |
| RPL34 | -31.34251 | 6.78E-12 |
| BHLHE40 | -38.861973 | 7.02E-12 |
| SMC3 | 3.31761766 | 7.10E-12 |
| GAS7 | -1.6801473 | 7.24E-12 |
| CASP8AP2 | 1.03136909 | 7.32E-12 |
| CD248 | -9.1555515 | 7.38E-12 |
| UBTD1 | -2.8590964 | 7.40E-12 |
| PVR | 1.71871751 | 7.54E-12 |
| THOC2 | 1.36607947 | 7.58E-12 |
| FADS2 | 13.748886 | 7.76E-12 |
| TADA1 | 1.40490785 | 7.76E-12 |
| SMCHD1 | 1.65059674 | 7.83E-12 |
| RPL10 | -46.401274 | 7.84E-12 |
| GRP | -3.9323942 | 7.99E-12 |
| IP6K2 | -2.3821529 | 8.04E-12 |
| UTP25 | 1.02926724 | 8.16E-12 |
| ANGPTL2 | -7.4583527 | 8.49E-12 |
| PSMD5 | 3.05010741 | 8.74E-12 |
| SLC11A2 | 1.87941654 | 8.84E-12 |
| PIN1 | -2.0834234 | 8.84E-12 |

| | | |
|---|---|---|
| ZNF644 | 1.65332912 | 9.08E-12 |
| TMEM70 | 2.76454294 | 9.21E-12 |
| EPB41L4B | 1.33739161 | 9.21E-12 |
| RHNO1 | 3.58457458 | 9.29E-12 |
| ARPC5 | 3.62924298 | 9.36E-12 |
| NFE2L3 | 2.56456882 | 9.45E-12 |
| NUP43 | 2.14738278 | 9.54E-12 |
| TBL2 | 1.61276325 | 9.60E-12 |
| TUBA1B | 7.15950926 | 9.60E-12 |
| CCDC130 | -2.3533303 | 9.69E-12 |
| ABRACL | 16.4623112 | 9.90E-12 |
| MVB12A | -2.6990102 | 1.01E-11 |
| RBM17 | 3.33833838 | 1.01E-11 |
| NSF | 3.29903749 | 1.02E-11 |
| MRE11 | 1.04367447 | 1.02E-11 |
| CNKSR1 | -1.9000169 | 1.02E-11 |
| GORASP2 | 4.12321226 | 1.03E-11 |
| DNAJC3 | 5.25746279 | 1.06E-11 |
| AURKB | 3.22417435 | 1.06E-11 |
| C1QBP | 8.55370806 | 1.07E-11 |
| DMAP1 | -1.4564395 | 1.12E-11 |
| INKA1 | -1.0667075 | 1.16E-11 |
| SYNPO | -2.2557996 | 1.20E-11 |
| CLEC7A | 2.05457274 | 1.20E-11 |
| RCN2 | 1.63428042 | 1.20E-11 |
| TPR | 3.41077002 | 1.22E-11 |
| DPH2 | 2.11014682 | 1.22E-11 |
| TMEM129 | -3.044117 | 1.24E-11 |
| GLT8D2 | -2.2517493 | 1.25E-11 |

| | | |
|---|---|---|
| ATP11B | 2.11260698 | 1.29E-11 |
| SNRNP200 | 5.67405425 | 1.29E-11 |
| AGO2 | 1.85669567 | 1.31E-11 |
| SON | 3.94551242 | 1.31E-11 |
| LGALS1 | -118.44637 | 1.31E-11 |
| THY1 | -6.7106127 | 1.35E-11 |
| HEXD | -1.6111493 | 1.36E-11 |
| RPL9 | -21.406006 | 1.38E-11 |
| CHST14 | -2.1998389 | 1.43E-11 |
| COL6A1 | -79.01338 | 1.43E-11 |
| NOSIP | -2.9233637 | 1.47E-11 |
| P3H3 | -3.7696524 | 1.47E-11 |
| RAI2 | -5.4340917 | 1.47E-11 |
| SPARC | -234.60589 | 1.54E-11 |
| AVL9 | 1.31582989 | 1.54E-11 |
| KDM4B | -4.0586038 | 1.56E-11 |
| EMILIN1 | -12.569109 | 1.61E-11 |
| POGK | 3.36425798 | 1.61E-11 |
| IRF2BP1 | -3.3704438 | 1.64E-11 |
| P4HTM | -2.177202 | 1.70E-11 |
| C3orf38 | 1.05791052 | 1.72E-11 |
| SIRT6 | -1.5245864 | 1.72E-11 |
| PGD | 11.2457064 | 1.72E-11 |
| ATP6V0A4 | 1.62981571 | 1.73E-11 |
| EBF4 | -3.6609144 | 1.74E-11 |
| ANKRD17 | 2.87247213 | 1.74E-11 |
| HSPH1 | 7.20207082 | 1.85E-11 |
| OLFML1 | -1.2018804 | 1.88E-11 |
| NFATC4 | -3.4112693 | 1.89E-11 |

| | | |
|---|---|---|
| WDR77 | 1.42087991 | 1.93E-11 |
| UFL1 | 2.60244179 | 1.97E-11 |
| SDCBP | 9.69142278 | 1.98E-11 |
| PURB | 2.19163328 | 2.01E-11 |
| FDPS | 2.82734141 | 2.11E-11 |
| IFNAR1 | 2.03885693 | 2.15E-11 |
| NUP58 | 1.40853659 | 2.17E-11 |
| SDE2 | 1.98260418 | 2.19E-11 |
| BROX | 4.35337801 | 2.27E-11 |
| HTATIP2 | 4.86681776 | 2.34E-11 |
| JUN | -28.017742 | 2.36E-11 |
| PSPH | 2.57372976 | 2.37E-11 |
| XRN2 | 6.77401959 | 2.47E-11 |
| TMEM259 | -3.5585971 | 2.48E-11 |
| FAM160A2 | -1.4496298 | 2.50E-11 |
| UBTF | -2.9806447 | 2.52E-11 |
| DKK3 | -1.8442532 | 2.52E-11 |
| ALKBH7 | -12.01555 | 2.52E-11 |
| TSPYL2 | -1.6851806 | 2.54E-11 |
| CCDC77 | 1.16368983 | 2.60E-11 |
| GNPTG | -3.3090755 | 2.66E-11 |
| EIF3G | -6.3394726 | 2.71E-11 |
| E2F1 | 3.47965689 | 2.71E-11 |
| CXXC1 | -2.1431327 | 2.89E-11 |
| RBM5 | -1.5142038 | 3.04E-11 |
| PTK2 | 2.68698113 | 3.12E-11 |
| SMG7 | 2.93601198 | 3.18E-11 |
| RFC5 | 1.20685249 | 3.23E-11 |
| BCL6B | -1.0740886 | 3.29E-11 |

| | | |
|---|---|---|
| CAMSAP2 | 2.15464433 | 3.35E-11 |
| PDXDC1 | 5.71200259 | 3.36E-11 |
| ZNF444 | -1.4751805 | 3.37E-11 |
| TAP1 | 12.7962448 | 3.40E-11 |
| RECQL | 1.87434369 | 3.41E-11 |
| TPI1 | 44.7741096 | 3.47E-11 |
| SGSM2 | -1.7676949 | 3.50E-11 |
| SAP130 | 1.4287735 | 3.51E-11 |
| GALNT16 | -1.2312006 | 3.52E-11 |
| ABITRAM | 1.66265798 | 3.54E-11 |
| HSP90AB1 | 145.944802 | 3.58E-11 |
| TRIP12 | 2.92940643 | 3.68E-11 |
| MSANTD3 | 1.14209779 | 3.69E-11 |
| TRA2B | 1.21829367 | 3.70E-11 |
| RO60 | 1.76492926 | 3.81E-11 |
| AOC3 | -4.1963536 | 3.93E-11 |
| RBPMS | -2.1465642 | 3.95E-11 |
| TATDN1 | 1.37327494 | 3.97E-11 |
| SLBP | 5.72678651 | 3.98E-11 |
| NEPRO | 1.02744361 | 4.04E-11 |
| SNRPG | 5.42611031 | 4.07E-11 |
| NSMAF | 1.40168291 | 4.08E-11 |
| COPS9 | -8.926854 | 4.15E-11 |
| PARP12 | 3.06660218 | 4.19E-11 |
| ADD1 | -2.4043139 | 4.25E-11 |
| FAM220A | 1.45972201 | 4.35E-11 |
| TRAP1 | 2.06189871 | 4.42E-11 |
| FEN1 | 5.82044399 | 4.56E-11 |
| H2AZ1 | 16.7158831 | 4.59E-11 |

| | | |
|---|---|---|
| IRAK1 | 12.7766369 | 4.73E-11 |
| MTMR12 | 3.21410046 | 4.75E-11 |
| MIS12 | 1.17224483 | 4.86E-11 |
| IBTK | 1.84824025 | 4.88E-11 |
| RBBP9 | 2.43653687 | 4.93E-11 |
| RRAGD | 1.5303804 | 4.96E-11 |
| MRPL37 | 8.1401373 | 4.96E-11 |
| PRRC2C | 5.00569268 | 4.96E-11 |
| TMPRSS13 | 1.93135452 | 4.96E-11 |
| MRPL35 | 1.49722461 | 5.51E-11 |
| BTG2 | -59.979414 | 5.60E-11 |
| PDLIM4 | -3.0443273 | 5.62E-11 |
| SLC12A4 | -1.0165722 | 5.96E-11 |
| ESCO1 | 1.0764369 | 5.97E-11 |
| FOS | -59.1162 | 6.12E-11 |
| DNTTIP2 | 1.6787578 | 6.15E-11 |
| LPIN1 | 1.66208182 | 6.17E-11 |
| RPL13A | -127.17593 | 6.30E-11 |
| RPIA | 2.74040882 | 6.45E-11 |
| EMC1 | 1.31079597 | 6.49E-11 |
| MYD88 | 3.26198773 | 6.54E-11 |
| TMEM175 | -1.6077703 | 6.64E-11 |
| NOL11 | 4.56638991 | 6.70E-11 |
| SLC38A10 | -3.5344481 | 6.89E-11 |
| NOMO1 | 2.29131207 | 7.12E-11 |
| DNAJC4 | -2.2810644 | 7.20E-11 |
| PFKP | 3.56070436 | 7.33E-11 |
| ATP11A | 1.18699658 | 7.39E-11 |
| JRKL | 1.13090293 | 7.42E-11 |

| | | |
|---|---|---|
| CCN1 | -27.652808 | 7.42E-11 |
| VCP | 7.57664713 | 7.68E-11 |
| SF3A2 | -4.9148543 | 8.15E-11 |
| IWS1 | 1.84174018 | 8.37E-11 |
| PIMREG | 1.34536111 | 8.38E-11 |
| UBE2C | 16.1417391 | 8.42E-11 |
| CSTF3 | 1.38440933 | 8.54E-11 |
| PRKD3 | 1.48231292 | 8.57E-11 |
| SGF29 | -1.8245705 | 8.92E-11 |
| EXOC4 | 1.40845369 | 8.94E-11 |
| SIGMAR1 | 7.83202461 | 8.99E-11 |
| FAM171A1 | 3.88303259 | 9.07E-11 |
| PHYH | 3.3787325 | 9.09E-11 |
| ENKD1 | -1.6633409 | 9.17E-11 |
| HNRNPA0 | -3.1247956 | 9.45E-11 |
| RIOK3 | 2.48672615 | 9.58E-11 |
| PPP1R12C | -1.6805032 | 9.72E-11 |
| NAMPT | 5.09152701 | 9.77E-11 |
| KLF11 | 1.37205668 | 9.98E-11 |
| PRRC1 | 2.30301219 | 1.01E-10 |
| CAD | 2.00569188 | 1.02E-10 |
| CKS2 | 21.6252122 | 1.03E-10 |
| NCL | 11.2620798 | 1.05E-10 |
| AK4 | 2.5870874 | 1.08E-10 |
| COQ9 | 1.5766415 | 1.08E-10 |
| SHMT2 | 4.69757733 | 1.08E-10 |
| TWF1 | 4.04728116 | 1.09E-10 |
| RIPOR1 | -1.1979796 | 1.10E-10 |
| D2HGDH | -1.4680274 | 1.10E-10 |

| | | |
|---|---|---|
| CDH5 | -2.3632019 | 1.10E-10 |
| TPM3 | 5.66352341 | 1.12E-10 |
| C12orf57 | -4.324716 | 1.13E-10 |
| GGH | 1.93948892 | 1.13E-10 |
| DAPK3 | -3.9046194 | 1.17E-10 |
| COL5A1 | -18.35737 | 1.20E-10 |
| MOB3A | -1.9851675 | 1.20E-10 |
| OTUD4 | 1.17162748 | 1.20E-10 |
| MRPL54 | -7.4987578 | 1.22E-10 |
| VEGFB | -8.6526975 | 1.22E-10 |
| FAM199X | 1.77987544 | 1.27E-10 |
| TBC1D22B | 1.1489252 | 1.29E-10 |
| TNRC18 | -3.245812 | 1.34E-10 |
| APOO | 2.27802887 | 1.36E-10 |
| IER2 | -12.159041 | 1.40E-10 |
| GIGYF1 | -2.738378 | 1.40E-10 |
| NNMT | -14.711136 | 1.41E-10 |
| ATL3 | 3.3650219 | 1.42E-10 |
| STIP1 | 10.271594 | 1.43E-10 |
| ATP1A1 | 8.99740293 | 1.50E-10 |
| NFIC | -3.942995 | 1.50E-10 |
| OXSR1 | 1.54923082 | 1.53E-10 |
| UGT8 | 2.25780176 | 1.54E-10 |
| FKBP8 | -18.099137 | 1.55E-10 |
| ERCC1 | -1.7117225 | 1.55E-10 |
| SELENOO | -2.1888068 | 1.56E-10 |
| SORT1 | 4.47632714 | 1.60E-10 |
| YARS2 | 1.45005955 | 1.65E-10 |
| BUD31 | 2.06304548 | 1.66E-10 |

| | | |
|---|---|---|
| ENAH | 5.15107355 | 1.71E-10 |
| SUCLG1 | 2.38673265 | 1.75E-10 |
| FNDC3B | 2.26627923 | 1.76E-10 |
| MCM2 | 2.74449089 | 1.78E-10 |
| COL6A2 | -93.322045 | 1.81E-10 |
| ASS1 | 18.9572126 | 1.81E-10 |
| ME1 | 3.91549291 | 1.81E-10 |
| ROGDI | -1.6502196 | 1.82E-10 |
| WNK1 | 4.03309499 | 1.83E-10 |
| CSRNP1 | -4.9095004 | 1.83E-10 |
| SYTL1 | -2.0394498 | 1.84E-10 |
| NME3 | -18.307671 | 1.88E-10 |
| GEMIN4 | 1.06769538 | 1.92E-10 |
| HDGFL2 | -3.3413957 | 1.96E-10 |
| IQCB1 | 1.68883626 | 1.98E-10 |
| EIF3B | 5.12378122 | 2.00E-10 |
| AGPAT5 | 1.68638455 | 2.01E-10 |
| CABLES2 | 1.58201589 | 2.02E-10 |
| CHCHD4 | 1.68428593 | 2.05E-10 |
| EMC8 | 1.10787374 | 2.12E-10 |
| IQCG | 1.06231559 | 2.16E-10 |
| LMAN1 | 6.99527765 | 2.17E-10 |
| CAPN7 | 1.91977648 | 2.17E-10 |
| NPDC1 | -8.5998056 | 2.22E-10 |
| ARF4 | 16.7293062 | 2.23E-10 |
| CD63 | -47.2511 | 2.24E-10 |
| CDCP1 | 2.59395473 | 2.25E-10 |
| WARS1 | 11.17398 | 2.26E-10 |
| POLR3F | 1.17857422 | 2.26E-10 |

| | | |
|---|---|---|
| RPS6KC1 | 1.24277573 | 2.29E-10 |
| LRRC42 | 3.4956564 | 2.32E-10 |
| PRKX | 2.44436294 | 2.33E-10 |
| SRPRA | 8.3845798 | 2.34E-10 |
| MZT2B | -8.360149 | 2.48E-10 |
| GHITM | 17.5057461 | 2.50E-10 |
| KPNA6 | 2.30300642 | 2.52E-10 |
| DERL1 | 4.16335789 | 2.55E-10 |
| BUD13 | 1.47479651 | 2.57E-10 |
| BIRC5 | 6.75040808 | 2.60E-10 |
| WNK4 | -4.1295222 | 2.60E-10 |
| FBXL15 | -1.5314426 | 2.64E-10 |
| LARP1 | 4.20707494 | 2.71E-10 |
| TRIM25 | 1.33096339 | 2.74E-10 |
| DGCR6L | -8.1528399 | 2.75E-10 |
| DNMT1 | 1.86986252 | 2.90E-10 |
| TRIM14 | 2.89668707 | 2.92E-10 |
| MOB2 | -1.5459487 | 2.92E-10 |
| SGSH | -1.159704 | 2.97E-10 |
| HOXD9 | -1.2064991 | 2.99E-10 |
| KCTD5 | 2.69915423 | 3.01E-10 |
| BRI3BP | 1.43858435 | 3.04E-10 |
| USP47 | 1.03621752 | 3.06E-10 |
| DNAJA3 | 1.96857346 | 3.10E-10 |
| ATG16L2 | -1.479992 | 3.10E-10 |
| ZNF639 | 1.305878 | 3.11E-10 |
| CAMSAP1 | 1.00621643 | 3.12E-10 |
| NNT | 1.36146831 | 3.27E-10 |
| HECA | 1.86478748 | 3.30E-10 |

| | | |
|---|---|---|
| FLYWCH2 | -7.0122899 | 3.32E-10 |
| HDGF | 26.7469241 | 3.37E-10 |
| RPL7L1 | 5.27603847 | 3.37E-10 |
| RAB3D | 3.09562968 | 3.42E-10 |
| USP34 | 1.52686887 | 3.57E-10 |
| B4GALT7 | -1.2225485 | 3.58E-10 |
| GANAB | 16.0677767 | 3.59E-10 |
| NOL6 | 1.65922017 | 3.65E-10 |
| SMPD4BP | -2.1313031 | 3.70E-10 |
| CEP350 | 1.62401855 | 3.87E-10 |
| PTS | 1.32483245 | 4.04E-10 |
| TMEM33 | 1.82152083 | 4.09E-10 |
| WDR53 | 1.09568322 | 4.10E-10 |
| YWHAE | 23.3638386 | 4.14E-10 |
| SBDS | 6.08816916 | 4.29E-10 |
| HNRNPR | 2.32122755 | 4.32E-10 |
| EIF4H | 4.38673309 | 4.40E-10 |
| SLC35C1 | 2.50128205 | 4.46E-10 |
| RPL36AL | -43.190428 | 4.51E-10 |
| MRPS9 | 1.41694705 | 4.65E-10 |
| NAT10 | 3.77756733 | 4.65E-10 |
| GNPTAB | 1.70120192 | 4.65E-10 |
| NSA2 | -3.0082907 | 4.69E-10 |
| SIGIRR | -4.7136154 | 4.78E-10 |
| MRPL15 | 9.83527517 | 4.79E-10 |
| RNH1 | -3.8570663 | 4.84E-10 |
| RSPRY1 | 1.23765683 | 4.85E-10 |
| COL1A2 | -210.43882 | 5.04E-10 |
| CDK6 | 2.01840284 | 5.06E-10 |

| | | |
|---|---|---|
| CCT3 | 22.4935383 | 5.17E-10 |
| PAPOLA | 2.29641079 | 5.28E-10 |
| BIRC6 | 1.85911384 | 5.42E-10 |
| C1GALT1 | 1.36526921 | 5.50E-10 |
| CHMP3 | 1.63177423 | 5.58E-10 |
| HSPB6 | -1.0436627 | 5.68E-10 |
| RALA | 3.36925274 | 5.80E-10 |
| CREB3L2 | 5.17271221 | 5.80E-10 |
| CARMIL1 | 1.33516869 | 5.82E-10 |
| SEMA3F | -6.3894165 | 5.90E-10 |
| SLC30A9 | 2.07194803 | 6.01E-10 |
| TSPAN7 | -1.3873707 | 6.02E-10 |
| CILP | -16.459244 | 6.07E-10 |
| SCOC | 2.5830698 | 6.09E-10 |
| DELE1 | -1.4308992 | 6.17E-10 |
| PLK3 | -1.2912437 | 6.48E-10 |
| NIPAL2 | 2.4892746 | 6.48E-10 |
| SLC17A5 | 2.34597385 | 6.49E-10 |
| EFTUD2 | 1.78386819 | 6.56E-10 |
| CDCA3 | 1.39335568 | 6.60E-10 |
| RAB14 | 5.17331662 | 6.65E-10 |
| YWHAB | 9.42820179 | 6.93E-10 |
| IQGAP3 | 2.5976589 | 6.93E-10 |
| TIMMDC1 | 3.15108145 | 6.99E-10 |
| TUBGCP3 | 1.08624474 | 7.11E-10 |
| MRPL43 | -2.0462926 | 7.22E-10 |
| BTBD1 | 2.84473859 | 7.24E-10 |
| SRPRB | 3.73166567 | 7.34E-10 |
| NCSTN | 8.40973576 | 7.73E-10 |

| | | |
|---|---|---|
| CEP70 | 1.19100284 | 7.75E-10 |
| EIF2B3 | 1.29092198 | 7.83E-10 |
| PAK2 | 4.63854064 | 7.85E-10 |
| TENT4A | 1.10586621 | 7.85E-10 |
| RPL15 | -15.988837 | 7.88E-10 |
| PABPC1 | 52.7042786 | 8.02E-10 |
| TUBG2 | -1.5956254 | 8.22E-10 |
| SNRPA1 | 2.31645517 | 8.38E-10 |
| RCC2 | 8.08349302 | 8.43E-10 |
| NUMA1 | -4.4311877 | 8.48E-10 |
| SEMA3G | -2.3300873 | 8.52E-10 |
| MAPK1 | 3.88944433 | 8.60E-10 |
| PRPS1 | 2.38027704 | 8.61E-10 |
| CDK2AP1 | 9.35385792 | 8.62E-10 |
| TMEM267 | 1.13191731 | 8.66E-10 |
| ECHDC2 | -1.6427247 | 8.71E-10 |
| TCF20 | 1.75809191 | 8.74E-10 |
| UBR2 | 2.00816888 | 8.91E-10 |
| NELFA | -1.1084365 | 8.97E-10 |
| PSMA4 | 3.41401517 | 8.99E-10 |
| DCK | 1.98636089 | 9.06E-10 |
| HEYL | -1.6823006 | 9.09E-10 |
| RCC1 | 3.69499485 | 9.10E-10 |
| AHSA2P | -1.1471222 | 9.12E-10 |
| SH2B1 | -1.7138206 | 9.23E-10 |
| ZBTB5 | 1.13497904 | 9.46E-10 |
| DDX46 | 1.34853325 | 9.56E-10 |
| CENPH | 1.1371432 | 9.57E-10 |
| RRN3 | 3.30387247 | 9.57E-10 |

| | | |
|---|---|---|
| NFIL3 | 4.05339399 | 9.62E-10 |
| COMP | -25.681686 | 9.65E-10 |
| FBLN1 | -12.276082 | 9.73E-10 |
| GBP1 | 9.95493948 | 1.00E-09 |
| CUL3 | 1.03098233 | 1.01E-09 |
| POLRMT | -1.5819977 | 1.01E-09 |
| ARHGEF1 | -2.6523386 | 1.01E-09 |
| CAND1 | 4.27257041 | 1.01E-09 |
| FGD3 | -6.4660946 | 1.02E-09 |
| RAF1 | 1.10518555 | 1.02E-09 |
| FAM110B | -2.3074006 | 1.05E-09 |
| UBN1 | 1.93724638 | 1.08E-09 |
| USP20 | -1.1084828 | 1.09E-09 |
| SEC63 | 4.71473259 | 1.09E-09 |
| CAPZB | -1.8560858 | 1.09E-09 |
| DCN | -18.161257 | 1.09E-09 |
| ADNP | 4.06940001 | 1.09E-09 |
| CPSF6 | 1.59165504 | 1.11E-09 |
| TMEM38A | 1.28468557 | 1.12E-09 |
| CDK16 | 5.26083598 | 1.15E-09 |
| RPL11 | -57.13756 | 1.15E-09 |
| TULP3 | 2.04705653 | 1.17E-09 |
| PCNP | 4.86354864 | 1.17E-09 |
| IPO5 | 4.12377075 | 1.17E-09 |
| TP53BP2 | 3.70651912 | 1.18E-09 |
| CACUL1 | 1.33260741 | 1.20E-09 |
| XPNPEP1 | 1.23595028 | 1.23E-09 |
| MGST1 | 7.59442616 | 1.25E-09 |
| ELAVL1 | 1.60938617 | 1.26E-09 |

| | | |
|---|---|---|
| SERTAD4 | 3.6329808 | 1.27E-09 |
| DYRK1B | -2.6133832 | 1.28E-09 |
| MOB4 | 1.30731994 | 1.29E-09 |
| ZNF428 | -2.5897776 | 1.29E-09 |
| OAF | -2.9455363 | 1.32E-09 |
| CLOCK | 1.07174471 | 1.33E-09 |
| MIA2 | 1.05352424 | 1.35E-09 |
| BLOC1S1 | -1.0528934 | 1.35E-09 |
| LAP3 | 8.19444234 | 1.39E-09 |
| DESI1 | 1.64130193 | 1.39E-09 |
| PPP4R2 | 1.77801622 | 1.39E-09 |
| MARVELD1 | -4.5065524 | 1.41E-09 |
| PERP | 24.1729258 | 1.43E-09 |
| ME3 | -1.3728215 | 1.43E-09 |
| MISP | -6.1581133 | 1.44E-09 |
| LAD1 | 7.09679934 | 1.44E-09 |
| ZBTB4 | -3.6242298 | 1.48E-09 |
| GEM | -5.5220074 | 1.49E-09 |
| TNN | -1.4718037 | 1.50E-09 |
| RAB5A | 2.24731339 | 1.50E-09 |
| KDM5A | 1.59797986 | 1.52E-09 |
| RPL31 | -26.196489 | 1.53E-09 |
| RAB29 | 2.63735528 | 1.54E-09 |
| CTBP1 | -1.2744323 | 1.59E-09 |
| SEL1L3 | 2.42153938 | 1.60E-09 |
| MTHFD1L | 2.17598039 | 1.60E-09 |
| PATZ1 | -3.1383004 | 1.63E-09 |
| DERA | 2.34503423 | 1.67E-09 |
| RASIP1 | -1.1058694 | 1.68E-09 |

| | | |
|---|---|---|
| ZNF385A | -7.6392488 | 1.69E-09 |
| MED30 | 2.2628976 | 1.72E-09 |
| AIF1L | 6.63163 | 1.73E-09 |
| CLUH | 2.99917235 | 1.73E-09 |
| SPTY2D1 | 1.50954672 | 1.75E-09 |
| DNAJB6 | 1.33648495 | 1.77E-09 |
| ID3 | -8.362435 | 1.78E-09 |
| RSU1 | 7.13894454 | 1.81E-09 |
| RNF24 | 1.95333376 | 1.81E-09 |
| TSC22D3 | -9.4366635 | 1.83E-09 |
| CCDC47 | 8.38937793 | 1.83E-09 |
| PPP1R15A | -5.928749 | 1.83E-09 |
| NEK7 | 2.38866338 | 1.83E-09 |
| SLC35A5 | 1.14727099 | 1.83E-09 |
| ASAP1 | 1.75498592 | 1.89E-09 |
| PICALM | 5.14092065 | 1.89E-09 |
| RPL28 | -32.202331 | 1.91E-09 |
| FLVCR1 | 1.07927202 | 1.92E-09 |
| NR1H2 | -2.100015 | 1.95E-09 |
| UTP23 | 1.82459685 | 1.98E-09 |
| ATP5F1D | -8.4857371 | 2.01E-09 |
| TYMS | 1.1187308 | 2.04E-09 |
| E2F2 | 1.20190161 | 2.04E-09 |
| GCNT2 | 1.35688987 | 2.09E-09 |
| CCDC85B | -8.3036023 | 2.10E-09 |
| ETV3 | 2.02422923 | 2.16E-09 |
| KANSL2 | 1.01840791 | 2.19E-09 |
| RPN1 | 15.2173877 | 2.21E-09 |
| FAAH | -4.2680226 | 2.21E-09 |

| | | |
|---|---|---|
| ZFP36 | -44.74759 | 2.21E-09 |
| DOP1B | 2.66696602 | 2.23E-09 |
| TRAM1 | 14.9996079 | 2.29E-09 |
| WASL | 3.69135336 | 2.33E-09 |
| SF3B6 | 12.7419799 | 2.39E-09 |
| AAAS | -1.1660797 | 2.43E-09 |
| PADI2 | 11.7285495 | 2.48E-09 |
| SEC24D | 2.60653818 | 2.53E-09 |
| RPN2 | 53.5030334 | 2.59E-09 |
| MRPS33 | 1.25666345 | 2.62E-09 |
| LIPE | -2.4909914 | 2.66E-09 |
| UBXN7 | 1.21696237 | 2.68E-09 |
| GMNN | 2.25868255 | 2.73E-09 |
| CALM2 | 12.966127 | 2.73E-09 |
| B4GALNT4 | -3.0931359 | 2.74E-09 |
| OVOL2 | -1.4164239 | 2.77E-09 |
| HOXC9 | -1.521493 | 2.86E-09 |
| C1orf198 | 6.69465794 | 2.94E-09 |
| TOP2B | 6.21680856 | 2.96E-09 |
| KDM7A | 1.47102519 | 3.12E-09 |
| ST14 | 13.3172128 | 3.16E-09 |
| EGR1 | -50.190739 | 3.16E-09 |
| FGF1 | -1.0772415 | 3.18E-09 |
| SIRT3 | -1.1574814 | 3.19E-09 |
| COL3A1 | -282.14163 | 3.21E-09 |
| GAPDH | 161.371677 | 3.24E-09 |
| RPL13 | -62.657382 | 3.31E-09 |
| UQCRH | 18.6441719 | 3.33E-09 |
| B3GALT6 | -2.4648072 | 3.36E-09 |

| | | |
|---|---|---|
| PLA2G7 | 2.38513443 | 3.42E-09 |
| FAM167B | -1.4004901 | 3.44E-09 |
| RP2 | 1.0250581 | 3.52E-09 |
| SALL2 | -1.4542414 | 3.52E-09 |
| REST | 1.1878367 | 3.57E-09 |
| CLBA1 | -1.0795019 | 3.60E-09 |
| LENG8 | -5.340415 | 3.61E-09 |
| MRPL47 | 6.97650359 | 3.62E-09 |
| TNIP2 | -2.1532941 | 3.62E-09 |
| LSP1 | -2.6147924 | 3.66E-09 |
| UCK2 | 1.69113214 | 3.67E-09 |
| EDF1 | -43.475621 | 3.71E-09 |
| ZNF664 | 6.15178199 | 3.75E-09 |
| SPTLC1 | 1.72792611 | 3.75E-09 |
| ORAI3 | -1.4861983 | 3.79E-09 |
| PLPP1 | -3.0419888 | 3.82E-09 |
| RB1CC1 | 3.12002752 | 3.99E-09 |
| SFXN3 | -2.1422993 | 4.32E-09 |
| MRPL9 | 3.39944795 | 4.38E-09 |
| TADA3 | -3.9355028 | 4.43E-09 |
| ARHGAP21 | 2.0251261 | 4.45E-09 |
| ZMYM4 | 1.72497038 | 4.47E-09 |
| CUL4A | 2.60090267 | 4.48E-09 |
| ADAMDEC1 | 3.40289417 | 4.49E-09 |
| GSDMD | -4.7032043 | 4.58E-09 |
| CXXC5 | -8.1158877 | 4.59E-09 |
| HDAC5 | -1.7955617 | 4.66E-09 |
| NEDD1 | 1.47646337 | 4.66E-09 |
| CIC | -1.804652 | 4.77E-09 |

| | | |
|---|---|---|
| IVNS1ABP | 3.38740119 | 4.82E-09 |
| LIMS1 | 1.63977083 | 4.82E-09 |
| TNS1 | -2.7211853 | 4.83E-09 |
| NUDT14 | -2.5597755 | 4.87E-09 |
| ITPR3 | 2.67712168 | 4.92E-09 |
| SERP1 | 7.9686529 | 5.05E-09 |
| MCU | 1.0007877 | 5.09E-09 |
| MTRR | 1.66963504 | 5.13E-09 |
| POGLUT3 | 1.92631917 | 5.16E-09 |
| HMG20B | -6.3396159 | 5.18E-09 |
| MAPK8IP3 | -1.526027 | 5.23E-09 |
| MICALL2 | -1.0930482 | 5.32E-09 |
| VANGL1 | 1.38330286 | 5.43E-09 |
| PIGQ | -2.5377506 | 5.45E-09 |
| ATP5MC3 | 6.36513619 | 5.65E-09 |
| SLC66A1 | -1.1892521 | 5.82E-09 |
| NT5C3A | 1.91763576 | 5.90E-09 |
| REEP3 | 3.05957062 | 6.01E-09 |
| MEF2D | -2.6903215 | 6.03E-09 |
| NDUFA2 | -4.0243548 | 6.14E-09 |
| MAGED2 | -92.122559 | 6.39E-09 |
| NLGN2 | -1.2058218 | 6.40E-09 |
| NMI | 2.02784302 | 6.44E-09 |
| GCN1 | 2.40730953 | 6.47E-09 |
| NIPSNAP2 | 3.71136988 | 6.49E-09 |
| JOSD1 | 2.68660914 | 6.60E-09 |
| DCAF1 | 1.04481961 | 6.60E-09 |
| THBD | -2.4519351 | 6.76E-09 |
| RAD21 | 21.675405 | 6.80E-09 |

| | | |
|---|---|---|
| LGALSL | 1.46179439 | 6.81E-09 |
| SRP9 | 18.4986217 | 6.83E-09 |
| BCL7C | -1.4951039 | 6.84E-09 |
| BAD | -1.5899956 | 6.87E-09 |
| PIP5K1A | 3.20097275 | 6.89E-09 |
| MAP3K2 | 1.25205388 | 7.07E-09 |
| PMP22 | -3.3970195 | 7.13E-09 |
| PRDX3 | 8.70288693 | 7.26E-09 |
| RFNG | -2.2214693 | 7.36E-09 |
| RTN2 | -1.580978 | 7.36E-09 |
| RPL13AP25 | -3.7102549 | 7.40E-09 |
| NIPSNAP3A | 1.66871261 | 7.45E-09 |
| MRFAP1L1 | -6.1679248 | 7.56E-09 |
| LYPD6B | -2.5592336 | 7.59E-09 |
| STAT6 | -3.3332304 | 7.74E-09 |
| VWF | -5.8655336 | 7.80E-09 |
| MISP3 | -2.2471929 | 7.92E-09 |
| INTS11 | -1.6517714 | 8.03E-09 |
| FIBIN | -2.9611018 | 8.03E-09 |
| TSPAN33 | 2.3627492 | 8.46E-09 |
| SRD5A1 | 1.81951428 | 8.48E-09 |
| CARD10 | -1.1161394 | 8.64E-09 |
| F2RL2 | -3.899281 | 8.64E-09 |
| CAVIN2 | -1.9861044 | 8.74E-09 |
| SETDB1 | 1.12625265 | 8.80E-09 |
| RNF145 | 3.6927595 | 8.96E-09 |
| TLE5 | -5.8852509 | 8.99E-09 |
| PUM2 | 3.55580204 | 8.99E-09 |
| CUEDC2 | -6.5061298 | 9.04E-09 |

| | | |
|---|---|---|
| NUP188 | 1.4348389 | 9.09E-09 |
| PLSCR4 | -1.3872675 | 9.13E-09 |
| CCN5 | -3.98627 | 9.21E-09 |
| TM9SF4 | 3.2289782 | 9.40E-09 |
| ACADM | 2.19193894 | 9.54E-09 |
| PKN2 | 1.57031018 | 9.57E-09 |
| MRPS18A | 1.58756003 | 9.71E-09 |
| RPL18 | -46.175635 | 9.79E-09 |
| GFM2 | 1.17161963 | 9.81E-09 |
| SMARCAD1 | 1.2024914 | 9.99E-09 |
| TPPP3 | -5.4356703 | 1.01E-08 |
| MANSC1 | 2.75675336 | 1.01E-08 |
| CDC40 | 1.17019607 | 1.05E-08 |
| ITPK1 | -3.1061319 | 1.06E-08 |
| PPA2 | 2.00135312 | 1.08E-08 |
| GLO1 | 15.8243543 | 1.09E-08 |
| PDCD11 | 1.55044968 | 1.12E-08 |
| SMS | 13.4018687 | 1.14E-08 |
| EMC2 | 1.73455929 | 1.14E-08 |
| EZH1 | -1.0471396 | 1.14E-08 |
| ADAM15 | 9.80571039 | 1.19E-08 |
| POLR2K | 9.22939394 | 1.19E-08 |
| PNKP | -1.4488722 | 1.20E-08 |
| ADAM9 | 3.21442542 | 1.22E-08 |
| S1PR1 | -1.7578095 | 1.24E-08 |
| RBBP4 | 2.71322975 | 1.24E-08 |
| SCAF11 | 1.60168903 | 1.25E-08 |
| EIF4EBP2 | 4.08709048 | 1.27E-08 |
| TCEAL4 | -29.306077 | 1.31E-08 |

| | | |
|---|---|---|
| NDUFS2 | 2.97166286 | 1.32E-08 |
| GPC1 | -5.4987184 | 1.33E-08 |
| LRIF1 | 1.09989017 | 1.34E-08 |
| MPHOSPH6 | 3.9380981 | 1.37E-08 |
| RRAS | -8.5980583 | 1.38E-08 |
| BAG5 | 1.9947729 | 1.39E-08 |
| SMYD2 | 2.19773112 | 1.39E-08 |
| UQCRC2 | 5.34226051 | 1.39E-08 |
| PPP1R15B | 3.14602299 | 1.43E-08 |
| STK26 | 3.63171783 | 1.44E-08 |
| TPT1 | -31.2947 | 1.44E-08 |
| CREG1 | 8.0271758 | 1.45E-08 |
| ZFYVE27 | -1.2941988 | 1.45E-08 |
| STEAP3 | 4.12057036 | 1.46E-08 |
| USP38 | 1.22519792 | 1.46E-08 |
| RAB3IL1 | -1.2566797 | 1.53E-08 |
| PSMA3 | 4.54746869 | 1.53E-08 |
| FKBP10 | -11.868275 | 1.54E-08 |
| ANKS1A | 1.16413507 | 1.58E-08 |
| STMN1 | 12.3606251 | 1.59E-08 |
| PHF5A | 3.46515032 | 1.61E-08 |
| PDP1 | 2.52148283 | 1.64E-08 |
| ELOF1 | -2.1028441 | 1.65E-08 |
| R3HCC1 | -2.1600543 | 1.69E-08 |
| EIF5B | 4.35677776 | 1.70E-08 |
| RHOT2 | -3.8631792 | 1.71E-08 |
| FOXRED2 | 2.52776287 | 1.73E-08 |
| IL12RB2 | 1.38760613 | 1.74E-08 |
| RASD2 | 4.18502956 | 1.74E-08 |

| | | |
|---|---|---|
| RPL7A | -86.016251 | 1.74E-08 |
| SAMD9L | 3.17245016 | 1.76E-08 |
| LETMD1 | -1.1993882 | 1.80E-08 |
| EPS15 | 1.54900863 | 1.80E-08 |
| METAP1 | 1.84064517 | 1.82E-08 |
| NCS1 | 1.92520447 | 1.82E-08 |
| ROCK1 | 1.25299695 | 1.89E-08 |
| TMEM159 | 4.04210069 | 1.94E-08 |
| SRP14 | -14.31727 | 1.96E-08 |
| ERLEC1 | 4.92497065 | 1.96E-08 |
| GMDS | 2.580523 | 1.96E-08 |
| SERF2 | -6.2852067 | 2.00E-08 |
| ISM1 | -1.6165879 | 2.03E-08 |
| GLT8D1 | -1.8362097 | 2.03E-08 |
| TMTC3 | 1.4250403 | 2.03E-08 |
| STX4 | -2.1468625 | 2.03E-08 |
| ZDHHC5 | 3.82480059 | 2.03E-08 |
| SAMHD1 | 2.48595379 | 2.04E-08 |
| UBE2N | 2.72076247 | 2.04E-08 |
| ERF | -2.9199077 | 2.04E-08 |
| FAU | -32.179548 | 2.04E-08 |
| USP18 | 3.02903492 | 2.05E-08 |
| CDC25B | 5.78892393 | 2.09E-08 |
| SCARB2 | 2.85079522 | 2.09E-08 |
| MKRN1 | 3.27477771 | 2.10E-08 |
| GPR68 | -1.1376261 | 2.10E-08 |
| MTMR6 | 1.25984611 | 2.13E-08 |
| CRTAP | -3.2049206 | 2.13E-08 |
| PNMA1 | -3.3459066 | 2.16E-08 |

| | | |
|---|---|---|
| HSP90AA1 | 78.073855 | 2.16E-08 |
| CDC42EP5 | -2.4119832 | 2.19E-08 |
| MICOS13 | -6.5900835 | 2.28E-08 |
| TMEM101 | -10.289676 | 2.28E-08 |
| GPX4 | -30.056554 | 2.30E-08 |
| CRISPLD2 | -3.8294146 | 2.33E-08 |
| CCNT1 | 1.06378524 | 2.34E-08 |
| RPS11 | -156.71571 | 2.36E-08 |
| SRPX | -3.5189763 | 2.36E-08 |
| IDH1 | 8.11990765 | 2.37E-08 |
| MAP3K7 | 1.25796794 | 2.41E-08 |
| ISCU | -1.9627744 | 2.42E-08 |
| E4F1 | -1.4815467 | 2.44E-08 |
| AKAP8L | -1.2254491 | 2.50E-08 |
| RANBP1 | 2.96452222 | 2.50E-08 |
| TRIAP1 | 2.8425008 | 2.56E-08 |
| POR | 3.3660579 | 2.56E-08 |
| THAP7 | -1.9415672 | 2.59E-08 |
| MBD4 | 1.87784737 | 2.59E-08 |
| PRPF38A | 1.3429087 | 2.65E-08 |
| BRCC3 | 4.60271186 | 2.68E-08 |
| CRIP2 | -17.790786 | 2.69E-08 |
| TCEA2 | -2.1266516 | 2.72E-08 |
| ZNF524 | -2.8068622 | 2.73E-08 |
| BGN | -121.96993 | 2.76E-08 |
| PTPN18 | -2.5985395 | 2.77E-08 |
| MYL6 | -21.454487 | 2.79E-08 |
| VPS4B | 2.92334647 | 2.80E-08 |
| GADD45G | -6.1135063 | 2.80E-08 |

| | | |
|---|---|---|
| SQLE | 9.92975621 | 2.85E-08 |
| DIP2B | 1.54969328 | 2.91E-08 |
| NECTIN2 | -13.122639 | 2.95E-08 |
| CDT1 | 2.4334051 | 2.99E-08 |
| CENPU | 2.20982366 | 3.03E-08 |
| HORMAD1 | 2.3720321 | 3.03E-08 |
| STRBP | 1.45819599 | 3.04E-08 |
| NFYA | 4.48089048 | 3.13E-08 |
| SLC7A8 | -8.1783416 | 3.14E-08 |
| BAIAP2L1 | 1.58106552 | 3.14E-08 |
| SPTSSA | 8.00275985 | 3.16E-08 |
| SLIT3 | -1.058283 | 3.17E-08 |
| CAV1 | -7.4812365 | 3.20E-08 |
| SRP54 | 1.60834932 | 3.23E-08 |
| PTER | 1.28439213 | 3.24E-08 |
| CPXM1 | -9.9745834 | 3.32E-08 |
| UHRF1BP1L | 1.01539374 | 3.33E-08 |
| OAS3 | 7.75807777 | 3.34E-08 |
| STK24 | 1.18461421 | 3.37E-08 |
| KIF12 | -9.5479089 | 3.40E-08 |
| CIDEA | -1.3234618 | 3.42E-08 |
| MYORG | 2.24173742 | 3.45E-08 |
| POLR2C | 2.54605044 | 3.47E-08 |
| SEMA3B | -3.9949343 | 3.52E-08 |
| VPS51 | -2.391477 | 3.57E-08 |
| KCTD9 | 1.64407336 | 3.57E-08 |
| TSPAN9 | -2.3893852 | 3.62E-08 |
| MRPL30 | 1.00793432 | 3.63E-08 |
| TMEM263 | 3.04153771 | 3.64E-08 |

| | | |
|---|---|---|
| SFRP4 | -17.540762 | 3.65E-08 |
| BAK1 | 1.96095943 | 3.66E-08 |
| DNAJB2 | -3.3537521 | 3.67E-08 |
| TRIM45 | -1.2343293 | 3.71E-08 |
| HMGB3 | 7.58810693 | 3.77E-08 |
| MBD3 | -1.039375 | 3.78E-08 |
| MED15 | -1.0584106 | 3.83E-08 |
| OCEL1 | -3.0061683 | 3.84E-08 |
| MEOX1 | -1.1461286 | 3.89E-08 |
| UBP1 | 1.74711212 | 3.95E-08 |
| SLC18B1 | 1.69601559 | 3.97E-08 |
| RETREG2 | -3.2541663 | 4.07E-08 |
| ADM2 | 1.38301149 | 4.15E-08 |
| EMCN | -1.100224 | 4.15E-08 |
| TMEM219 | -10.418821 | 4.16E-08 |
| KRAS | 1.99178801 | 4.18E-08 |
| PSMD11 | 4.78010275 | 4.19E-08 |
| AIP | -6.5243588 | 4.23E-08 |
| TIMP1 | -80.258103 | 4.26E-08 |
| ABCA12 | 2.25028427 | 4.27E-08 |
| STING1 | -2.0752109 | 4.31E-08 |
| TDP2 | 2.10626897 | 4.32E-08 |
| LMO2 | -1.2720379 | 4.38E-08 |
| CAB39 | 2.40957653 | 4.39E-08 |
| ACIN1 | -2.2687994 | 4.39E-08 |
| ARVCF | -1.0738967 | 4.47E-08 |
| RNMT | 1.32576665 | 4.48E-08 |
| MRC2 | -10.114042 | 4.64E-08 |
| SMAD3 | -1.6835813 | 4.72E-08 |

| | | |
|---|---|---|
| PM20D2 | 1.67860245 | 4.86E-08 |
| GTF2A1 | 1.28511441 | 4.88E-08 |
| OAS2 | 10.2974389 | 4.93E-08 |
| DOK1 | -1.8773373 | 4.97E-08 |
| NCOA7 | 3.04500827 | 5.00E-08 |
| P3H4 | -2.746226 | 5.12E-08 |
| AZIN1 | 10.9919123 | 5.16E-08 |
| PER1 | -2.5651783 | 5.17E-08 |
| NASP | 2.90224896 | 5.18E-08 |
| FAAP100 | -1.8401348 | 5.18E-08 |
| SMDT1 | -3.8911983 | 5.21E-08 |
| OGDH | 3.49087545 | 5.23E-08 |
| USF2 | -3.8488122 | 5.23E-08 |
| UBFD1 | 1.96245681 | 5.26E-08 |
| PLRG1 | 1.47617282 | 5.48E-08 |
| CCR1 | 2.03698251 | 5.51E-08 |
| IDH2 | 26.2582317 | 5.56E-08 |
| LMO4 | 7.24270791 | 5.65E-08 |
| HOOK2 | -1.7679342 | 5.67E-08 |
| GNL3 | 4.36591531 | 5.68E-08 |
| SRPX2 | -1.0124412 | 5.69E-08 |
| CSAD | -2.6124391 | 5.71E-08 |
| CFAP20 | 1.4621448 | 5.76E-08 |
| SLC16A1 | 3.90601531 | 5.79E-08 |
| CHRD | -2.5580999 | 5.87E-08 |
| ACAT1 | 1.70899041 | 5.96E-08 |
| ZDHHC8 | -1.0694776 | 6.10E-08 |
| SERTAD2 | 1.82434685 | 6.11E-08 |
| SETD5 | 1.89937671 | 6.23E-08 |

| | | |
|---|---|---|
| SEMA6B | -1.434736 | 6.23E-08 |
| ZFAND1 | 1.96324013 | 6.25E-08 |
| CNOT6 | 1.79879405 | 6.27E-08 |
| DNMBP | -1.1306568 | 6.29E-08 |
| PER2 | -1.1427535 | 6.35E-08 |
| XRCC1 | -1.9693611 | 6.38E-08 |
| TPRN | -2.3341085 | 6.48E-08 |
| FLAD1 | 3.38341383 | 6.48E-08 |
| PTMS | -65.498971 | 6.51E-08 |
| TSSC4 | -2.970567 | 6.51E-08 |
| PTTG1 | 4.58514965 | 6.54E-08 |
| CXCL12 | -6.0602093 | 6.54E-08 |
| FDFT1 | 6.07791517 | 6.61E-08 |
| KDM1A | 3.0357886 | 6.71E-08 |
| FCF1 | 1.09400957 | 6.82E-08 |
| AFAP1L2 | -2.4057011 | 6.97E-08 |
| MMP19 | -1.0362686 | 6.99E-08 |
| LAMP3 | 3.76140152 | 7.14E-08 |
| RNF19A | 4.14059301 | 7.15E-08 |
| GPR107 | 2.64795209 | 7.15E-08 |
| EML2 | -1.5749904 | 7.31E-08 |
| SLC26A11 | -1.1247877 | 7.35E-08 |
| TEX264 | -2.2564304 | 7.40E-08 |
| FUT3 | 1.61711511 | 7.45E-08 |
| NDFIP2 | 1.31806431 | 7.50E-08 |
| CBX5 | 2.24698078 | 7.50E-08 |
| PGLS | -3.9043492 | 7.50E-08 |
| C8orf33 | 4.35486781 | 7.52E-08 |
| SEC14L2 | -6.2233245 | 7.61E-08 |

| | | |
|---|---|---|
| ZNF12 | 1.0320972 | 7.62E-08 |
| CSTF2 | 1.32308828 | 7.62E-08 |
| MAD2L1BP | 1.95571941 | 7.71E-08 |
| RRAS2 | 1.13460846 | 7.88E-08 |
| COL6A3 | -20.83859 | 7.89E-08 |
| PRDM4 | 1.233929 | 7.94E-08 |
| TRMT2A | -1.4420179 | 8.01E-08 |
| EEF2 | -108.80364 | 8.06E-08 |
| POLR2E | -3.8146404 | 8.28E-08 |
| TRAPPC6A | -9.1125096 | 8.30E-08 |
| ADK | 1.38957619 | 8.37E-08 |
| SCPEP1 | 9.38047912 | 8.52E-08 |
| COX7A1 | -8.2180435 | 8.55E-08 |
| OPTN | 4.74134165 | 8.65E-08 |
| LYAR | 1.38676182 | 8.66E-08 |
| PPIL4 | 1.52296753 | 8.91E-08 |
| COL5A3 | -3.5862179 | 8.93E-08 |
| RNF213 | 3.30265629 | 8.97E-08 |
| CAVIN3 | -1.0868975 | 9.01E-08 |
| LRRC59 | 12.8988522 | 9.06E-08 |
| RFK | 1.4574615 | 9.15E-08 |
| DRG1 | 4.13816653 | 9.38E-08 |
| PIH1D1 | -3.0529775 | 9.38E-08 |
| UPF3B | 1.06723962 | 9.46E-08 |
| ACOX1 | 1.73066767 | 9.63E-08 |
| RAB13 | -1.7971469 | 9.66E-08 |
| SEC24C | 2.64298758 | 9.67E-08 |
| SOD2 | 7.43586658 | 9.69E-08 |
| LYPLA1 | 6.65685935 | 9.74E-08 |

| | | |
|---|---|---|
| RNASEH2C | -1.2926038 | 9.88E-08 |
| FBXO27 | 1.39601065 | 9.94E-08 |
| HSD3B7 | -2.3508514 | 1.00E-07 |
| ARNTL2 | 1.74062923 | 1.01E-07 |
| PARD3 | 1.75028594 | 1.03E-07 |
| NPRL2 | -1.216858 | 1.03E-07 |
| GPBP1L1 | 2.15221024 | 1.03E-07 |
| REX1BD | -1.9502013 | 1.05E-07 |
| JADE2 | -1.4220977 | 1.06E-07 |
| CWC22 | 1.06025493 | 1.09E-07 |
| USP5 | 5.19705846 | 1.09E-07 |
| BOK | -3.2098466 | 1.10E-07 |
| GLE1 | 2.09977125 | 1.10E-07 |
| YIPF6 | 1.79534625 | 1.12E-07 |
| SLC22A18 | -1.4046022 | 1.12E-07 |
| USP13 | 1.22292137 | 1.16E-07 |
| PBRM1 | 1.3726144 | 1.16E-07 |
| INTS14 | 1.51773188 | 1.19E-07 |
| PPDPF | -84.508053 | 1.19E-07 |
| OLFML2B | -8.5274471 | 1.20E-07 |
| ZER1 | -2.3518968 | 1.23E-07 |
| SLC35B2 | 5.894476 | 1.24E-07 |
| ABLIM1 | 2.67530055 | 1.25E-07 |
| CDIPT | -5.080854 | 1.26E-07 |
| SPAG9 | 2.06459807 | 1.28E-07 |
| EIF3F | -1.2574628 | 1.28E-07 |
| XRCC5 | 8.03351236 | 1.28E-07 |
| CAPRIN1 | 7.68038866 | 1.30E-07 |
| OAZ1 | -15.68656 | 1.30E-07 |

| | | |
|---|---|---|
| EMP3 | -5.153882 | 1.32E-07 |
| COMMD4 | -1.6621935 | 1.32E-07 |
| HPN | -4.6907771 | 1.34E-07 |
| PDCD5 | 5.96517233 | 1.35E-07 |
| ZNF362 | -1.8533137 | 1.36E-07 |
| SELENOF | 8.16375215 | 1.38E-07 |
| WDR75 | 1.57591849 | 1.41E-07 |
| B3GNT3 | 1.9166323 | 1.41E-07 |
| INPP5J | -3.6269216 | 1.41E-07 |
| DCAKD | -2.9229338 | 1.42E-07 |
| MLX | 1.26492091 | 1.43E-07 |
| YKT6 | 2.39837751 | 1.49E-07 |
| PAPSS1 | 9.60043595 | 1.50E-07 |
| MPG | -2.4964286 | 1.53E-07 |
| MPC1 | 3.08602008 | 1.56E-07 |
| CCDC74B | -1.1415116 | 1.56E-07 |
| PIR | 2.56419368 | 1.56E-07 |
| CHMP4C | 4.09397732 | 1.57E-07 |
| TMEM205 | -5.6427057 | 1.58E-07 |
| BPNT2 | 4.57574964 | 1.59E-07 |
| OGFRL1 | 3.01348521 | 1.61E-07 |
| C21orf91 | 1.23161983 | 1.65E-07 |
| MAGOH | 4.46924794 | 1.67E-07 |
| WDR18 | -2.9945323 | 1.67E-07 |
| ANXA11 | -3.4216333 | 1.71E-07 |
| GNL2 | 2.27891353 | 1.72E-07 |
| RAB34 | -5.2529654 | 1.72E-07 |
| NCOA3 | 2.18986281 | 1.74E-07 |
| SYF2 | -1.5772952 | 1.74E-07 |

| | | |
|---|---|---|
| GNPAT | 3.27914912 | 1.76E-07 |
| NSMCE1 | -3.5038884 | 1.76E-07 |
| PARP3 | -1.6977254 | 1.76E-07 |
| TMSB15A | 4.72492921 | 1.78E-07 |
| NRBP1 | 2.96001339 | 1.78E-07 |
| WNT4 | -1.2612777 | 1.78E-07 |
| SDHB | 3.40209591 | 1.83E-07 |
| IMPA2 | 4.04060536 | 1.83E-07 |
| SNRPD1 | 2.01107812 | 1.83E-07 |
| MSRB3 | -1.483209 | 1.84E-07 |
| NAGLU | -2.5311732 | 1.93E-07 |
| IFI44 | 11.1290511 | 1.94E-07 |
| ABHD14B | -3.7518017 | 1.94E-07 |
| TMC4 | -9.8544408 | 1.94E-07 |
| NGFR | -2.1624059 | 1.95E-07 |
| AP1S1 | 5.83132812 | 1.95E-07 |
| SPAG7 | -2.2144671 | 1.96E-07 |
| C16orf74 | -1.0799441 | 1.97E-07 |
| PBXIP1 | -7.9161173 | 2.05E-07 |
| CLSTN2 | -11.156421 | 2.06E-07 |
| SRD5A3 | 1.41912227 | 2.08E-07 |
| EPS8L1 | -2.3802896 | 2.08E-07 |
| TRMT1L | 1.24857745 | 2.10E-07 |
| SUPT16H | 3.37876045 | 2.12E-07 |
| CXCL16 | 7.08595644 | 2.15E-07 |
| PIP4K2C | 5.0408254 | 2.17E-07 |
| SPAG5 | 2.25816181 | 2.19E-07 |
| STK4 | 1.07838084 | 2.21E-07 |
| UBAP2L | 4.15764212 | 2.22E-07 |

| | | |
|---|---|---|
| SPIRE1 | 1.0032076 | 2.22E-07 |
| NUCKS1 | 13.4601512 | 2.25E-07 |
| CTSS | 9.39659387 | 2.27E-07 |
| RARS2 | 1.71989152 | 2.32E-07 |
| ZNF513 | -1.1794658 | 2.35E-07 |
| TNFRSF21 | 6.28094565 | 2.35E-07 |
| TMEM184C | 1.32514489 | 2.41E-07 |
| SMU1 | 1.3725754 | 2.41E-07 |
| HMGCL | -1.2466895 | 2.42E-07 |
| CDCA7L | 1.65234213 | 2.43E-07 |
| PSTPIP2 | 1.38065266 | 2.43E-07 |
| TROAP | 1.139425 | 2.44E-07 |
| TUT7 | 1.16205506 | 2.44E-07 |
| POLR2L | -22.346243 | 2.46E-07 |
| SNX12 | 2.56164902 | 2.51E-07 |
| SPRY1 | -3.3479456 | 2.56E-07 |
| MATN3 | -3.0704834 | 2.64E-07 |
| ECE1 | -7.7256984 | 2.65E-07 |
| TCIM | -26.529936 | 2.66E-07 |
| STAG2 | 2.2403581 | 2.70E-07 |
| NDUFA3 | -4.7694898 | 2.70E-07 |
| IFNGR1 | 4.67485156 | 2.72E-07 |
| CXADR | 2.80637492 | 2.72E-07 |
| HAT1 | 1.33667359 | 2.75E-07 |
| DNAJB9 | 3.23118945 | 2.78E-07 |
| ITFG1 | 1.54964421 | 2.80E-07 |
| RAP2B | 1.1059741 | 2.85E-07 |
| CNN2 | -6.9647259 | 2.86E-07 |
| MRPL1 | 1.91366671 | 2.86E-07 |

| | | |
|---|---|---|
| HMGN4 | 6.32333238 | 2.93E-07 |
| TIMP2 | -14.58425 | 2.97E-07 |
| PPID | 1.77353341 | 3.00E-07 |
| ACLY | 7.59365359 | 3.03E-07 |
| BNIP3 | 2.7677012 | 3.05E-07 |
| KMT5A | 1.10200754 | 3.05E-07 |
| UROD | -1.9833422 | 3.07E-07 |
| PPP1R8 | 1.69972159 | 3.08E-07 |
| SH3GLB1 | 2.07386742 | 3.11E-07 |
| SEC24B | 1.58295132 | 3.13E-07 |
| ECM2 | -1.4506205 | 3.16E-07 |
| ABRAXAS2 | 1.25302033 | 3.18E-07 |
| GNA11 | -1.5139082 | 3.20E-07 |
| INTS10 | 1.19295951 | 3.21E-07 |
| TPMT | 2.34740907 | 3.32E-07 |
| RPS27L | -1.5390179 | 3.32E-07 |
| DAPP1 | 1.2330768 | 3.32E-07 |
| DCLRE1B | 1.27199407 | 3.32E-07 |
| ALDH5A1 | 1.03695539 | 3.39E-07 |
| USP12 | 1.27102284 | 3.39E-07 |
| CCDC80 | -6.2114719 | 3.42E-07 |
| FAM189B | 2.50540257 | 3.52E-07 |
| PRRT3 | -1.5717233 | 3.53E-07 |
| KCNK15 | -3.022697 | 3.60E-07 |
| RDH10 | 10.2485428 | 3.66E-07 |
| RNF13 | 2.27644246 | 3.69E-07 |
| DYNC1LI1 | 1.36804196 | 3.70E-07 |
| CCDC59 | 1.05043729 | 3.71E-07 |
| ADO | 1.35429548 | 3.72E-07 |

| | | |
|---|---|---|
| EIF2AK1 | 5.56887795 | 3.72E-07 |
| RAB2A | 6.40205885 | 3.73E-07 |
| BICDL2 | -1.9648861 | 3.74E-07 |
| CCL8 | 3.96451229 | 3.77E-07 |
| ARHGEF17 | -2.1342065 | 3.79E-07 |
| RPL32 | -41.789465 | 3.79E-07 |
| PIK3C2A | 1.67459975 | 3.81E-07 |
| MGLL | -2.2540891 | 3.85E-07 |
| NAA60 | -1.2367574 | 3.89E-07 |
| CMPK2 | 2.3520154 | 3.99E-07 |
| TMF1 | 1.06368757 | 4.00E-07 |
| BMERB1 | -3.5150039 | 4.03E-07 |
| WDR45B | 6.36602397 | 4.07E-07 |
| VPS28 | -8.6707708 | 4.07E-07 |
| LRRC56 | -1.135699 | 4.08E-07 |
| ELOVL1 | 5.82115903 | 4.10E-07 |
| DPY19L4 | 1.2206602 | 4.10E-07 |
| ENY2 | 1.81451913 | 4.12E-07 |
| KRT19 | -218.93377 | 4.14E-07 |
| RHPN1 | -4.147583 | 4.14E-07 |
| ANP32B | 14.2849184 | 4.18E-07 |
| GGCT | 7.59815291 | 4.18E-07 |
| PIK3AP1 | 1.3873953 | 4.19E-07 |
| GNA13 | 3.49857161 | 4.20E-07 |
| TEF | -1.2260918 | 4.26E-07 |
| HMGB1P5 | 2.67601372 | 4.28E-07 |
| NOSTRIN | -2.0475654 | 4.28E-07 |
| METTL26 | -6.8489104 | 4.31E-07 |
| NFIB | 4.02464986 | 4.38E-07 |

| | | |
|---|---|---|
| FERMT1 | 1.01202932 | 4.43E-07 |
| GOT1 | 3.1523279 | 4.47E-07 |
| ZNF516 | -1.0145941 | 4.47E-07 |
| CD151 | -12.354496 | 4.48E-07 |
| TMUB2 | -1.6571515 | 4.50E-07 |
| INPP4B | -1.843979 | 4.53E-07 |
| HSPA5 | 38.1374046 | 4.53E-07 |
| HILPDA | 4.89432005 | 4.56E-07 |
| PHKG2 | -1.0034931 | 4.59E-07 |
| RSRP1 | -1.4237553 | 4.60E-07 |
| BTBD10 | 1.0677029 | 4.66E-07 |
| AKR7A2 | -5.9317947 | 4.71E-07 |
| HNRNPU | 2.81711498 | 4.74E-07 |
| MAP7D1 | -2.8443522 | 4.74E-07 |
| RNF38 | 1.4668075 | 4.75E-07 |
| SUSD3 | -22.27313 | 4.80E-07 |
| UHMK1 | 4.75082569 | 4.83E-07 |
| PPP1R37 | -2.0598724 | 4.88E-07 |
| ETV6 | 3.39517806 | 4.88E-07 |
| STXBP2 | -2.0759829 | 4.89E-07 |
| NR4A1 | -6.1776633 | 4.89E-07 |
| SEC61A1 | 10.8103936 | 4.92E-07 |
| SHLD2 | 1.19144676 | 4.94E-07 |
| CCT7 | 9.02254853 | 4.94E-07 |
| ARPC5L | 2.46533382 | 5.00E-07 |
| TRARG1 | -2.4101759 | 5.02E-07 |
| PFN2 | 7.57858106 | 5.03E-07 |
| PLPPR3 | -2.0811483 | 5.05E-07 |
| F2R | -4.7584319 | 5.06E-07 |

| | | |
|---|---|---|
| IRS1 | -2.4515919 | 5.07E-07 |
| ANTKMT | -2.9579358 | 5.10E-07 |
| RPL35 | -60.140479 | 5.19E-07 |
| ARL3 | -2.150688 | 5.25E-07 |
| USE1 | -1.5430693 | 5.30E-07 |
| MFSD6 | 2.90028126 | 5.33E-07 |
| BATF | -5.4606537 | 5.35E-07 |
| IRX5 | -4.5848904 | 5.36E-07 |
| VEGFC | -1.1776644 | 5.39E-07 |
| DOK7 | -1.532101 | 5.41E-07 |
| PLIN4 | -8.0951627 | 5.42E-07 |
| TAF11 | 2.26390604 | 5.47E-07 |
| MMP14 | -30.916037 | 5.51E-07 |
| HSD17B14 | -3.6865552 | 5.58E-07 |
| GTPBP2 | 2.13933982 | 5.67E-07 |
| MS4A14 | -1.2956089 | 5.71E-07 |
| IRF2BPL | -3.033534 | 5.80E-07 |
| RNPEPL1 | -1.3538702 | 5.89E-07 |
| ERLIN1 | 1.80145294 | 5.93E-07 |
| NDUFB3 | 5.11789937 | 5.95E-07 |
| NXPH4 | 2.86993038 | 5.98E-07 |
| MIB1 | 1.63415043 | 6.05E-07 |
| ASF1B | 4.0473665 | 6.14E-07 |
| PSRC1 | 1.15366311 | 6.16E-07 |
| CNIH4 | 2.32056012 | 6.16E-07 |
| AP3M1 | 3.17582179 | 6.25E-07 |
| TTI1 | 2.59763826 | 6.29E-07 |
| LDHB | 1.17482425 | 6.34E-07 |
| SERPING1 | -8.9727405 | 6.41E-07 |

| | | |
|---|---|---|
| KLF3 | 1.3100599 | 6.48E-07 |
| SUCO | 3.57343379 | 6.49E-07 |
| UBE2Q1 | 3.12948445 | 6.49E-07 |
| FBXO44 | -1.329885 | 6.61E-07 |
| GAL | 2.77244138 | 6.71E-07 |
| ANAPC2 | -1.3528078 | 6.92E-07 |
| UBLCP1 | 1.40649008 | 6.92E-07 |
| MMP11 | -34.190444 | 6.99E-07 |
| BLZF1 | 1.63846445 | 7.00E-07 |
| MED21 | 1.58310696 | 7.00E-07 |
| ATP6V1C2 | 1.02914558 | 7.04E-07 |
| PARP4 | 2.2504077 | 7.04E-07 |
| NUP107 | 1.97425046 | 7.20E-07 |
| PRKAA1 | 1.80635004 | 7.21E-07 |
| CCDC8 | -2.2079186 | 7.23E-07 |
| NVL | 2.09433202 | 7.26E-07 |
| TXNDC15 | -1.4311084 | 7.28E-07 |
| STOML2 | 8.38915721 | 7.30E-07 |
| NCBP2 | 2.66833424 | 7.31E-07 |
| SUDS3 | 1.97132921 | 7.33E-07 |
| CXCL10 | 43.6418081 | 7.45E-07 |
| NSD1 | 1.28118238 | 7.47E-07 |
| C6orf120 | 1.3477354 | 7.48E-07 |
| NOP14 | 1.04603765 | 7.65E-07 |
| LRRC41 | 1.36855312 | 7.65E-07 |
| ITGA7 | -1.0842382 | 7.70E-07 |
| BZW2 | 4.44907691 | 7.70E-07 |
| FCGRT | -10.980556 | 7.82E-07 |
| COX5A | 9.70463829 | 7.88E-07 |

| | | |
|---|---|---|
| PCIF1 | -1.9022062 | 7.89E-07 |
| SART1 | -2.7026294 | 7.95E-07 |
| NADK2 | 1.10830411 | 7.97E-07 |
| ABHD8 | -1.0494751 | 8.17E-07 |
| RGS14 | -1.2818986 | 8.24E-07 |
| ICMT | 2.37666192 | 8.34E-07 |
| ENOPH1 | 4.02077896 | 8.37E-07 |
| PTPRK | 3.71633416 | 8.39E-07 |
| FOXC1 | 5.70931749 | 8.44E-07 |
| CHMP6 | -1.0583702 | 8.57E-07 |
| GPD1 | -3.9769735 | 8.90E-07 |
| GPRC5B | 1.91827955 | 8.99E-07 |
| TREM2 | -4.9161393 | 9.02E-07 |
| RAB22A | 1.6210676 | 9.02E-07 |
| IGF2BP2 | 1.08704958 | 9.19E-07 |
| GRPEL1 | 1.28362167 | 9.26E-07 |
| HOOK1 | 1.76375116 | 9.35E-07 |
| PIK3R3 | 3.76097991 | 9.50E-07 |
| CCS | -4.5652832 | 9.59E-07 |
| ETV7 | 1.29931479 | 9.68E-07 |
| HSPG2 | -4.0274717 | 1.01E-06 |
| ARL6IP1 | 15.0443145 | 1.01E-06 |
| SYDE1 | -1.1067585 | 1.02E-06 |
| GADD45A | -3.5970307 | 1.03E-06 |
| ELOB | -34.608243 | 1.04E-06 |
| G0S2 | -10.986954 | 1.04E-06 |
| CEP20 | 2.90729453 | 1.05E-06 |
| AKAP12 | -1.1432323 | 1.05E-06 |
| ARFGEF2 | 3.41842053 | 1.06E-06 |

| | | |
|---|---|---|
| TELO2 | -2.7904833 | 1.06E-06 |
| RMI2 | 1.4943756 | 1.06E-06 |
| SOX12 | -2.726334 | 1.06E-06 |
| CHMP2A | -13.443789 | 1.07E-06 |
| DNAJC12 | -11.504046 | 1.08E-06 |
| STC2 | -54.435109 | 1.09E-06 |
| ANKRD13D | -1.6907525 | 1.09E-06 |
| METTL5 | 1.06062791 | 1.10E-06 |
| HERC5 | 1.8028961 | 1.11E-06 |
| TMX1 | 1.72419004 | 1.12E-06 |
| SETX | 1.37306659 | 1.12E-06 |
| HIRIP3 | -1.6189475 | 1.12E-06 |
| BTG3 | 3.57345112 | 1.15E-06 |
| PCGF3 | -1.0625895 | 1.16E-06 |
| MACC1 | 1.02112469 | 1.16E-06 |
| NECTIN1 | 2.21863908 | 1.16E-06 |
| DEAF1 | -1.6813281 | 1.16E-06 |
| RABAC1 | -1.8118224 | 1.16E-06 |
| FUS | -5.7843403 | 1.16E-06 |
| TANK | 1.17762515 | 1.17E-06 |
| CIAO2A | 2.32129563 | 1.17E-06 |
| SERTAD3 | -4.6794019 | 1.19E-06 |
| EEF1AKNMT | 2.11730729 | 1.21E-06 |
| B3GNT2 | 2.87895675 | 1.23E-06 |
| CSDE1 | 16.0689413 | 1.23E-06 |
| TMEM121 | -1.3049247 | 1.24E-06 |
| PPP6R2 | -1.7819389 | 1.26E-06 |
| OXR1 | 2.10791003 | 1.26E-06 |
| ARHGAP45 | -1.787733 | 1.26E-06 |

| | | |
|---|---|---|
| ATP1B3 | 10.2129116 | 1.26E-06 |
| TAF1D | 1.18453211 | 1.27E-06 |
| CNN1 | -5.1047287 | 1.28E-06 |
| KDM5B | 1.35852833 | 1.30E-06 |
| ZNF750 | 1.14304399 | 1.30E-06 |
| NAA38 | -4.3273704 | 1.30E-06 |
| RARRES1 | 36.8267544 | 1.30E-06 |
| CSNK1D | -1.5789398 | 1.31E-06 |
| HNRNPA2B1 | 5.37330074 | 1.31E-06 |
| NDUFAF3 | -4.620704 | 1.34E-06 |
| ARHGAP1 | -3.3410884 | 1.35E-06 |
| RBM6 | -1.507267 | 1.37E-06 |
| LMX1B | -3.5072646 | 1.39E-06 |
| FBXO46 | -1.5154003 | 1.40E-06 |
| SMG1 | 1.11461491 | 1.41E-06 |
| ELMO2 | 1.34961369 | 1.43E-06 |
| MED25 | -1.1849418 | 1.43E-06 |
| IKBKB | -2.33774 | 1.43E-06 |
| PRR14 | -1.8773985 | 1.43E-06 |
| C9orf16 | -5.0415347 | 1.43E-06 |
| ANKRD52 | 1.33267422 | 1.46E-06 |
| SSH3 | -4.9776757 | 1.47E-06 |
| PDPN | -1.8678132 | 1.47E-06 |
| JOSD2 | -3.8912275 | 1.48E-06 |
| RPL22 | -4.3059879 | 1.49E-06 |
| TRUB1 | 1.4345591 | 1.50E-06 |
| C9orf116 | -1.5288657 | 1.51E-06 |
| ARFRP1 | -1.2328117 | 1.51E-06 |
| FHL1 | -3.6920781 | 1.52E-06 |

| | | |
|---|---|---|
| EIF3A | 5.20259459 | 1.52E-06 |
| RPS5 | -30.948661 | 1.54E-06 |
| CACFD1 | -2.9570744 | 1.55E-06 |
| ACTA2 | -13.501802 | 1.56E-06 |
| TPRKB | 1.65003048 | 1.56E-06 |
| MBD6 | -1.8439501 | 1.59E-06 |
| ABHD10 | 1.63459671 | 1.61E-06 |
| CRACR2B | -5.9349149 | 1.62E-06 |
| CDK10 | -1.1349905 | 1.68E-06 |
| POLR3GL | -1.0614656 | 1.68E-06 |
| BID | 1.03769136 | 1.69E-06 |
| ASPN | -22.62596 | 1.71E-06 |
| BATF2 | 1.56607856 | 1.72E-06 |
| TMEM87A | 2.68249267 | 1.75E-06 |
| COPG1 | 8.14483606 | 1.80E-06 |
| CERS4 | -5.7802897 | 1.81E-06 |
| CTPS2 | 2.59610416 | 1.83E-06 |
| TOP2A | 13.1217333 | 1.83E-06 |
| DIPK2A | 1.68700676 | 1.84E-06 |
| AXL | -2.0882264 | 1.85E-06 |
| CCNE1 | 3.04685324 | 1.88E-06 |
| CHAD | -11.151877 | 1.90E-06 |
| PAQR4 | 1.69996928 | 1.91E-06 |
| MRAS | 2.02135817 | 1.91E-06 |
| ARRDC2 | -1.4504818 | 1.92E-06 |
| PEX16 | -1.5969277 | 1.97E-06 |
| ATF6 | 2.1166093 | 1.99E-06 |
| RERE | -1.4606139 | 2.01E-06 |
| FZD6 | 3.97495502 | 2.03E-06 |

| | | |
|---|---|---|
| TLE2 | -2.4111988 | 2.03E-06 |
| C5AR2 | -1.0084667 | 2.03E-06 |
| DOCK1 | -1.7413818 | 2.06E-06 |
| MLF1 | 1.70367024 | 2.06E-06 |
| TPGS2 | 1.11121181 | 2.07E-06 |
| LRRC15 | -9.2859119 | 2.07E-06 |
| ACADS | -2.3138017 | 2.07E-06 |
| SEPTIN3 | 1.40589831 | 2.09E-06 |
| GNE | 1.7321236 | 2.09E-06 |
| RIT1 | 1.42337175 | 2.09E-06 |
| GLI3 | -1.7670735 | 2.09E-06 |
| SLC16A5 | -1.237563 | 2.11E-06 |
| VPS41 | 1.14124424 | 2.14E-06 |
| SLAIN2 | 1.43800905 | 2.16E-06 |
| ZNFX1 | 2.58902527 | 2.16E-06 |
| USP11 | -2.2757368 | 2.23E-06 |
| PROM1 | 6.55580956 | 2.24E-06 |
| RABEP2 | -2.1030297 | 2.24E-06 |
| AADAT | 1.07330459 | 2.25E-06 |
| PDCD6IP | 2.41526312 | 2.25E-06 |
| TRIM2 | 1.02265785 | 2.26E-06 |
| TRPM4 | -2.3001224 | 2.27E-06 |
| FBXW2 | 1.06036647 | 2.27E-06 |
| SPPL2A | 1.37930547 | 2.34E-06 |
| PARP14 | 3.83304514 | 2.34E-06 |
| SYAP1 | 5.78977448 | 2.35E-06 |
| PDK4 | -6.6506734 | 2.40E-06 |
| SEPHS2 | 13.7977169 | 2.46E-06 |
| MED8 | 1.49398351 | 2.51E-06 |

| | | |
|---|---|---|
| SLC4A11 | 1.43203002 | 2.51E-06 |
| WAPL | 1.69916449 | 2.54E-06 |
| P4HB | 25.4586113 | 2.56E-06 |
| UBR4 | 1.15308002 | 2.56E-06 |
| BCL2 | -2.7474321 | 2.60E-06 |
| TMEM160 | -2.4471844 | 2.60E-06 |
| NDUFB5 | 1.69065889 | 2.61E-06 |
| FAM168B | 2.92361341 | 2.67E-06 |
| NAB2 | -2.218113 | 2.72E-06 |
| RASSF4 | 1.28365879 | 2.74E-06 |
| ST6GAL1 | 3.33743906 | 2.75E-06 |
| ATF7IP | 1.40155318 | 2.79E-06 |
| PLEKHA4 | -2.7195712 | 2.81E-06 |
| TOMM34 | 4.34989677 | 2.82E-06 |
| LARGE2 | 3.08512485 | 2.86E-06 |
| PPM1G | 5.04536551 | 2.88E-06 |
| GBP5 | 2.47753099 | 2.89E-06 |
| REEP6 | -13.040715 | 2.90E-06 |
| ZBTB41 | 1.99429348 | 2.96E-06 |
| CHRAC1 | 2.16151768 | 3.00E-06 |
| MRPS16 | 4.27880318 | 3.00E-06 |
| TIFA | 1.17265587 | 3.04E-06 |
| PLIN1 | -5.8605005 | 3.08E-06 |
| HDAC11 | -2.4529361 | 3.11E-06 |
| UBE2E3 | 3.0476657 | 3.18E-06 |
| NNAT | -1.0918651 | 3.20E-06 |
| HSPA2 | -4.5520199 | 3.22E-06 |
| SLC39A7 | 12.3042872 | 3.22E-06 |
| MFNG | -1.0135842 | 3.23E-06 |

| | | |
|---|---|---|
| KHDRBS1 | 4.20474135 | 3.23E-06 |
| PDXK | 2.92636754 | 3.24E-06 |
| PGGHG | -10.261482 | 3.27E-06 |
| CDKN2A | 3.43713527 | 3.28E-06 |
| TP53RK | 1.49942296 | 3.29E-06 |
| PCYOX1 | 3.84062949 | 3.35E-06 |
| CBR3 | 1.30488797 | 3.47E-06 |
| DRAP1 | -11.795067 | 3.51E-06 |
| CDH3 | 6.695139 | 3.52E-06 |
| CCN2 | -64.853399 | 3.52E-06 |
| RPRD1A | 1.4466151 | 3.52E-06 |
| SENP6 | 1.04487523 | 3.53E-06 |
| SERPINE1 | -12.641787 | 3.58E-06 |
| PPIF | 13.0716196 | 3.64E-06 |
| AFTPH | 2.17920359 | 3.69E-06 |
| ELF4 | 1.33156083 | 3.70E-06 |
| FOSB | -10.360979 | 3.70E-06 |
| USP14 | 4.46888412 | 3.70E-06 |
| MLXIP | 1.17537246 | 3.72E-06 |
| KLF16 | -1.0387506 | 3.76E-06 |
| FAM20B | 2.90374535 | 3.76E-06 |
| RPL24 | -31.449816 | 3.80E-06 |
| NAT14 | -2.9999181 | 3.84E-06 |
| RNF20 | 1.7337531 | 3.85E-06 |
| PSIP1 | 2.49600935 | 3.87E-06 |
| MAX | -1.1350994 | 3.98E-06 |
| WDR6 | -3.7949679 | 3.99E-06 |
| YTHDF1 | 4.01732299 | 4.06E-06 |
| DDX60 | 3.41413774 | 4.08E-06 |

| | | |
|---|---|---|
| ALDH9A1 | 6.42271931 | 4.10E-06 |
| SNX14 | 1.20703138 | 4.25E-06 |
| BPGM | 1.43936863 | 4.32E-06 |
| PON2 | 3.10162203 | 4.33E-06 |
| RBM38 | 3.95698781 | 4.36E-06 |
| DTX3L | 3.64643702 | 4.37E-06 |
| SLFN11 | 1.29019362 | 4.38E-06 |
| SCAND1 | -7.0009901 | 4.42E-06 |
| SLC9A7 | 1.09653271 | 4.44E-06 |
| CD81 | -9.3170899 | 4.44E-06 |
| GGT7 | -1.8426682 | 4.47E-06 |
| DEGS1 | 18.9787099 | 4.56E-06 |
| DCAF7 | 4.19535285 | 4.59E-06 |
| PDE4B | 2.28716015 | 4.63E-06 |
| SYNE4 | -1.5964414 | 4.73E-06 |
| TGIF1 | -1.2425122 | 4.73E-06 |
| HMGXB3 | -1.4216755 | 4.74E-06 |
| PYCARD | -7.2569939 | 4.86E-06 |
| DHCR7 | 7.8772394 | 4.87E-06 |
| DSG2 | 6.23937112 | 4.92E-06 |
| ELK4 | 1.06736818 | 4.98E-06 |
| TMEM214 | 2.9593721 | 5.00E-06 |
| PARP9 | 2.90265711 | 5.00E-06 |
| SNX5 | 1.62826822 | 5.16E-06 |
| PRPF3 | 2.29024408 | 5.17E-06 |
| APPL1 | 1.44640504 | 5.22E-06 |
| ATG4B | -1.0286804 | 5.47E-06 |
| GABRP | 29.3518306 | 5.53E-06 |
| LMNA | -11.1417 | 5.55E-06 |

| | | |
|---|---|---|
| LRRCC1 | 2.31282386 | 5.58E-06 |
| SPDEF | -25.340358 | 5.59E-06 |
| PLPPR2 | -2.305979 | 5.60E-06 |
| TLR2 | 1.03401149 | 5.61E-06 |
| PRDX6 | 14.4188548 | 5.64E-06 |
| CYBB | 5.40447761 | 5.66E-06 |
| SOAT1 | 3.01782922 | 5.66E-06 |
| SCD | 62.4016301 | 5.73E-06 |
| DHX29 | 2.07796481 | 5.77E-06 |
| SNX7 | 1.90143828 | 5.77E-06 |
| SPC24 | 1.14076578 | 5.81E-06 |
| ELK3 | -1.9859253 | 5.82E-06 |
| FAR2 | 1.32233111 | 5.83E-06 |
| A4GALT | -1.6959512 | 5.85E-06 |
| TIMP3 | -54.534441 | 5.87E-06 |
| NONO | 3.61703889 | 5.94E-06 |
| HBP1 | 1.23685631 | 5.97E-06 |
| HCFC1 | 1.35911409 | 5.97E-06 |
| MAGEA4 | 3.33715653 | 6.00E-06 |
| PREPL | 1.10628852 | 6.03E-06 |
| UTP18 | 5.47910941 | 6.04E-06 |
| SLC25A3 | 3.68569333 | 6.07E-06 |
| AP3B1 | 1.48690011 | 6.11E-06 |
| YBX1 | 25.9041614 | 6.19E-06 |
| MTA1 | -1.0382887 | 6.28E-06 |
| MTCH1 | 8.80271806 | 6.39E-06 |
| DBI | 18.2831197 | 6.40E-06 |
| ZNF692 | -2.0490992 | 6.44E-06 |
| KLHDC7B | 6.7151059 | 6.48E-06 |

| | | |
|---|---|---|
| POC1A | 1.18259782 | 6.48E-06 |
| ZMYND11 | 3.16526458 | 6.52E-06 |
| WDR54 | -1.1894941 | 6.59E-06 |
| RAB3GAP1 | 1.28430666 | 6.60E-06 |
| RNF149 | 1.12629278 | 6.62E-06 |
| ATP5IF1 | -5.9795642 | 6.62E-06 |
| NR1D1 | -2.1850415 | 6.67E-06 |
| TMEM126B | 1.55563531 | 6.78E-06 |
| CMTR1 | 1.41069017 | 6.79E-06 |
| FABP4 | -32.156462 | 6.79E-06 |
| GATD1 | -1.7345456 | 6.81E-06 |
| DDX50 | 1.58254031 | 6.84E-06 |
| SUMO1 | 5.39659113 | 6.90E-06 |
| NT5DC2 | 2.96133863 | 6.90E-06 |
| BMP1 | -1.6543823 | 6.90E-06 |
| AP1S2 | 1.26348999 | 6.90E-06 |
| KCNN4 | 5.58109539 | 6.93E-06 |
| TP53I13 | -1.8376304 | 7.00E-06 |
| TMEM37 | -1.4517368 | 7.02E-06 |
| ZRSR2 | -1.0847448 | 7.03E-06 |
| VPS29 | 1.6897649 | 7.04E-06 |
| RUNX1 | -2.5933052 | 7.07E-06 |
| PREB | 2.38891774 | 7.08E-06 |
| NUP210 | 3.89252359 | 7.12E-06 |
| TSPAN15 | -10.950943 | 7.19E-06 |
| CDH1 | 20.7160615 | 7.42E-06 |
| CCDC115 | -1.160107 | 7.65E-06 |
| SNRPB2 | 5.6188665 | 7.70E-06 |
| UBE2K | 2.75303723 | 7.70E-06 |

| | | |
|---|---|---|
| DEDD | 1.71297061 | 7.73E-06 |
| WDFY1 | 1.0648137 | 7.82E-06 |
| SLC34A2 | 8.50716116 | 7.87E-06 |
| SNX3 | 12.3897828 | 7.89E-06 |
| GSDMC | 1.55877551 | 7.89E-06 |
| MRPL41 | -15.147651 | 7.96E-06 |
| COA3 | -5.1218059 | 7.99E-06 |
| NXF1 | -1.4420542 | 8.02E-06 |
| RELL1 | -2.4490457 | 8.10E-06 |
| BAG3 | -8.7831543 | 8.18E-06 |
| AHCYL2 | 1.00857639 | 8.21E-06 |
| YAP1 | 2.39909008 | 8.21E-06 |
| FOXK2 | 1.22996526 | 8.21E-06 |
| EIF1 | -16.115487 | 8.22E-06 |
| RTP4 | 3.61353525 | 8.23E-06 |
| MARCHF7 | 1.71727516 | 8.24E-06 |
| PMVK | -13.194197 | 8.28E-06 |
| CD2BP2 | -3.5438692 | 8.57E-06 |
| NPLOC4 | 1.93566995 | 8.59E-06 |
| PRR12 | -1.2757996 | 8.60E-06 |
| SNX10 | 1.41989236 | 8.63E-06 |
| PDIA3 | 21.8413909 | 8.63E-06 |
| IRX3 | -6.0174216 | 8.66E-06 |
| UBL7 | -2.797341 | 8.68E-06 |
| AK2 | 1.77416437 | 8.72E-06 |
| PRPF31 | -2.4591682 | 8.94E-06 |
| APOLD1 | -1.4731824 | 9.06E-06 |
| RPS2 | -77.492532 | 9.06E-06 |
| RETREG3 | -2.7840624 | 9.06E-06 |

| | | |
|---|---|---|
| HSPA4 | 5.11132624 | 9.19E-06 |
| TKFC | -1.1597364 | 9.25E-06 |
| DAG1 | 3.92304476 | 9.27E-06 |
| TMEM268 | 1.85490688 | 9.29E-06 |
| MED20 | 2.16100047 | 9.29E-06 |
| HIGD2A | -9.3876799 | 9.31E-06 |
| G6PD | 6.09057377 | 9.43E-06 |
| SCAF1 | -3.0521769 | 9.47E-06 |
| PSD4 | -1.0406732 | 9.52E-06 |
| CTSL | 3.4601372 | 9.62E-06 |
| MOCOS | 1.11932921 | 9.63E-06 |
| EXT1 | 1.24386955 | 9.67E-06 |
| RPS3A | -17.062779 | 9.69E-06 |
| WASHC5 | 4.43705325 | 9.80E-06 |
| PEX5 | 1.49363907 | 9.88E-06 |
| TRIM44 | 1.78332538 | 9.92E-06 |
| GALNT10 | -2.44136 | 9.93E-06 |
| PDCL3 | 2.26373763 | 9.95E-06 |
| GSR | 5.51481254 | 1.02E-05 |
| UBE2L6 | 14.1219747 | 1.04E-05 |
| TRPT1 | -1.5601394 | 1.05E-05 |
| BRAT1 | -1.5363134 | 1.05E-05 |
| BCAR3 | -1.5756348 | 1.05E-05 |
| JAG2 | -1.1031084 | 1.06E-05 |
| A2ML1 | 2.89778539 | 1.06E-05 |
| WDTC1 | -1.888802 | 1.06E-05 |
| EIF3E | 1.63043077 | 1.07E-05 |
| CPNE3 | 7.01468787 | 1.07E-05 |
| KLHL24 | 1.21312484 | 1.07E-05 |

| | | |
|---|---|---|
| MTLN | -1.187091 | 1.07E-05 |
| ZNHIT2 | -1.9356786 | 1.07E-05 |
| BUD23 | 1.57199891 | 1.07E-05 |
| ELAC2 | 1.02841809 | 1.08E-05 |
| SLK | 1.6604287 | 1.09E-05 |
| CACNG4 | -10.193409 | 1.11E-05 |
| URM1 | -1.4284326 | 1.12E-05 |
| EEFSEC | -1.1419095 | 1.13E-05 |
| RPLP2 | -80.684943 | 1.13E-05 |
| HSF4 | -1.0042514 | 1.13E-05 |
| NDUFB7 | -26.529794 | 1.14E-05 |
| MAPK3 | -3.6561097 | 1.14E-05 |
| WRNIP1 | 2.21850052 | 1.14E-05 |
| FUBP1 | 1.68753121 | 1.14E-05 |
| G6PC3 | -3.3832819 | 1.14E-05 |
| RPL36 | -42.300593 | 1.15E-05 |
| ZNF623 | 1.55397343 | 1.15E-05 |
| IPO13 | 1.02697347 | 1.15E-05 |
| TBRG4 | 1.63179318 | 1.15E-05 |
| DOHH | -1.1734318 | 1.15E-05 |
| NSDHL | 2.33275378 | 1.16E-05 |
| TGIF2 | 2.09820683 | 1.17E-05 |
| RCOR1 | 1.54314019 | 1.19E-05 |
| RAB11FIP3 | -2.0948855 | 1.19E-05 |
| C1QTNF3 | -1.3897259 | 1.19E-05 |
| TMEM30A | 4.61542503 | 1.20E-05 |
| OMD | -2.1622918 | 1.20E-05 |
| TLNRD1 | -1.1430561 | 1.20E-05 |
| LUM | -75.040293 | 1.20E-05 |

| | | |
|---|---|---|
| COPS2 | 1.13118935 | 1.20E-05 |
| ECI1 | -4.3431868 | 1.20E-05 |
| STK32B | -1.3964394 | 1.20E-05 |
| AFF3 | -3.8206925 | 1.21E-05 |
| CARM1 | 1.38361301 | 1.22E-05 |
| CKAP4 | 6.9549105 | 1.23E-05 |
| TMEM126A | 2.24705745 | 1.23E-05 |
| TIMM13 | -6.5404446 | 1.24E-05 |
| CNIH1 | 1.9323059 | 1.25E-05 |
| CFAP97 | 1.17227193 | 1.25E-05 |
| THAP4 | -2.2415223 | 1.27E-05 |
| IGFBP2 | -15.376819 | 1.28E-05 |
| C19orf53 | -10.838963 | 1.28E-05 |
| TSPYL5 | 4.8645725 | 1.29E-05 |
| LIN7C | 1.09218281 | 1.30E-05 |
| VAPB | 1.89464869 | 1.31E-05 |
| ZNF217 | 4.86964524 | 1.32E-05 |
| MAP2K2 | -2.9126448 | 1.32E-05 |
| ACAD9 | 1.03015952 | 1.33E-05 |
| EPSTI1 | 1.43863682 | 1.37E-05 |
| SIL1 | -2.767212 | 1.39E-05 |
| ZMYND10 | -1.7859833 | 1.44E-05 |
| EIF2A | 1.78910415 | 1.44E-05 |
| EDEM3 | 2.84769294 | 1.44E-05 |
| DDB1 | 2.79422818 | 1.45E-05 |
| TMEM45A | 3.30803515 | 1.45E-05 |
| RAP1A | 1.1971452 | 1.47E-05 |
| CD163 | 3.7245365 | 1.47E-05 |
| ODF3B | -2.6119559 | 1.48E-05 |

| | | |
|---|---|---|
| RTN3 | 7.20818969 | 1.49E-05 |
| IRF3 | -2.2962607 | 1.50E-05 |
| TAOK2 | -1.2061144 | 1.50E-05 |
| ENO1 | 39.8029932 | 1.50E-05 |
| GRHL2 | 2.33918251 | 1.51E-05 |
| ITGB1 | 4.25566803 | 1.52E-05 |
| BCAR1 | -1.0926242 | 1.54E-05 |
| CYP1B1 | 10.9694445 | 1.57E-05 |
| REXO2 | 1.12472206 | 1.59E-05 |
| SELP | -1.0556394 | 1.60E-05 |
| SLC25A24 | 1.74446858 | 1.61E-05 |
| WDR26 | 2.08198923 | 1.62E-05 |
| DGCR2 | -2.8357422 | 1.62E-05 |
| PYCR1 | 5.36717607 | 1.65E-05 |
| HNRNPK | 7.65234211 | 1.67E-05 |
| CENPB | -5.5736003 | 1.68E-05 |
| GLIPR2 | 4.78161554 | 1.69E-05 |
| B4GALT3 | 3.47850353 | 1.69E-05 |
| COX7C | -12.405083 | 1.70E-05 |
| MX1 | 9.91866434 | 1.70E-05 |
| PLEKHB2 | 2.56623172 | 1.71E-05 |
| LPAR6 | -1.2362914 | 1.72E-05 |
| WBP11 | 6.50941663 | 1.73E-05 |
| TMEM14B | 1.1839484 | 1.74E-05 |
| SELENOW | -5.9793022 | 1.74E-05 |
| SLC37A1 | 1.05009968 | 1.76E-05 |
| ADI1 | 2.00230384 | 1.77E-05 |
| TPD52L1 | 2.18316387 | 1.78E-05 |
| SAFB | -1.3254451 | 1.80E-05 |

| | | |
|---|---|---|
| ACKR3 | -5.7916743 | 1.83E-05 |
| YIPF5 | 1.59011434 | 1.84E-05 |
| TAGLN | -10.351693 | 1.85E-05 |
| NMU | 1.35430561 | 1.85E-05 |
| VCPIP1 | 1.11566912 | 1.85E-05 |
| NDRG1 | 11.8732516 | 1.86E-05 |
| WTAP | 1.20745717 | 1.87E-05 |
| USP25 | 1.14825409 | 1.88E-05 |
| APLNR | -2.5365442 | 1.88E-05 |
| SMCO4 | 3.1443138 | 1.91E-05 |
| PMF1 | -1.3805865 | 1.92E-05 |
| FIBP | -2.4068016 | 1.93E-05 |
| CX3CR1 | -1.3463142 | 1.97E-05 |
| NDRG3 | 2.63709522 | 2.01E-05 |
| NCEH1 | 1.72218416 | 2.02E-05 |
| RBCK1 | -2.5282845 | 2.03E-05 |
| FRMD6 | -2.0213469 | 2.03E-05 |
| SWI5 | -1.2556048 | 2.04E-05 |
| PCOLCE | -15.484565 | 2.04E-05 |
| IK | -2.4708528 | 2.05E-05 |
| VDAC2 | 3.48221307 | 2.06E-05 |
| NELFCD | 3.27572693 | 2.08E-05 |
| KIF3B | 2.48996573 | 2.10E-05 |
| ARL1 | 3.37795828 | 2.10E-05 |
| GLS | 2.4414876 | 2.10E-05 |
| METRNL | -2.9344326 | 2.10E-05 |
| TCEAL1 | -7.4816093 | 2.10E-05 |
| CIR1 | -1.3621392 | 2.13E-05 |
| CEBPB | 8.61520891 | 2.14E-05 |

| | | |
|---|---|---|
| GBA | 3.16131735 | 2.15E-05 |
| EN1 | 4.93601176 | 2.16E-05 |
| PPP1R35 | -1.4352443 | 2.19E-05 |
| GATAD2A | 1.41301056 | 2.20E-05 |
| CLPTM1L | 3.17405358 | 2.22E-05 |
| CORO6 | -3.2507089 | 2.22E-05 |
| FAM160B2 | -1.0725316 | 2.23E-05 |
| SLC9A3R2 | -8.7104318 | 2.25E-05 |
| FDXR | -1.3963338 | 2.25E-05 |
| ARHGAP18 | 1.30782238 | 2.26E-05 |
| UBE2E2 | 1.34425079 | 2.26E-05 |
| RSAD2 | 3.86977846 | 2.28E-05 |
| KIFAP3 | 1.35795249 | 2.31E-05 |
| TFAP2C | 5.74345239 | 2.33E-05 |
| PHKB | 1.43946332 | 2.35E-05 |
| SLC30A5 | 1.12486498 | 2.37E-05 |
| MFAP2 | -2.8991259 | 2.37E-05 |
| SAT2 | -2.9220443 | 2.39E-05 |
| USP33 | 1.42511236 | 2.40E-05 |
| C1orf43 | 13.847544 | 2.40E-05 |
| SRSF3 | 2.93890076 | 2.41E-05 |
| FSTL1 | -8.591456 | 2.44E-05 |
| MFSD10 | -4.6707941 | 2.47E-05 |
| CLTC | 21.1830072 | 2.48E-05 |
| DDX58 | 4.78865122 | 2.52E-05 |
| CDKN1C | -1.5457878 | 2.58E-05 |
| RABGAP1 | 1.14988258 | 2.58E-05 |
| RFX5 | 1.99715939 | 2.60E-05 |
| SPON2 | -3.4875137 | 2.66E-05 |

| | | |
|---|---|---|
| PYM1 | -1.7222355 | 2.70E-05 |
| ACSL1 | 5.25573884 | 2.71E-05 |
| GOLGA3 | 1.09173262 | 2.71E-05 |
| LYZ | 48.9960926 | 2.71E-05 |
| CDC6 | 3.59572634 | 2.74E-05 |
| CXCL11 | 5.70659849 | 2.74E-05 |
| HPS1 | -1.2406622 | 2.74E-05 |
| LRP6 | 1.56064084 | 2.75E-05 |
| CYSTM1 | -2.6420838 | 2.78E-05 |
| ANO1 | -10.188254 | 2.79E-05 |
| RAB5C | -2.239665 | 2.79E-05 |
| GRAMD1A | -2.3716672 | 2.82E-05 |
| TNIP1 | -2.3276337 | 2.83E-05 |
| NIPSNAP1 | 3.72071552 | 2.88E-05 |
| TNKS2 | 1.8273068 | 2.89E-05 |
| PIAS4 | -1.1411448 | 2.89E-05 |
| PPP1R13L | -1.8087419 | 2.90E-05 |
| COL17A1 | -4.7914783 | 2.91E-05 |
| ADAM12 | -3.065668 | 2.98E-05 |
| PDIA5 | 1.43978708 | 3.00E-05 |
| HERC6 | 2.52024247 | 3.00E-05 |
| LCN2 | 14.4321445 | 3.01E-05 |
| PIGO | 1.14519999 | 3.04E-05 |
| CDH11 | -2.3850647 | 3.05E-05 |
| CEACAM1 | 2.27199488 | 3.06E-05 |
| DDX27 | 1.61061865 | 3.09E-05 |
| HAGH | -1.3203621 | 3.19E-05 |
| PPP1R2 | 1.75258682 | 3.20E-05 |
| CBX1 | 8.7252913 | 3.21E-05 |

| | | |
|---|---|---|
| SERPINB6 | -1.3369494 | 3.22E-05 |
| THNSL2 | -1.2523448 | 3.24E-05 |
| RNF11 | 3.88850082 | 3.24E-05 |
| SLC43A3 | 1.52735934 | 3.34E-05 |
| NUDC | -5.527941 | 3.36E-05 |
| ANO10 | 1.03997774 | 3.40E-05 |
| POLR1E | 2.08942065 | 3.41E-05 |
| NIPBL | 1.2397962 | 3.43E-05 |
| NENF | -13.694753 | 3.46E-05 |
| MAL2 | 21.6943216 | 3.47E-05 |
| MSMO1 | 5.07481731 | 3.52E-05 |
| HSCB | -1.000106 | 3.58E-05 |
| CDR2 | 1.60781034 | 3.58E-05 |
| ZNF581 | -2.6076817 | 3.59E-05 |
| EIF5 | 2.82787797 | 3.59E-05 |
| NUBP2 | -2.25442 | 3.60E-05 |
| PDZK1IP1 | 12.7791721 | 3.60E-05 |
| NAB1 | 1.06317814 | 3.66E-05 |
| ZNF467 | -1.812541 | 3.80E-05 |
| APOBEC3B | 2.10023638 | 3.80E-05 |
| KAT8 | -1.1519837 | 3.81E-05 |
| FAM174A | -3.2273867 | 3.86E-05 |
| SMPDL3A | 1.34221271 | 3.92E-05 |
| DOLPP1 | 1.253281 | 3.92E-05 |
| CLIC4 | 9.57955192 | 3.94E-05 |
| URI1 | 4.97900138 | 3.94E-05 |
| C1orf54 | -1.0372873 | 3.96E-05 |
| PLIN2 | 3.79541026 | 3.98E-05 |
| LSM7 | -3.9027099 | 3.99E-05 |

| | | |
|---|---|---|
| ATAD1 | 1.32553615 | 4.01E-05 |
| ZNF106 | 1.03182252 | 4.01E-05 |
| SPRYD3 | -2.7063769 | 4.02E-05 |
| EXT2 | 1.15566667 | 4.03E-05 |
| SUMO3 | 5.90060515 | 4.04E-05 |
| CHI3L2 | 10.9309063 | 4.06E-05 |
| ABCD1 | 1.71687388 | 4.07E-05 |
| HDLBP | 6.03549872 | 4.10E-05 |
| UBR5 | 2.64688426 | 4.13E-05 |
| TP63 | -1.0315204 | 4.14E-05 |
| NCDN | -2.260671 | 4.14E-05 |
| CHMP5 | 4.51711867 | 4.15E-05 |
| C7orf50 | -2.5828979 | 4.16E-05 |
| ARSA | -2.1425768 | 4.19E-05 |
| ADRA2A | -2.182548 | 4.19E-05 |
| AGPAT3 | 1.09184864 | 4.19E-05 |
| RPRD1B | 2.13057055 | 4.20E-05 |
| RHOT1 | 1.51922673 | 4.20E-05 |
| PRPSAP1 | 1.13680094 | 4.24E-05 |
| TSR3 | -4.5588526 | 4.24E-05 |
| SGPP2 | 1.47270036 | 4.24E-05 |
| OSTF1 | 3.25271312 | 4.27E-05 |
| CYBRD1 | -21.004145 | 4.34E-05 |
| UBE2D3 | 2.13548346 | 4.36E-05 |
| H2BC11 | 1.38057878 | 4.39E-05 |
| DPYSL3 | -4.8892768 | 4.40E-05 |
| OAZ2 | -1.0624246 | 4.54E-05 |
| COG4 | 1.44354553 | 4.59E-05 |
| FBXW11 | 1.27276014 | 4.67E-05 |

| | | |
|---|---|---|
| STAB1 | -1.3757334 | 4.67E-05 |
| BBX | 1.15116715 | 4.68E-05 |
| MBOAT2 | 3.25915675 | 4.70E-05 |
| EGFL7 | -3.8884869 | 4.76E-05 |
| CHKA | 1.28623077 | 4.77E-05 |
| TRAK1 | -1.2201469 | 4.82E-05 |
| C1orf21 | -2.6501763 | 4.85E-05 |
| TALDO1 | 8.38154178 | 4.86E-05 |
| TP53I11 | -4.2566199 | 4.95E-05 |
| ELF5 | 6.96396974 | 4.95E-05 |
| NAAA | 1.20195693 | 5.00E-05 |
| CYB5R3 | -3.6189602 | 5.01E-05 |
| CAP1 | 10.4129549 | 5.03E-05 |
| PRTFDC1 | 1.26913142 | 5.03E-05 |
| PLOD3 | 3.02488252 | 5.03E-05 |
| TNNT1 | -7.8553586 | 5.04E-05 |
| SEC31A | 2.68849511 | 5.05E-05 |
| PWWP2B | -2.1617674 | 5.08E-05 |
| ABCC1 | 1.22417334 | 5.09E-05 |
| APEX2 | 1.78133157 | 5.11E-05 |
| KMT2C | 1.36174248 | 5.11E-05 |
| EIF2B1 | 1.3807981 | 5.19E-05 |
| PBDC1 | 2.71164898 | 5.23E-05 |
| HNRNPL | 1.51357935 | 5.27E-05 |
| CCDC124 | -5.4604288 | 5.31E-05 |
| PKP2 | 1.03832004 | 5.31E-05 |
| DNAJA2 | 2.53498294 | 5.32E-05 |
| ZNHIT6 | 1.23612192 | 5.35E-05 |
| TTYH3 | 3.99405118 | 5.42E-05 |

| | | |
|---|---|---|
| POFUT1 | 1.7430206 | 5.47E-05 |
| LMNB2 | 2.7084531 | 5.51E-05 |
| IMPDH2 | -5.7596744 | 5.53E-05 |
| UEVLD | 1.06173112 | 5.66E-05 |
| DENND4C | 1.10037485 | 5.69E-05 |
| BRPF3 | 1.48649271 | 5.70E-05 |
| MET | 1.47085919 | 5.71E-05 |
| ICAM1 | 4.18625994 | 5.73E-05 |
| GSTA4 | 2.78851836 | 5.78E-05 |
| COPG2 | 1.81303002 | 5.80E-05 |
| ARL4D | -1.5929594 | 5.86E-05 |
| ARFGEF1 | 2.74290303 | 5.86E-05 |
| SLC25A28 | -1.9849309 | 5.93E-05 |
| SRRM1 | 1.11953529 | 5.94E-05 |
| FAF2 | 1.59481829 | 5.99E-05 |
| ALG2 | 1.41691725 | 6.09E-05 |
| SLC6A8 | 2.36256571 | 6.17E-05 |
| THUMPD1 | 1.66081603 | 6.22E-05 |
| IQGAP1 | 4.87700345 | 6.24E-05 |
| UCHL1 | 6.67611979 | 6.24E-05 |
| COX7B | 3.58318538 | 6.26E-05 |
| TOMM22 | 3.49167831 | 6.38E-05 |
| FAT1 | 3.12181929 | 6.43E-05 |
| VEZT | 1.18083611 | 6.51E-05 |
| CYP7B1 | 1.04522313 | 6.52E-05 |
| LFNG | -5.6035204 | 6.58E-05 |
| ELOC | 1.97752296 | 6.68E-05 |
| HSPB1 | -122.01475 | 6.70E-05 |
| ARHGEF11 | 1.32131226 | 6.73E-05 |

| | | |
|---|---|---|
| ARFGEF3 | 2.39296639 | 6.82E-05 |
| GOLGA5 | 2.53503859 | 6.94E-05 |
| LGMN | 5.27412659 | 7.02E-05 |
| COLGALT1 | 4.04202454 | 7.03E-05 |
| HSDL2 | 3.07356691 | 7.07E-05 |
| FBXO21 | 1.04274324 | 7.09E-05 |
| ERMP1 | 3.61815677 | 7.10E-05 |
| ECHDC1 | 1.45920231 | 7.11E-05 |
| LZTS3 | 1.09923232 | 7.12E-05 |
| OASL | 2.86333393 | 7.16E-05 |
| RASD1 | -6.2414316 | 7.19E-05 |
| BARX1 | 1.45819977 | 7.19E-05 |
| FAM102A | -4.6934862 | 7.21E-05 |
| PABPC1L | -1.3056595 | 7.29E-05 |
| HECTD1 | 1.88754799 | 7.36E-05 |
| KRT18 | -54.730564 | 7.39E-05 |
| GPR137 | -1.1687506 | 7.40E-05 |
| TLCD3A | 1.09525281 | 7.45E-05 |
| CYB5D2 | -2.4954343 | 7.45E-05 |
| FBLN2 | -9.588568 | 7.48E-05 |
| TRIR | -10.271661 | 7.49E-05 |
| NUCB1 | -7.8807591 | 7.55E-05 |
| NUDT16L1 | -3.3340934 | 7.56E-05 |
| ACSL4 | 1.31606822 | 7.60E-05 |
| GID8 | 3.76690141 | 7.63E-05 |
| LRRC1 | 1.18959327 | 7.67E-05 |
| PEMT | -1.2518037 | 7.69E-05 |
| ITM2B | -4.8259369 | 7.70E-05 |
| DIRAS1 | 1.31915201 | 7.73E-05 |

| | | |
|---|---|---|
| ARL8B | 4.14770744 | 7.75E-05 |
| ATP6V1C1 | 3.07393036 | 7.76E-05 |
| PABPC4 | 1.8742308 | 7.80E-05 |
| TMEM86A | 1.8451009 | 7.80E-05 |
| VAT1 | -8.4689949 | 7.81E-05 |
| AHCY | 7.15828356 | 7.82E-05 |
| PTPA | -4.2526555 | 7.84E-05 |
| MYH11 | -2.556956 | 7.97E-05 |
| DECR1 | 2.17614524 | 8.01E-05 |
| SLC2A1 | 5.53086281 | 8.12E-05 |
| MAGED1 | 8.38303402 | 8.12E-05 |
| PHRF1 | -1.1958929 | 8.13E-05 |
| ZMYM2 | 1.17193827 | 8.14E-05 |
| TK1 | 12.0260368 | 8.19E-05 |
| TXN | 28.7578414 | 8.21E-05 |
| RIDA | 2.80402963 | 8.25E-05 |
| P4HA1 | 6.67113602 | 8.40E-05 |
| SKA2 | 3.42025851 | 8.45E-05 |
| DBNDD1 | 2.38480161 | 8.47E-05 |
| AP1B1 | 2.54891533 | 8.51E-05 |
| MS4A15 | -1.0710477 | 8.54E-05 |
| UBL4A | 2.78854789 | 8.54E-05 |
| DEF6 | -1.7868313 | 8.62E-05 |
| MRPL28 | -3.3802835 | 8.64E-05 |
| RRM2B | 2.22543276 | 8.65E-05 |
| FBRS | -1.6224045 | 8.80E-05 |
| GSTP1 | 38.6885634 | 8.83E-05 |
| ITGA5 | -2.6840283 | 8.84E-05 |
| MYBL1 | 1.28696416 | 8.93E-05 |

| | | |
|---|---|---|
| CAMK2N1 | -7.6810485 | 8.98E-05 |
| SH3GL1 | -2.6910677 | 9.05E-05 |
| CSNK1G2 | -2.0632872 | 9.06E-05 |
| GLOD4 | 1.04188039 | 9.20E-05 |
| AGPS | 1.39518511 | 9.33E-05 |
| CISH | -2.4894214 | 9.34E-05 |
| NME4 | -6.1655289 | 9.35E-05 |
| GPATCH4 | 1.44205568 | 9.41E-05 |
| SSNA1 | -7.0795031 | 9.55E-05 |
| CDC37 | -5.1866372 | 9.55E-05 |
| THBS4 | -3.9635622 | 9.61E-05 |
| PPM1H | 2.22210841 | 9.72E-05 |
| MCRIP2 | -1.4563607 | 9.81E-05 |
| SOX9 | 7.02175211 | 9.83E-05 |
| NOP56 | 2.86100792 | 9.86E-05 |
| DYNLRB1 | -1.1841046 | 9.87E-05 |
| GADD45GIP1 | -5.4744714 | 9.97E-05 |
| PKN1 | -4.7610845 | 0.00010154 |
| HIPK1 | 1.71452294 | 0.00010168 |
| SBNO2 | -1.9306317 | 0.00010211 |
| RPS15A | -1.8154722 | 0.00010223 |
| FOXA1 | -13.32333 | 0.00010279 |
| ADIPOQ | -3.4245362 | 0.00010339 |
| AGPAT2 | -6.3062039 | 0.00010383 |
| PHB | 4.17983232 | 0.00010386 |
| ECSIT | -2.0126371 | 0.00010386 |
| SDF4 | -3.4717737 | 0.00010426 |
| MIPEP | 1.70725525 | 0.00010494 |
| VWA1 | -4.3360109 | 0.00010568 |

| | | |
|---|---|---|
| SLFN5 | 1.88908489 | 0.00010673 |
| RPL27 | -32.38301 | 0.00010704 |
| TRIM37 | 2.37645854 | 0.00010935 |
| CCN4 | -1.645905 | 0.00011025 |
| CXCL5 | 1.2054241 | 0.00011195 |
| HCCS | 2.69015643 | 0.00011305 |
| RNF126 | -1.1485456 | 0.00011305 |
| LY6E | 52.1818289 | 0.00011448 |
| CD93 | -2.7426915 | 0.00011636 |
| MTREX | 1.5394831 | 0.00011816 |
| NECTIN4 | 5.35601248 | 0.00011823 |
| SMAD7 | -1.045801 | 0.00011851 |
| AC034236.1 | -3.7344391 | 0.00011904 |
| NDUFAB1 | 4.42768564 | 0.00011953 |
| ANKRD10 | -2.8017858 | 0.0001204 |
| GPR108 | -1.8782783 | 0.00012151 |
| SLC35B1 | 2.67970312 | 0.00012168 |
| POSTN | -56.895489 | 0.00012174 |
| XBP1 | -134.52062 | 0.00012197 |
| MDC1 | 1.48620427 | 0.00012228 |
| SLAMF7 | 1.91722138 | 0.00012255 |
| PDGFB | -2.4636763 | 0.00012278 |
| OGFR | -2.0620274 | 0.0001232 |
| DDX23 | 2.26607569 | 0.00012364 |
| LIMCH1 | 1.68144287 | 0.00012431 |
| RFXANK | -2.0224176 | 0.00012469 |
| BAP1 | -1.432653 | 0.00012504 |
| ACSF2 | -2.1604461 | 0.00012904 |
| FNBP1L | 2.90928691 | 0.00013018 |

| | | |
|---|---|---|
| PRUNE1 | 2.54296454 | 0.00013076 |
| FADS1 | 1.6583587 | 0.00013143 |
| SAYSD1 | 1.51070549 | 0.00013174 |
| ENTPD2 | 2.37472334 | 0.0001327 |
| FN3K | -1.1390408 | 0.00013299 |
| PHC2 | -2.2971574 | 0.00013383 |
| DHX32 | 1.15218684 | 0.00013397 |
| SCRN1 | 3.22073707 | 0.0001352 |
| PARK7 | -9.137554 | 0.00013617 |
| CDKN2C | 1.52520726 | 0.00013649 |
| SLC35E1 | 1.21539258 | 0.00013801 |
| TBC1D1 | 1.16789516 | 0.00013814 |
| SCARB1 | 1.97440678 | 0.0001385 |
| IFIT5 | 1.51877417 | 0.00013904 |
| TOMM20 | 12.3097723 | 0.00013955 |
| HMGN3 | 6.27771882 | 0.00014008 |
| MRFAP1 | -13.123906 | 0.00014012 |
| RPS8 | -49.765107 | 0.00014013 |
| ALDH16A1 | -1.086901 | 0.00014057 |
| DGKD | -1.047974 | 0.00014059 |
| CCL20 | 1.07782337 | 0.00014141 |
| CCND1 | -42.814675 | 0.00014151 |
| DNAJC10 | 1.18491939 | 0.0001419 |
| NUDT2 | -2.345349 | 0.00014205 |
| KLHDC9 | -1.6614791 | 0.00014259 |
| AURKAIP1 | -8.5922634 | 0.00014289 |
| TTC19 | 1.22901058 | 0.00014418 |
| ADAMTS2 | -2.3858283 | 0.00014563 |
| MGST2 | -1.4534577 | 0.00014582 |

| | | |
|---|---|---|
| IL27RA | 3.02862466 | 0.00014791 |
| KIFC2 | -1.3848306 | 0.00014865 |
| CCDC74A | -9.4363218 | 0.00014943 |
| CHST15 | -1.9788982 | 0.00014953 |
| SLC9A1 | -1.732695 | 0.00014969 |
| TTYH1 | 1.64927932 | 0.00015002 |
| NOB1 | 1.48688812 | 0.00015209 |
| DEDD2 | -1.3861724 | 0.00015343 |
| SMG8 | 1.62282014 | 0.00015501 |
| APH1A | 9.67952185 | 0.00015634 |
| POMP | 7.84530964 | 0.0001565 |
| CAPNS1 | -11.921209 | 0.00015703 |
| CERS6 | 2.92977645 | 0.00015742 |
| PRR15 | -5.7409768 | 0.00015765 |
| TMEM69 | 1.98200187 | 0.00015958 |
| BCCIP | 1.42184578 | 0.00015963 |
| CBR1 | 2.48316493 | 0.00015987 |
| N4BP3 | -1.6666554 | 0.00015995 |
| TGFA | 1.16443502 | 0.00016079 |
| SLC27A3 | -1.0847236 | 0.00016103 |
| ABTB2 | 1.04471962 | 0.00016402 |
| SGTA | -1.1259702 | 0.00016411 |
| ISYNA1 | -5.1778622 | 0.00016492 |
| CNPPD1 | -2.622215 | 0.000165 |
| GLYATL2 | 20.0563505 | 0.00016823 |
| LSM5 | 1.15024109 | 0.00016944 |
| AK5 | -1.1812743 | 0.00017016 |
| RCN3 | -10.781292 | 0.00017032 |
| RRNAD1 | -1.8298093 | 0.00017237 |

| | | |
|---|---|---|
| LY86 | -1.2418558 | 0.00017322 |
| FA2H | 1.15619076 | 0.00017343 |
| BORCS7 | -1.3077972 | 0.00017391 |
| TFPT | -2.7074526 | 0.00017517 |
| NUP85 | 1.22715104 | 0.00017563 |
| CRKL | 1.83991489 | 0.00017799 |
| DAP | 6.99535142 | 0.0001792 |
| MAP7 | 2.61747601 | 0.0001805 |
| SGPL1 | 2.26360464 | 0.00018311 |
| EPS8 | 1.10851431 | 0.00018395 |
| SUSD6 | -2.4415498 | 0.00018436 |
| TMEM245 | 1.07827774 | 0.00018553 |
| KLK6 | 5.69751433 | 0.00018648 |
| TESMIN | -2.0057912 | 0.00018793 |
| MARK2 | 1.00128923 | 0.00019098 |
| ATP5F1A | 4.15619856 | 0.00019312 |
| COL7A1 | -1.2584641 | 0.0001955 |
| ATP5ME | -2.3442116 | 0.00019732 |
| SAMD4B | 1.95589474 | 0.00019748 |
| SLC66A2 | -1.2653735 | 0.00019772 |
| SNRPC | 7.89934199 | 0.00019893 |
| SNRPD3 | 1.84236028 | 0.00019961 |
| DVL1 | -2.4266463 | 0.00019962 |
| MGME1 | 1.93001604 | 0.00020097 |
| TGFBR1 | 2.35129844 | 0.00020206 |
| NDUFB1 | -2.6265846 | 0.00020255 |
| RPL39L | 3.65516104 | 0.00020305 |
| ECD | 1.4016595 | 0.00020374 |
| DUSP12 | 1.16754295 | 0.00020401 |

| | | |
|---|---|---|
| CSTB | 16.8989103 | 0.00020533 |
| FRAT2 | 1.20638948 | 0.00020653 |
| SUCLG2 | 3.00880753 | 0.00020655 |
| ACADVL | -6.6317546 | 0.00020714 |
| NXPH3 | -1.0859298 | 0.00020725 |
| KDM5C | 1.65088511 | 0.0002074 |
| KIAA0100 | 3.18850167 | 0.00020935 |
| BRMS1 | -3.4921874 | 0.00021361 |
| RALGAPB | 2.00441349 | 0.00021434 |
| TECR | -3.0687726 | 0.000215 |
| IFI44L | 2.88559273 | 0.000215 |
| ENDOD1 | 2.98616212 | 0.00021607 |
| PRKD2 | -1.1232494 | 0.00021618 |
| SUN1 | 1.07734539 | 0.00022074 |
| GEMIN8 | -1.2004989 | 0.00022377 |
| VSIR | -1.2162677 | 0.00022601 |
| CTNNAL1 | 1.27602936 | 0.00022628 |
| CYP2J2 | 1.21590167 | 0.00022741 |
| FXR1 | 1.27297528 | 0.00022924 |
| B4GALT1 | 13.058732 | 0.00023022 |
| TYK2 | -1.1272634 | 0.00023059 |
| TPBG | -3.9704764 | 0.00023123 |
| SLC39A14 | 1.86447657 | 0.00023149 |
| NR1D2 | 1.38844968 | 0.00023234 |
| CDKAL1 | 1.58805053 | 0.00023471 |
| ULK3 | -1.4213082 | 0.00023533 |
| SLC3A2 | 5.64240103 | 0.00023549 |
| SIX3 | 1.1299243 | 0.0002357 |
| RASSF7 | -3.2579837 | 0.00023701 |

| | | |
|---|---|---|
| RHOD | -5.6073591 | 0.00023715 |
| JPH1 | 1.13200813 | 0.0002383 |
| WDR13 | -1.2255523 | 0.00023958 |
| ATRN | 1.32521003 | 0.00024135 |
| NPM1 | 7.71179315 | 0.0002415 |
| SNRPE | 4.62037571 | 0.0002444 |
| TCF7L1 | 2.34845871 | 0.00024492 |
| BSG | -19.977106 | 0.00024492 |
| IGSF3 | 2.47330229 | 0.00024569 |
| FGFR3 | -3.4935084 | 0.00024616 |
| LCP1 | 7.80773958 | 0.00024617 |
| PDGFD | -1.2975259 | 0.00024635 |
| GYPC | -1.2277899 | 0.00024635 |
| VIM | -19.717802 | 0.00024955 |
| DUSP23 | -8.7829579 | 0.00024967 |
| THOC6 | -2.4322108 | 0.00025129 |
| EFCC1 | -1.0236805 | 0.00025157 |
| JUP | -12.702714 | 0.00025428 |
| PCP2 | -2.1804779 | 0.00025428 |
| IDO1 | 5.23281533 | 0.00025528 |
| HADH | 1.05293725 | 0.00025665 |
| ARMCX2 | -2.3471344 | 0.00025685 |
| AHNAK | -12.458954 | 0.00025704 |
| MUC15 | 1.29737173 | 0.00025809 |
| TRIM3 | -1.0633065 | 0.00025809 |
| AGTR1 | -7.6939775 | 0.00026163 |
| CYTH3 | -1.1354664 | 0.00026471 |
| JADE3 | 1.1995011 | 0.00026668 |
| TMEM63B | 2.432925 | 0.00026668 |

| | | |
|---|---|---|
| UQCRFS1 | 5.03936235 | 0.00027 |
| CLDN12 | 1.2588059 | 0.00027054 |
| HM13 | 2.65272218 | 0.00027123 |
| AAGAB | 2.19013816 | 0.00027248 |
| FBXL16 | -2.4067903 | 0.00027412 |
| TGFBR2 | -2.9652204 | 0.00027761 |
| GZMB | 1.69094725 | 0.00027768 |
| LMAN2L | -1.4949209 | 0.0002789 |
| PSMA7 | 14.3989384 | 0.00028354 |
| RARG | -1.4270618 | 0.00028483 |
| LRPAP1 | -1.2960946 | 0.00028584 |
| ELK1 | 1.48141278 | 0.00028738 |
| VPS45 | 1.09777005 | 0.00029095 |
| EFHD1 | -23.694003 | 0.00029181 |
| CPXM2 | -1.9416234 | 0.00029359 |
| MOAP1 | -2.225971 | 0.00029374 |
| NECAB3 | -2.355628 | 0.00029545 |
| ZNHIT1 | -2.2889024 | 0.00029707 |
| ARHGAP12 | 1.36544972 | 0.00029833 |
| SAP30 | 1.75788991 | 0.00030424 |
| GBE1 | 1.00747256 | 0.00030586 |
| FAM174B | -2.7312308 | 0.00030762 |
| DRAM1 | 1.28468414 | 0.00030771 |
| WDR82 | 1.92013763 | 0.00030858 |
| PYCR2 | -1.7230343 | 0.00030991 |
| KIAA0930 | -1.3374722 | 0.00031031 |
| LINC01116 | -2.2284328 | 0.00031332 |
| EFNB1 | -1.7394634 | 0.00031532 |
| MTA2 | 2.64309764 | 0.00031705 |

| | | |
|---|---|---|
| IGSF21 | -2.1477043 | 0.00032192 |
| MKNK2 | -5.3784019 | 0.00032849 |
| MLEC | 4.11428245 | 0.00032901 |
| ZNF768 | -3.7347535 | 0.00032965 |
| TRMT112 | -12.501508 | 0.00033195 |
| TRIP10 | -1.2301572 | 0.00033399 |
| IL4I1 | 1.51372784 | 0.00033576 |
| TMX4 | 3.26860753 | 0.00033625 |
| AKIRIN2 | 1.5579415 | 0.00033762 |
| NDUFS4 | -2.8301098 | 0.00033802 |
| ROBO1 | 1.08145279 | 0.0003398 |
| NOTCH2 | 2.7945109 | 0.0003402 |
| SNRNP40 | 1.03722677 | 0.00034207 |
| RBL2 | 1.31504922 | 0.00034254 |
| VEGFA | 2.048619 | 0.00034575 |
| GXYLT2 | -1.53297 | 0.00034591 |
| MEST | 3.19269627 | 0.00034768 |
| SAR1A | 2.37272145 | 0.00034865 |
| GOT2 | 4.73046096 | 0.00035425 |
| PLBD1 | 3.47433494 | 0.00035507 |
| TOMM40 | 2.98127724 | 0.0003578 |
| CELSR1 | -3.5491364 | 0.00035877 |
| FAM122B | 1.35529667 | 0.00036187 |
| UBE2D2 | 1.90083198 | 0.00036352 |
| MICALL1 | 1.55082487 | 0.00036822 |
| TKT | 3.83629895 | 0.00037192 |
| TXLNA | 1.89733798 | 0.00037205 |
| GOPC | 1.04134311 | 0.00037846 |
| MSC | -1.3027714 | 0.00037996 |

| | | |
|---|---|---|
| RALBP1 | 1.4112636 | 0.00038004 |
| MAP4K4 | 1.62776777 | 0.00038191 |
| UNC5B | -2.3203273 | 0.00038321 |
| RARA | -7.1670406 | 0.00038446 |
| KLHDC3 | 8.5515791 | 0.00038515 |
| B3GAT3 | -2.8641677 | 0.00038939 |
| RPL26 | -1.8795732 | 0.00039167 |
| C1QTNF1 | -1.9451083 | 0.00039279 |
| CHIC2 | 1.11634696 | 0.0003973 |
| TUSC2 | -1.2986894 | 0.00039806 |
| RBP7 | -3.9269465 | 0.00040266 |
| SNX9 | -1.8279686 | 0.00040984 |
| FUCA2 | 2.42519645 | 0.00041197 |
| EGR2 | -2.0615841 | 0.00041216 |
| RPL4 | -26.290862 | 0.0004168 |
| SLC22A17 | -4.49992 | 0.00041897 |
| BIRC3 | 1.75639724 | 0.00041919 |
| PSME3IP1 | 1.47179022 | 0.00042265 |
| MLLT1 | -1.6577832 | 0.00042837 |
| OAS1 | 3.8157346 | 0.0004299 |
| EBNA1BP2 | 2.1400631 | 0.0004312 |
| UNC13D | 1.25221614 | 0.00043187 |
| ATP6V1B2 | 2.02284391 | 0.00043205 |
| HSPA13 | 2.13955849 | 0.00043826 |
| PFDN1 | -1.7465967 | 0.00044581 |
| HEBP2 | 1.49688524 | 0.00044719 |
| ITGB8 | 1.0557078 | 0.00045268 |
| LAG3 | 1.02845374 | 0.00045356 |
| CTHRC1 | -10.419765 | 0.0004553 |

| | | |
|---|---|---|
| CHD3 | -2.4777545 | 0.00045644 |
| RFC1 | 1.05937568 | 0.00046176 |
| NOVA1 | -1.5749007 | 0.0004618 |
| DNAJC5 | 2.8105557 | 0.00046298 |
| TOR1B | 1.04682897 | 0.00046345 |
| KLHDC7A | -3.6694776 | 0.00046905 |
| GNG4 | 1.97897903 | 0.00046952 |
| SYBU | -1.5908357 | 0.0004711 |
| CPNE7 | -1.4443463 | 0.00047137 |
| RUVBL1 | 1.03792002 | 0.00047357 |
| ITGA11 | -2.1751338 | 0.00047509 |
| TICAM1 | -1.0970123 | 0.00047718 |
| TMEM14A | 4.36831431 | 0.00047775 |
| APBB2 | -1.5003653 | 0.00047893 |
| EGR3 | -2.785991 | 0.00048174 |
| ACTG1 | -122.71769 | 0.00048806 |
| TIMM50 | 1.1166349 | 0.00048884 |
| DCTPP1 | 3.80150165 | 0.00049011 |
| SELENON | -2.4175529 | 0.00049158 |
| ATP6AP2 | 2.24054511 | 0.00049289 |
| CLIC3 | 3.26436156 | 0.00049463 |
| CUEDC1 | -1.8261928 | 0.00050162 |
| FBN1 | -4.482072 | 0.00050202 |
| CCT2 | 11.2661852 | 0.00050206 |
| RASSF3 | 1.97583167 | 0.00050281 |
| CNN3 | 10.9896565 | 0.0005041 |
| DUSP5 | -5.1859982 | 0.00050441 |
| LRATD2 | 4.73226749 | 0.0005055 |
| KLF4 | -2.2305404 | 0.00050996 |

| | | |
|---|---|---|
| HSPBP1 | -3.0897725 | 0.00051026 |
| KLK7 | 4.23183031 | 0.00051262 |
| TAGLN2 | 52.415164 | 0.00051342 |
| VAMP5 | -4.8460848 | 0.00051615 |
| COMMD3 | -1.0103266 | 0.00051658 |
| PPP1R3C | -3.9268962 | 0.00051772 |
| NUTF2 | 3.33246338 | 0.00051961 |
| F3 | -3.0872051 | 0.00052308 |
| MT2A | -54.719217 | 0.00052449 |
| NTN4 | -7.8234717 | 0.00052449 |
| AKAP11 | 1.01579725 | 0.00052449 |
| RBBP7 | 6.96838355 | 0.00052449 |
| MAT2A | 3.63844181 | 0.00052558 |
| COX6C | -59.18789 | 0.00052585 |
| SRP68 | 1.53774374 | 0.00052741 |
| ARF1 | 20.8635211 | 0.00052952 |
| SHROOM1 | -9.823544 | 0.00053504 |
| F13A1 | -3.5934439 | 0.00053646 |
| SF3B1 | 3.17213271 | 0.00053901 |
| NKAIN1 | -6.0219336 | 0.00054038 |
| DPCD | -1.7145329 | 0.0005463 |
| SOCS3 | -7.4284776 | 0.00054687 |
| RBM42 | -4.3859216 | 0.00054864 |
| ARHGAP40 | -3.0156782 | 0.00054887 |
| TPSAB1 | -7.209905 | 0.00054975 |
| CYBC1 | -1.1971965 | 0.0005509 |
| DNAJB12 | -1.0554549 | 0.00055251 |
| CAPN6 | 5.0685129 | 0.00055847 |
| GAR1 | 1.42862632 | 0.00056688 |

| | | |
|---|---|---|
| GMPR2 | -1.643522 | 0.00056886 |
| ATPAF1 | 1.01471368 | 0.00057262 |
| RPL27A | -13.34646 | 0.00057553 |
| MAP1LC3A | -3.7147704 | 0.00057577 |
| KCNK5 | 3.21592715 | 0.0005776 |
| RTL8C | -7.3511193 | 0.00058557 |
| RAB35 | 1.0305359 | 0.0005887 |
| TMEM248 | 2.13869666 | 0.00059276 |
| FAM83A | 1.08824759 | 0.00059293 |
| SMARCA4 | 1.28810628 | 0.00059369 |
| PLEC | -3.0437316 | 0.00059388 |
| RPP25 | 1.69725204 | 0.00059565 |
| SCP2 | 2.13459102 | 0.00059621 |
| COPE | -4.0154356 | 0.00060249 |
| SMPD1 | -1.7136623 | 0.00061325 |
| RNF144B | 3.14211821 | 0.00061493 |
| CSGALNACT2 | 1.03923329 | 0.00061753 |
| MGAT4B | 2.10642265 | 0.00062085 |
| GLG1 | 1.40786833 | 0.00062469 |
| TMEM87B | 1.7253389 | 0.00062896 |
| TRIM65 | 1.18467466 | 0.00063604 |
| IMPDH1 | 3.3768324 | 0.00063683 |
| TRADD | -1.3305654 | 0.00063844 |
| ZNF706 | 1.84139669 | 0.00064157 |
| MUL1 | -1.1173051 | 0.00064157 |
| SNAPC2 | -3.6127228 | 0.0006429 |
| COL8A1 | -3.0746553 | 0.00064364 |
| OLA1 | 1.79541657 | 0.00064431 |
| ACSL3 | 4.72115826 | 0.00064487 |

| | | |
|---|---|---|
| SPINDOC | 1.2240183 | 0.00064635 |
| SH3D19 | -1.0112216 | 0.00064635 |
| VIRMA | 2.03886232 | 0.00064635 |
| EPHB3 | 7.29412403 | 0.00064851 |
| ID1 | -3.8842917 | 0.00064897 |
| KRT80 | 2.24968811 | 0.00064907 |
| GMFG | -2.5510004 | 0.00064956 |
| ZFP36L1 | -11.850566 | 0.00064996 |
| CDK4 | 3.06164268 | 0.00065036 |
| RBM24 | -2.6968526 | 0.00065334 |
| DLG3 | 1.24661514 | 0.00065894 |
| MACROD1 | -2.2684598 | 0.00066227 |
| MINDY1 | -2.8603651 | 0.00066267 |
| LPIN2 | 1.14320857 | 0.00066384 |
| NOA1 | 1.12288206 | 0.00069986 |
| ANXA7 | 4.27950267 | 0.00070154 |
| ALYREF | 6.33916683 | 0.00070655 |
| COQ10B | 1.30245342 | 0.00071986 |
| B2M | 71.5882233 | 0.00071986 |
| GBP4 | 4.30591782 | 0.00072053 |
| PRRG2 | -1.2063108 | 0.00072232 |
| SPATA20 | -2.7927861 | 0.00072593 |
| HNMT | -1.1755733 | 0.00073719 |
| TARS2 | 1.72886233 | 0.00073901 |
| PRKAG1 | 1.44118326 | 0.00074022 |
| PLEKHF2 | 6.37574563 | 0.00075171 |
| SRSF6 | -2.7131378 | 0.0007543 |
| BABAM1 | -1.1100497 | 0.00075746 |
| RAB4A | 1.67572044 | 0.00076673 |

| | | |
|---|---|---|
| NEK6 | 1.61535542 | 0.00076841 |
| MMP24OS | -1.4965435 | 0.00076971 |
| MAP3K1 | -2.5733342 | 0.00076987 |
| PAXX | -4.5558551 | 0.0007699 |
| LBP | 18.1277339 | 0.00078217 |
| FHL3 | -1.7713007 | 0.000783 |
| HRAS | -3.3417544 | 0.00078369 |
| CTDNEP1 | -1.1453568 | 0.00078473 |
| UQCRQ | -5.8025012 | 0.0007898 |
| ABLIM3 | -1.2199523 | 0.00079441 |
| WFS1 | -2.771331 | 0.00079812 |
| XAGE2 | 2.8451625 | 0.00080257 |
| PSMC4 | 5.21964036 | 0.00080355 |
| UBA1 | 7.23453199 | 0.00080375 |
| UBE2R2 | 1.98445258 | 0.00080498 |
| SEC13 | 2.4787395 | 0.00080629 |
| CDCA4 | 1.00424608 | 0.00080727 |
| PRXL2B | -1.701977 | 0.0008116 |
| PPP2R5A | 3.85104386 | 0.00081742 |
| GNS | 4.87205201 | 0.0008176 |
| USP19 | -1.0147591 | 0.00082596 |
| PTPRF | 8.34102612 | 0.00083507 |
| HNRNPF | 7.92073874 | 0.00083635 |
| DEPP1 | -6.3829665 | 0.00083832 |
| FBP1 | -5.2290605 | 0.00084209 |
| RAP2C | 4.27998197 | 0.00084745 |
| ARHGEF3 | -1.120638 | 0.00086277 |
| UBE2O | 1.01197551 | 0.00086547 |
| KCTD6 | -1.8282052 | 0.00087417 |

| | | |
|---|---|---|
| MRPL52 | -1.6274839 | 0.00088096 |
| EBAG9 | 1.21981884 | 0.00088624 |
| RAB18 | 2.35432988 | 0.00088624 |
| CXCL9 | 18.8230604 | 0.00088743 |
| CCNG2 | -2.976117 | 0.00090503 |
| GAS1 | -2.1119018 | 0.00090515 |
| MORF4L1 | 2.40623631 | 0.00091052 |
| LHFPL2 | 1.15519597 | 0.00091263 |
| CLTB | -5.4713238 | 0.00091595 |
| RTCB | 2.45417171 | 0.00091764 |
| S100A9 | 345.553085 | 0.00091768 |
| STUB1 | -1.6954519 | 0.00091881 |
| NUFIP2 | 2.64647598 | 0.00092432 |
| TMEM106C | 3.77923071 | 0.00092485 |
| CNP | 1.65774232 | 0.0009274 |
| ARRB1 | -1.4814937 | 0.00094987 |
| NPEPPS | 1.16327216 | 0.00096053 |
| TMED7 | 2.96879681 | 0.00096185 |
| GTF3C6 | 6.96699125 | 0.00097011 |
| CDK2AP2 | -4.5018226 | 0.0009747 |
| NDUFC1 | -1.3499643 | 0.00097848 |
| CLCN3 | 1.82321667 | 0.00099686 |
| MSN | 10.0890297 | 0.0009997 |
| CA11 | -1.6776398 | 0.00100575 |
| PHETA2 | -1.0809288 | 0.00101067 |
| MRPL55 | -3.7353382 | 0.00103149 |
| F7 | -1.1769567 | 0.00103333 |
| AKR7A3 | -11.342932 | 0.0010346 |
| MTMR4 | 1.46949321 | 0.00103474 |

| | | |
|---|---|---|
| ITGA3 | -2.4565488 | 0.00103914 |
| TBC1D9 | -19.485792 | 0.00104395 |
| BAX | -2.18268 | 0.00104809 |
| AFF4 | 1.9660456 | 0.00105051 |
| GALNT2 | 1.28892199 | 0.00105335 |
| UBAP1 | 1.66481408 | 0.00106261 |
| CSTF1 | 1.154205 | 0.00106688 |
| CASP14 | 19.6266275 | 0.00107884 |
| PLEKHM2 | -1.2145958 | 0.00108534 |
| AMOTL2 | -1.3878425 | 0.0010923 |
| IL33 | -1.423091 | 0.00110585 |
| HOXB13 | 1.00236794 | 0.00111718 |
| CAPN2 | 5.31615894 | 0.00114397 |
| CSRP1 | -4.4810901 | 0.00114411 |
| LDLRAD3 | -4.4011965 | 0.00114893 |
| RPP25L | -2.2609315 | 0.00114949 |
| RNF19B | 1.75551784 | 0.00114977 |
| POF1B | 1.11151845 | 0.00115345 |
| MARCO | 2.64219043 | 0.00115411 |
| UFM1 | 1.64681978 | 0.00115981 |
| FOLR2 | -2.7547211 | 0.00115981 |
| NUDT8 | 2.45537717 | 0.00116022 |
| ZNF629 | -1.0010733 | 0.00116182 |
| FOXP4 | 3.22940433 | 0.00116493 |
| LAMTOR2 | -6.520277 | 0.00116648 |
| JARID2 | 1.01764465 | 0.00117102 |
| MLF2 | 7.90933648 | 0.00117302 |
| TMEM134 | -1.0260006 | 0.0011769 |
| DKK1 | 2.06577322 | 0.00117931 |

| | | |
|---|---|---|
| RND1 | -2.1565543 | 0.00118687 |
| SEM1 | 1.01231462 | 0.00119208 |
| ATIC | 2.71187595 | 0.001193 |
| RUVBL2 | -3.1895952 | 0.00120223 |
| MRPL49 | 2.7922841 | 0.0012042 |
| PI4KB | 1.93847403 | 0.0012042 |
| PPP4R3A | 1.18779474 | 0.00120555 |
| RPL18A | -17.913398 | 0.00121185 |
| EDNRA | -1.4905683 | 0.00121856 |
| ART3 | 3.19723546 | 0.00122309 |
| TBCB | -2.5247251 | 0.00122329 |
| SDF2 | -1.8291119 | 0.00123699 |
| MYADM | -4.4418444 | 0.00124601 |
| CC2D1A | -1.3050317 | 0.00125907 |
| CTF1 | -1.0479263 | 0.00126557 |
| PRNP | 6.20388974 | 0.00126818 |
| NPR3 | 1.03345964 | 0.00128726 |
| ERBB2 | 42.8856589 | 0.00131402 |
| PPP1R14A | -1.0192622 | 0.00134193 |
| TMED4 | 2.8224561 | 0.00134349 |
| SLPI | 121.314054 | 0.00134563 |
| IFIT3 | 9.14049602 | 0.00134983 |
| TIMM10B | 1.16773853 | 0.00135053 |
| GPR137B | 2.42453549 | 0.00135209 |
| KLF9 | -1.5964103 | 0.00135833 |
| CYP4F22 | -2.7944647 | 0.00137364 |
| ATP8B2 | -1.0379974 | 0.00137962 |
| NOC4L | -1.3731068 | 0.00139293 |
| TXNDC11 | 1.40624055 | 0.00139777 |

| | | |
|---|---|---|
| COIL | 1.28349953 | 0.00140339 |
| CBLC | -2.2989838 | 0.00140597 |
| MSLN | 14.6457561 | 0.00142512 |
| CNDP2 | 1.82087726 | 0.00142676 |
| GYS1 | 1.25707766 | 0.0014278 |
| MOGS | 1.92475599 | 0.00143512 |
| DNASE2 | 3.27038169 | 0.00143568 |
| VWA2 | -1.6774004 | 0.00144159 |
| PUM1 | 1.33206623 | 0.00149152 |
| KCNJ8 | -1.6929963 | 0.00149528 |
| GALNT1 | 3.08907424 | 0.00150719 |
| STAT5B | -1.3442666 | 0.00153583 |
| IRX2 | -5.2852304 | 0.00154713 |
| HEXB | 1.96503296 | 0.00154898 |
| PFKFB2 | 1.1698472 | 0.00155162 |
| HADHA | 3.24710977 | 0.00155304 |
| NDUFB10 | -10.303712 | 0.00156627 |
| TBL1XR1 | 2.38503759 | 0.00156988 |
| TRAF7 | 2.26151216 | 0.0015911 |
| RBM10 | -1.4952673 | 0.0015911 |
| TNFAIP2 | 5.93031413 | 0.0016089 |
| PPP6R3 | 1.93758286 | 0.00161422 |
| PIGX | 1.07097987 | 0.00161817 |
| H2AZ2 | 2.06273279 | 0.00161854 |
| MCM5 | 1.15762556 | 0.00162429 |
| SLC50A1 | 6.13750678 | 0.00164952 |
| GDE1 | 2.22126295 | 0.00165027 |
| SOX10 | 4.89956645 | 0.00165586 |
| MSR1 | 1.11757192 | 0.00165653 |

| | | |
|---|---|---|
| TP53INP2 | 1.91054013 | 0.00166003 |
| CDK12 | 3.69188651 | 0.00166437 |
| BCL3 | -3.0822823 | 0.0016823 |
| MARCHF8 | -1.1789937 | 0.00169375 |
| KCNK6 | -2.0104665 | 0.00169623 |
| SH3KBP1 | 1.2643372 | 0.00170382 |
| AHSA1 | 3.69190959 | 0.00170589 |
| PRRX1 | -2.4320159 | 0.00171126 |
| NEDD9 | -1.1720889 | 0.00171729 |
| NFKBIA | -6.3349939 | 0.00172151 |
| PYGB | 6.3309299 | 0.00172206 |
| CITED4 | -12.672778 | 0.00173113 |
| TFF1 | -274.15677 | 0.00173203 |
| TASOR2 | 2.49666124 | 0.00175271 |
| CHPF | -6.6896591 | 0.00175271 |
| AC024940.1 | 1.03806452 | 0.00176183 |
| S100A11 | 146.884139 | 0.00176183 |
| LOXL2 | -2.5258263 | 0.00176658 |
| BET1L | -1.0861892 | 0.00177464 |
| NCF2 | 1.23654453 | 0.00177581 |
| CNTNAP2 | 1.12086541 | 0.00177614 |
| FUNDC1 | 1.9774744 | 0.00180294 |
| CD164 | 13.1490843 | 0.00180942 |
| SNX1 | -1.376175 | 0.0018169 |
| COL10A1 | -11.363758 | 0.00181782 |
| C11orf80 | 1.23016189 | 0.00182624 |
| PLEK | 2.33385477 | 0.00182785 |
| TPD52L2 | 3.42447637 | 0.00183362 |
| DNAL4 | -1.1658822 | 0.00184782 |

| | | |
|---|---|---|
| PHF20 | 1.01163858 | 0.00185368 |
| VRK2 | 1.99882514 | 0.00185922 |
| AGTRAP | -2.7114252 | 0.0018824 |
| MAP1LC3B | 1.26742145 | 0.00189412 |
| B4GAT1 | -1.9070433 | 0.00189484 |
| LPGAT1 | 2.2408237 | 0.00190899 |
| CHID1 | -1.4758632 | 0.00192808 |
| KLF5 | 4.19201332 | 0.00193383 |
| LRIG1 | -3.2205398 | 0.00194947 |
| IER3 | -9.0690896 | 0.00195818 |
| ERAP1 | 1.0419125 | 0.00196535 |
| PI16 | -2.573036 | 0.00197976 |
| HNRNPA3 | 2.83926381 | 0.00199643 |
| PHGDH | 6.58842516 | 0.00201092 |
| TUSC3 | 2.13484256 | 0.00205107 |
| RUSC1 | 1.52144471 | 0.00205203 |
| BNIPL | -2.5129731 | 0.00205644 |
| RHBDF1 | -1.4055725 | 0.00205644 |
| GNA12 | 1.20023339 | 0.00206814 |
| MANF | 3.62196722 | 0.0020777 |
| PLAT | -12.519476 | 0.00208614 |
| RNF141 | 1.44854361 | 0.0020898 |
| CDS1 | 1.57299767 | 0.00211854 |
| RPS21 | -53.383686 | 0.00213089 |
| TNFSF13B | 1.29812336 | 0.00214287 |
| TNFRSF1A | -2.4149978 | 0.00214536 |
| RPL6 | -13.585428 | 0.00216227 |
| RPS3 | -53.868725 | 0.0021652 |
| USP35 | -1.3637258 | 0.00216696 |

| | | |
|---|---|---|
| TIMM8B | 2.85033987 | 0.00218821 |
| SDSL | -1.3150718 | 0.00220099 |
| ST3GAL4 | 1.46771296 | 0.00221042 |
| DCXR | -9.073488 | 0.00221185 |
| LSM1 | 2.17741166 | 0.0022657 |
| SRSF2 | 2.79872589 | 0.00227056 |
| CYC1 | 9.77523632 | 0.0022751 |
| MTX2 | 1.37943415 | 0.00228169 |
| BCL6 | -1.5762219 | 0.00228743 |
| LPCAT1 | 2.48314387 | 0.00229433 |
| RPL37 | -10.280406 | 0.00230352 |
| FTH1 | -7.9512184 | 0.00233179 |
| PLXND1 | -2.5051142 | 0.00233296 |
| TEX2 | 1.7083395 | 0.00237584 |
| AQP5 | 8.89093654 | 0.00237593 |
| SHISA5 | -3.8213482 | 0.00237593 |
| CPA4 | 1.70116611 | 0.00238268 |
| CPD | 2.98573593 | 0.00238294 |
| FHL2 | -3.0461354 | 0.00239358 |
| VPS72 | 4.04921031 | 0.00240574 |
| LMF2 | -2.935354 | 0.00240782 |
| MMP7 | 33.9607256 | 0.0024381 |
| NRIP3 | -5.2530174 | 0.0024687 |
| HMCES | 1.87610315 | 0.00247856 |
| MED23 | 1.01765576 | 0.00248608 |
| ANP32A | -1.4052439 | 0.00248731 |
| SLC38A1 | 6.9866155 | 0.0024938 |
| FAP | -1.045867 | 0.00251473 |
| TJP3 | -2.2209096 | 0.00254817 |

| | | |
|---|---|---|
| PSMD8 | 5.41798711 | 0.00256006 |
| PSMB4 | 15.3530641 | 0.00256198 |
| COTL1 | 4.38366522 | 0.00256578 |
| CALB2 | 6.85734728 | 0.00257094 |
| NID1 | -3.5832655 | 0.00257094 |
| SIVA1 | -1.287576 | 0.00258454 |
| MCM3AP | 1.07573805 | 0.00259977 |
| ITGB6 | 5.10464824 | 0.00259983 |
| SEC16A | 3.00037977 | 0.00260071 |
| LAMA4 | -1.2241138 | 0.00261299 |
| ARHGAP4 | -1.1684932 | 0.0026166 |
| PFDN4 | 1.79510299 | 0.00264372 |
| COA6 | 1.01884363 | 0.00264524 |
| GPD2 | 2.02840291 | 0.00266112 |
| SOX8 | 1.35461819 | 0.00266714 |
| PLEKHA2 | 1.08177109 | 0.0027237 |
| FABP5 | 2.84088077 | 0.00273626 |
| BYSL | 5.76317982 | 0.00274149 |
| IFI16 | 3.0207086 | 0.00275803 |
| CHI3L1 | 24.5065425 | 0.00275884 |
| EID2 | 1.14793668 | 0.00277177 |
| CD9 | 8.76553402 | 0.00280018 |
| SPOCK1 | -1.6735798 | 0.00282552 |
| CRB3 | -1.146461 | 0.00283064 |
| TRMT1 | -1.2924836 | 0.00285242 |
| NUPR2 | -2.0443532 | 0.00287849 |
| RNF139 | 2.82931284 | 0.00292193 |
| SPTBN2 | 3.40394469 | 0.00292316 |
| SOX4 | 6.64856807 | 0.00292484 |

| | | |
|---|---|---|
| RAB26 | -1.2478567 | 0.00296005 |
| P3H1 | -1.4225932 | 0.0029665 |
| FANCL | 1.13860628 | 0.00296688 |
| MED1 | 4.91042762 | 0.00297836 |
| RFLNB | -1.0016326 | 0.00302129 |
| IFIT2 | 4.14935103 | 0.00302288 |
| BIK | -2.7384501 | 0.00306689 |
| PGAP6 | 1.94383203 | 0.00307093 |
| TUBA1A | -10.126966 | 0.00307933 |
| RPS27 | -69.286462 | 0.00309088 |
| FAM8A1 | 1.52768835 | 0.00309427 |
| TAOK1 | 1.39344956 | 0.00310038 |
| SLC26A2 | 1.62598424 | 0.00310525 |
| GFRA1 | -12.361873 | 0.00310525 |
| EMC10 | -1.041726 | 0.00314135 |
| GSTO1 | -3.5846766 | 0.00314577 |
| PHF23 | -1.1261484 | 0.00315836 |
| FAM234A | -1.962343 | 0.00317737 |
| UBE2S | 1.68669661 | 0.0031891 |
| ARFGAP1 | -2.1138884 | 0.0032026 |
| LRRN2 | -1.3396219 | 0.0032251 |
| PRRX2 | -1.5679539 | 0.00325224 |
| CRABP1 | 14.3152177 | 0.0032679 |
| MAPK15 | -1.6663717 | 0.00326906 |
| CXCL1 | 1.68685378 | 0.00327016 |
| NR4A2 | -2.2032196 | 0.00329102 |
| MUC1 | -24.368747 | 0.00330549 |
| ANKRD40 | 1.5732971 | 0.00335129 |
| ADGRL1 | -1.0852656 | 0.00337238 |

| | | |
|---|---|---|
| FOSL2 | -2.792817 | 0.00337322 |
| SYK | 1.06084871 | 0.00340052 |
| NRSN2 | -2.1337105 | 0.00340607 |
| PAF1 | -3.2085939 | 0.00340923 |
| KIRREL1 | -1.0913204 | 0.00341666 |
| LAMTOR1 | -3.1951839 | 0.00343325 |
| TUBG1 | 2.04430801 | 0.00343511 |
| MAPK13 | 1.48879012 | 0.00346836 |
| KRT23 | 7.71628271 | 0.0034761 |
| BASP1 | -3.7427596 | 0.00348292 |
| ADGRB2 | -1.3956639 | 0.00348746 |
| ANAPC11 | -2.6540928 | 0.00348931 |
| SLC49A3 | -1.7616579 | 0.00350419 |
| CIB1 | -12.04857 | 0.00350419 |
| BHLHE41 | -2.7073425 | 0.00354379 |
| SECTM1 | 3.33737737 | 0.00359283 |
| NTHL1 | -1.5912255 | 0.00359923 |
| ARFIP2 | -2.1836695 | 0.00360231 |
| COLEC12 | -1.5954662 | 0.00360642 |
| PREX1 | -7.1431235 | 0.00362919 |
| TC2N | 3.8012093 | 0.0037277 |
| LAPTM4B | 25.0436476 | 0.00378166 |
| RHOG | -2.7200928 | 0.0037955 |
| GOLT1A | -1.3423798 | 0.00381995 |
| SLC2A4RG | -5.0741006 | 0.00383949 |
| COX17 | -2.0258028 | 0.00386775 |
| ADIPOR1 | 5.62167656 | 0.00390774 |
| TACC3 | 1.02612828 | 0.00390821 |
| PLEKHJ1 | -1.0430433 | 0.00392096 |

| | | |
|---|---|---|
| DVL3 | 1.42968125 | 0.0039288 |
| ST8SIA6 | -1.3094283 | 0.0039288 |
| H19 | -4.3501064 | 0.0039522 |
| GFRA3 | 1.11788892 | 0.00398551 |
| XAB2 | -2.7017686 | 0.00398646 |
| GSTM3 | -6.793245 | 0.00402257 |
| EFNA5 | 1.40776041 | 0.00402601 |
| TPD52 | 5.50741851 | 0.00403539 |
| ANXA3 | 1.47128624 | 0.00404471 |
| CP | 7.49099858 | 0.00407396 |
| CXCL8 | 1.60835159 | 0.00407396 |
| CRISP3 | 56.1741908 | 0.00408707 |
| MRPS34 | -7.7682956 | 0.00409983 |
| GINS2 | 1.23036494 | 0.00411773 |
| KEAP1 | -1.8655173 | 0.00415235 |
| MED13 | 1.50771029 | 0.00416017 |
| TRMT12 | 1.48350702 | 0.0041669 |
| AREG | -19.365371 | 0.00416835 |
| LEP | -1.1524459 | 0.00423536 |
| NCOA2 | 1.30649451 | 0.00423899 |
| LANCL1 | 1.55244983 | 0.00425716 |
| MICU2 | 1.24536276 | 0.00428229 |
| NAPSB | -1.5063559 | 0.00430983 |
| CIAO1 | 1.00206037 | 0.00432638 |
| PDAP1 | 3.51180186 | 0.0043382 |
| CPA3 | -3.7328576 | 0.00435856 |
| ODAM | 1.50522415 | 0.00435909 |
| MUC5B | 2.5932685 | 0.00441685 |
| HPF1 | 1.13965175 | 0.00444412 |

| | | |
|---|---|---|
| LSR | 7.4783248 | 0.00445628 |
| GPNMB | 7.76834467 | 0.00445877 |
| SCG5 | -1.2657247 | 0.00448176 |
| ZKSCAN1 | 2.24766037 | 0.00448613 |
| RHPN2 | 1.64156088 | 0.00450187 |
| MEDAG | -1.0090123 | 0.00451047 |
| POP7 | 3.77963732 | 0.00455521 |
| CA12 | -14.040301 | 0.00455555 |
| SURF2 | -1.6981093 | 0.00458229 |
| TCIRG1 | -2.6982896 | 0.00460226 |
| C1R | -6.4271918 | 0.00467098 |
| SEC61G | 3.79811977 | 0.00469367 |
| KLHL12 | 1.95335117 | 0.00470228 |
| MYB | -2.8739977 | 0.00471957 |
| SH2D4A | 1.08819987 | 0.0047236 |
| KDELR3 | 3.86180549 | 0.00475464 |
| TMEM50B | 1.08384455 | 0.00476411 |
| TMEM254 | 2.17223445 | 0.00476872 |
| MRPL16 | 1.25087286 | 0.00479573 |
| SERPINF2 | -1.6682319 | 0.00479923 |
| MNDA | 1.01527287 | 0.00479923 |
| SURF1 | -2.3291016 | 0.0048195 |
| MXRA5 | -6.5983796 | 0.00482603 |
| IFITM3 | -65.23641 | 0.00494586 |
| RAE1 | 1.30511435 | 0.00495447 |
| ENPP4 | 1.63715761 | 0.00497019 |
| CFAP36 | -1.2035429 | 0.00503791 |
| TRAFD1 | 1.38761799 | 0.00506847 |
| FCMR | -1.3250232 | 0.00509937 |

| | | |
|---|---|---|
| AP3S1 | 2.00835472 | 0.00515038 |
| CD82 | 2.67335433 | 0.00519538 |
| RPL8 | -111.0675 | 0.00521198 |
| ZNRD2 | -1.2033157 | 0.00523278 |
| CXCL14 | -122.21572 | 0.0052374 |
| BBOX1 | 1.33758277 | 0.00523876 |
| PEF1 | -2.2666685 | 0.00524917 |
| HNRNPA1 | -5.7778503 | 0.00526449 |
| CST1 | -5.9825614 | 0.00527306 |
| EIF4A3 | 1.88567719 | 0.00529643 |
| CCL13 | 1.08290125 | 0.00533517 |
| CDKN1B | -4.8014329 | 0.00542011 |
| ZRANB2 | 1.11245886 | 0.00542094 |
| HNRNPDL | -2.2416719 | 0.00548228 |
| ACTN1 | -3.9102345 | 0.00548255 |
| MAST4 | -1.0320592 | 0.00552592 |
| ITPA | -2.5001558 | 0.00556578 |
| KAT2A | -1.8156321 | 0.00557583 |
| CTTN | -9.8811997 | 0.00560746 |
| TRAPPC1 | -3.2874949 | 0.00561591 |
| ERBB3 | -5.2449306 | 0.00561669 |
| PRR11 | 3.35004936 | 0.00566026 |
| PLPP3 | -1.0015552 | 0.00566905 |
| HSPE1 | 1.79448729 | 0.00575224 |
| SEC11C | 1.79828876 | 0.00577421 |
| MRPL36 | 1.65792167 | 0.00579544 |
| ABHD4 | -1.0356447 | 0.00581981 |
| VCL | 2.25975096 | 0.00582371 |
| BCAS2 | 6.42382021 | 0.00586053 |

| | | |
|---|---|---|
| MRPL57 | -1.7675905 | 0.00592499 |
| CTXN1 | -4.4440262 | 0.00595051 |
| PLOD2 | 2.69768224 | 0.00601945 |
| SMO | 1.05082647 | 0.00603516 |
| TMEM98 | -1.3159215 | 0.00617396 |
| FILIP1L | -1.1126066 | 0.00622921 |
| SEPTIN2 | 3.2962241 | 0.00624507 |
| MCAM | -2.0550943 | 0.0063098 |
| RUNX3 | 1.02135215 | 0.00631167 |
| DCTD | -1.3569403 | 0.00636252 |
| VDAC3 | 5.84547876 | 0.0063799 |
| S100A7A | 2.8536888 | 0.00646841 |
| HIPK2 | 1.15421839 | 0.00649415 |
| MBOAT7 | -3.4111606 | 0.00651079 |
| TARBP1 | 1.17641686 | 0.00652248 |
| TPSG1 | -1.7761006 | 0.00652958 |
| NOL3 | -1.2970835 | 0.00658356 |
| ARPC2 | 2.29700155 | 0.00662356 |
| BCAT2 | -1.3490514 | 0.00665419 |
| ANTXR1 | -5.4902559 | 0.00666154 |
| SPINT1 | 6.20256185 | 0.00673002 |
| CHRDL1 | -1.4070391 | 0.00675556 |
| PRELID1 | 1.04396045 | 0.00676736 |
| CLDN7 | 4.15436116 | 0.00681229 |
| MYBBP1A | 1.05656102 | 0.00687444 |
| VANGL2 | 1.95494692 | 0.0069386 |
| GALNT7 | 2.9451631 | 0.00698476 |
| SH3PXD2A | -1.2519207 | 0.00699125 |
| CMTM6 | 3.14351602 | 0.00705267 |

| | | |
|---|---|---|
| ZFAND5 | 1.57387574 | 0.00719295 |
| SNN | 1.44833173 | 0.00721683 |
| CORO2A | 1.41412799 | 0.00723448 |
| DPM3 | -5.9627882 | 0.00732675 |
| TYROBP | -9.1342736 | 0.00736065 |
| AKAP1 | 2.27069641 | 0.00736556 |
| MRPS7 | 1.8774649 | 0.00740859 |
| CYB561D2 | -1.1792351 | 0.0074739 |
| LAMA3 | -1.1500632 | 0.0074865 |
| COX7A2L | 1.0112894 | 0.00756405 |
| WBP2 | -3.066599 | 0.0075821 |
| PHF10 | 1.08373175 | 0.00763672 |
| CDK9 | -1.7832021 | 0.00766726 |
| TMEM79 | 1.49983244 | 0.00766814 |
| SRSF7 | 1.58013644 | 0.00770606 |
| SKAP1 | -1.401561 | 0.00774554 |
| PLEKHS1 | 1.73118361 | 0.00781581 |
| CBX6 | -1.2670479 | 0.00782434 |
| FPGS | -1.349458 | 0.00786038 |
| YWHAH | 5.11301509 | 0.00793389 |
| GUCY1A1 | 1.02172375 | 0.00794433 |
| PLEKHO1 | -1.1650997 | 0.00794869 |
| SAT1 | 16.5945702 | 0.00802158 |
| TERF2IP | -1.1101831 | 0.00802535 |
| RAMP1 | -6.9110333 | 0.00804693 |
| CTNNB1 | 4.81699188 | 0.008074 |
| UBA7 | -1.2350372 | 0.00807473 |
| DDX17 | -4.9404998 | 0.00807523 |
| ATP5PF | 2.27072651 | 0.0081163 |

| | | |
|---|---|---|
| RPL7P9 | -2.3718441 | 0.0081163 |
| DDAH1 | 1.79878632 | 0.00828906 |
| TTC9 | 1.02967726 | 0.00831648 |
| PALLD | -2.8948807 | 0.00833601 |
| TTC3 | 2.08426836 | 0.00843359 |
| B3GNT7 | 1.42890386 | 0.00844136 |
| S100A8 | 106.997404 | 0.00846853 |
| NET1 | 5.22112521 | 0.00865352 |
| SLC52A2 | 2.66968674 | 0.00866887 |
| CLDN1 | 5.36385929 | 0.00868348 |
| CA9 | 2.51981204 | 0.00873679 |
| MRPS15 | 2.34569953 | 0.0087537 |
| PPARD | 1.1359573 | 0.00882169 |
| MRPL40 | -1.4958337 | 0.00882245 |
| MAP2K3 | 1.28909129 | 0.00882278 |
| CCL21 | -7.4343422 | 0.00884189 |
| ZMYND19 | 1.0075777 | 0.00907964 |
| ANO6 | 1.11187054 | 0.00909243 |
| IL4R | -1.0953094 | 0.00910636 |
| ERBB4 | -1.0834008 | 0.00921911 |
| ROMO1 | -13.614899 | 0.00922842 |
| CST9 | -7.2542081 | 0.00925931 |
| IST1 | 1.06086722 | 0.0092696 |
| FOLR1 | 4.38156575 | 0.0093029 |
| TRIB1 | -5.388101 | 0.00933102 |
| PCDH1 | -1.8948882 | 0.009372 |
| ELN | -6.7869523 | 0.00945344 |
| PDLIM1 | -10.4717 | 0.00947399 |
| MELTF | 2.1310147 | 0.00952845 |

| | | |
|---|---|---|
| CD276 | -2.0094935 | 0.00954556 |
| PPP6C | 1.92536333 | 0.00959161 |
| IFIT1 | 7.55431432 | 0.00959161 |
| RPS6KB1 | 1.84790096 | 0.00964627 |
| KCNF1 | -1.9494958 | 0.00969628 |
| ZYX | -4.9499149 | 0.00976277 |
| CADPS2 | 1.21951795 | 0.00977375 |
| NEURL1 | -1.2047717 | 0.00978258 |
| MBNL1 | 1.19703248 | 0.00983099 |
| MS4A7 | -6.6807535 | 0.00989665 |
| VGLL1 | 6.49394125 | 0.00992327 |
| COASY | -1.8870478 | 0.00998355 |
| MAB21L4 | 3.69798219 | 0.00998648 |

Abbreviation: DEGs = differentially expressed genes.
