# Supplementary material for: Cuproptosis-associated genes and immune microenvironment characterization in breast cancer
Source: Medicine (Baltimore). 2022 Dec 16;101(50):e32301. doi: 10.1097/MD.0000000000032301 (PMC9771175; doi:10.1097/MD.0000000000032301)
Supplement: Supplementary file 2 [file medi-101-e32301-s002.pdf]

Table 2. Prognostic DEGs were identified by the univariate Cox regression analysis.

ID	HR	HR.95L	HR.95H	pvalue
NIPA2	1.11389332	1.04910479	1.18268293	0.00041892
NCBP1	1.05716446	1.02678101	1.08844699	0.00018679
PEX13	1.09724659	1.04107783	1.15644579	0.00053719
TOMM70	1.03111674	1.0135588	1.04897885	0.00047073
RAD23B	1.01832448	1.00970524	1.0270173	2.83E-05
MTHFD2	1.0584824	1.02716762	1.09075185	0.00020776
PSMD7	1.07817762	1.03587215	1.12221087	0.00022812
PSMD14	1.08104439	1.03682117	1.12715384	0.00025544
VTA1	1.09651287	1.05322644	1.14157832	7.34E-06
RAB1A	1.01824698	1.00782821	1.02877346	0.00056901
UBE2A	1.07474728	1.03561573	1.11535745	0.00013935
TARS1	1.02339809	1.0102175	1.03675064	0.00047054
NOP53	0.98454262	0.97589232	0.99326959	0.00054057
MORC4	1.08932445	1.04462293	1.13593883	6.28E-05
COPB2	1.06274318	1.02821067	1.09843547	0.00030548
SEC24A	1.10640875	1.05042033	1.16538142	0.00013532
ESRP1	1.01448394	1.00653418	1.02249649	0.00034026
MRPL39	1.03853862	1.01802753	1.05946297	0.00020281
TCP1	1.01720066	1.00953185	1.02492772	1.00E-05
C5orf22	1.08963926	1.03581207	1.14626365	0.00089623
MPHOSPH10	1.07071211	1.03065594	1.11232507	0.00044452
POLR2B	1.02681723	1.01489747	1.03887698	8.91E-06
TXNDC9	1.10681398	1.05341807	1.16291644	5.75E-05
VPS35	1.02145483	1.00957351	1.03347597	0.00037642
SRP72	1.0234573	1.01323054	1.03378729	6.03E-06
KIF21A	1.11972352	1.05772398	1.18535722	9.99E-05
PPAT	1.08280143	1.03971919	1.12766884	0.0001229

HSPA9	1.02331343	1.0134792	1.03324309	2.90E-06
NDUFS1	1.07992477	1.04388474	1.11720907	9.00E-06
PIK3CB	1.09447428	1.04008118	1.15171197	0.00051858
GNAI2	0.96554114	0.94996754	0.98137005	2.37E-05
PGK1	1.00860061	1.00579357	1.01141548	1.72E-09
SLC35A2	1.03327799	1.01638314	1.05045368	9.94E-05
CS	1.0347178	1.01807283	1.05163491	3.71E-05
LSG1	1.06442025	1.03028899	1.0996822	0.00017372
ATP5F1B	1.00373872	1.0020597	1.00542056	1.25E-05
PRDX1	1.00436222	1.00241524	1.00631297	1.10E-05
RAD1	1.15368472	1.06193916	1.25335658	0.00072114
RC3H2	1.08992336	1.03622555	1.14640383	0.00083647
RPS9	0.99445259	0.99124606	0.9976695	0.00073568
KIF5B	1.01531796	1.00653983	1.02417265	0.00060068
RILPL2	0.82012737	0.73618295	0.91364369	0.00031912
TGFB1	0.94869528	0.92118482	0.97702733	0.00045167
MMADHC	1.03745749	1.01993009	1.0552861	2.34E-05
ATG4A	1.18414021	1.09474265	1.28083806	2.44E-05
EIF3J	1.03279468	1.01508097	1.05081749	0.00025641
LARP4	1.0707781	1.03236075	1.11062507	0.00024409
ORC3	1.06415687	1.02754919	1.10206875	0.00049852
TNFRSF14	0.86010102	0.80073514	0.92386824	3.63E-05
PAICS	1.02108601	1.01137364	1.03089165	1.88E-05
RPL29	0.99700709	0.99530907	0.99870801	0.0005679
STRAP	1.00496709	1.00235009	1.00759093	0.00019578
AARS1	1.03410982	1.01373828	1.05489073	0.00095281
PGM3	1.07800104	1.03220287	1.12583125	0.00069664
PSMG1	1.03252291	1.01624108	1.04906559	7.93E-05
POP1	1.10108826	1.04124848	1.16436699	0.00073087

PAFAH1B1	1.08337634	1.0350822	1.13392376	0.00057752
HBS1L	1.05826476	1.03400707	1.08309153	1.70E-06
MRPL13	1.04034751	1.02001481	1.06108552	8.57E-05
PCMT1	1.0357372	1.02105242	1.05063318	1.44E-06
MTDH	1.01665746	1.00749985	1.0258983	0.00034565
MPZL3	1.09064268	1.04150208	1.14210185	0.00022541
PTGES3	1.01023052	1.00622893	1.01424803	5.00E-07
UBXN4	1.03074844	1.01325326	1.04854569	0.00052557
EIF2AK3	1.12267316	1.05060622	1.19968358	0.00062998
ZBTB11	1.17862909	1.08951395	1.27503327	4.18E-05
TMEM167A	1.03689906	1.02118081	1.05285924	3.33E-06
ETFA	1.03941895	1.02227762	1.0568477	5.19E-06
GPR157	1.12450396	1.06596926	1.18625294	1.69E-05
CMTM4	1.04904435	1.02022971	1.07867281	0.00075347
ARMC1	1.02905823	1.01172707	1.04668626	0.00094872
RPLP1	0.99796988	0.99679268	0.99914847	0.00073926
LACTB2	1.0886989	1.04204829	1.13743796	0.0001428
PCYT1A	1.06799599	1.03221354	1.10501887	0.00015466
YWHAZ	1.00309429	1.00130649	1.00488527	0.00068757
BCLAF1	1.05931204	1.02672926	1.09292883	0.00030054
DNAJC13	1.11217914	1.05134567	1.17653259	0.00021171
OSGIN2	1.03446112	1.01703845	1.05218225	9.25E-05
HLTF	1.05599366	1.02282918	1.09023348	0.00081863
APOOL	1.16811572	1.08984967	1.25200234	1.13E-05
IP6K2	0.90928132	0.86563557	0.9551277	0.00015112
TMEM70	1.05605363	1.02498069	1.08806855	0.00034461
NUP43	1.08371514	1.04461374	1.12428017	1.80E-05
NSF	1.04011457	1.02126595	1.05931106	2.50E-05
ATP11B	1.05410006	1.02458869	1.08446145	0.00027626

SNRNP200	1.02336923	1.00988408	1.03703446	0.00064197
RPL9	0.99060646	0.98519782	0.9960448	0.00072829
HSPH1	1.01921727	1.00985663	1.02866467	5.27E-05
XRN2	1.01446087	1.00680773	1.0221722	0.00020242
TATDN1	1.09647976	1.04548743	1.14995919	0.00015019
PDLIM4	0.90537988	0.85447901	0.95931289	0.00076
PRRC1	1.08442729	1.04601885	1.12424605	1.06E-05
STIP1	1.01228085	1.00512803	1.01948458	0.00074162
CHMP3	1.08210715	1.03512584	1.13122081	0.00049331
YWHAB	1.0149761	1.00839546	1.02159969	7.50E-06
CAND1	1.01793521	1.00781934	1.02815261	0.00048574
MOB4	1.0755207	1.03032084	1.12270347	0.00088883
CLOCK	1.13491872	1.05827577	1.21711234	0.00038862
PERP	1.00566244	1.00266888	1.00866494	0.00020539
RPL31	0.99343471	0.98955137	0.99733329	0.00097997
UTP23	1.07258555	1.04205551	1.10401006	1.97E-06
DOP1B	1.03390298	1.01382845	1.054375	0.00085976
EMC2	1.07486365	1.03550398	1.11571937	0.00014888
POLR2K	1.01487783	1.00717787	1.02263665	0.00014436
TPT1	0.99532175	0.99259581	0.99805517	0.00080459
SCARB2	1.03842969	1.01649665	1.06083598	0.00053579
HSP90AA1	1.0011	1.00053594	1.00166437	0.00013172
CCNT1	1.16389071	1.08368675	1.25003059	3.10E-05
DIP2B	1.14032528	1.08912608	1.19393133	2.11E-08
POLR2C	1.032148	1.0187585	1.04571347	2.04E-06
MRPL30	1.13490483	1.05571064	1.22003977	0.00060595
GPR107	1.04334118	1.01912159	1.06813634	0.00039927
CBX5	1.04664535	1.02072714	1.07322168	0.00036591
C8orf33	1.0245595	1.01163676	1.03764732	0.00017937

ADK	1.0623945	1.02519981	1.10093863	0.00087253
BPNT2	1.01995555	1.00935838	1.03066398	0.0002089
TMEM184C	1.1033138	1.04405769	1.16593301	0.00048171
BNIP3	1.03119764	1.01339884	1.04930905	0.00054367
RAB2A	1.01555655	1.00762559	1.02354992	0.00011382
RAB22A	1.06596926	1.02698007	1.10643866	0.00077858
GRPEL1	1.09926554	1.04704002	1.15409603	0.00013847
GSDMC	1.04632423	1.02146754	1.07178578	0.00022297
WASHC5	1.01823645	1.00775311	1.02882885	0.00062015
ZNF623	1.06839908	1.03370733	1.1042551	8.55E-05
VAPB	1.05803634	1.0295855	1.08727337	4.98E-05
RTN3	1.00839821	1.00356488	1.01325483	0.0006458
GRHL2	1.05043318	1.02258831	1.07903626	0.00033126
YIPF5	1.06767295	1.0313754	1.10524794	0.00020681
VDAC2	1.02090145	1.01001567	1.03190455	0.00015556
RABGAP1	1.07414641	1.03084228	1.11926969	0.00065736
TNIP1	0.94368087	0.91733701	0.97078126	6.00E-05
MAL2	1.00240013	1.00115763	1.00364416	0.00015171
ARL8B	1.0193483	1.007877	1.03095016	0.00090413
MDC1	1.04205039	1.02446411	1.05993856	2.10E-06
TMEM245	1.08794464	1.03827712	1.13998808	0.00040698
GYPC	0.88721287	0.83046363	0.94784004	0.00038764
GOT2	1.01774711	1.00747847	1.02812041	0.0006739
MSC	0.81395371	0.72370992	0.91545055	0.00059618
DLG3	1.07180718	1.03179517	1.11337082	0.00035371
NUFIP2	1.02320571	1.01190756	1.03463001	5.13E-05
POF1B	1.05047159	1.02172637	1.08002553	0.00050461
GPR137B	1.02926232	1.01174047	1.04708762	0.00099365
BCL3	0.96336656	0.94507974	0.98200722	0.00013518

NFKBIA	0.97611854	0.96559241	0.98675943	1.25E-05
TASOR2	1.0248831	1.01049657	1.03947444	0.00065522
PHF20	1.05103959	1.0210559	1.08190376	0.00074882
RNF139	1.01712476	1.00696904	1.02738291	0.00091186
TAOK1	1.0387024	1.02032232	1.05741357	3.07E-05
NCOA2	1.04461146	1.01807808	1.07183636	0.00088471
SPINT1	1.01017352	1.0043084	1.0160729	0.00065676

Abbreviations: DEGs = differentially expressed genes.
