# Supplementary material for: Cuproptosis-associated genes and immune microenvironment characterization in breast cancer
Source: Medicine (Baltimore). 2022 Dec 16;101(50):e32301. doi: 10.1097/MD.0000000000032301 (PMC9771175; doi:10.1097/MD.0000000000032301)
Supplement: Supplementary file 3 [file medi-101-e32301-s003.pdf]

Table 3. Hub DEGs were identified by the LASSO analysis

ID	Coef
COPB2	0.03580477
MRPL39	0.03492509
PGK1	-0.0044054
PRDX1	0.00464861
PCMT1	0.02492791
MPZL3	0.08150989
LACTB2	0.07802452
HSPH1	0.0145334
DIP2B	0.09696833
DLG3	0.07129033
NFKBIA	-0.0301151

Abbreviations: DEGs = differentially expressed genes, LASSO = least absolute shrinkage and selection operator.
