# Supplementary material for: Cuproptosis-associated genes and immune microenvironment characterization in breast cancer
Source: Medicine (Baltimore). 2022 Dec 16;101(50):e32301. doi: 10.1097/MD.0000000000032301 (PMC9771175; doi:10.1097/MD.0000000000032301)

Supplementary figure 1. The correlation of hub CAGs and PR (A), ER (B), HER2 statue (C), and breast cancer stage (D).

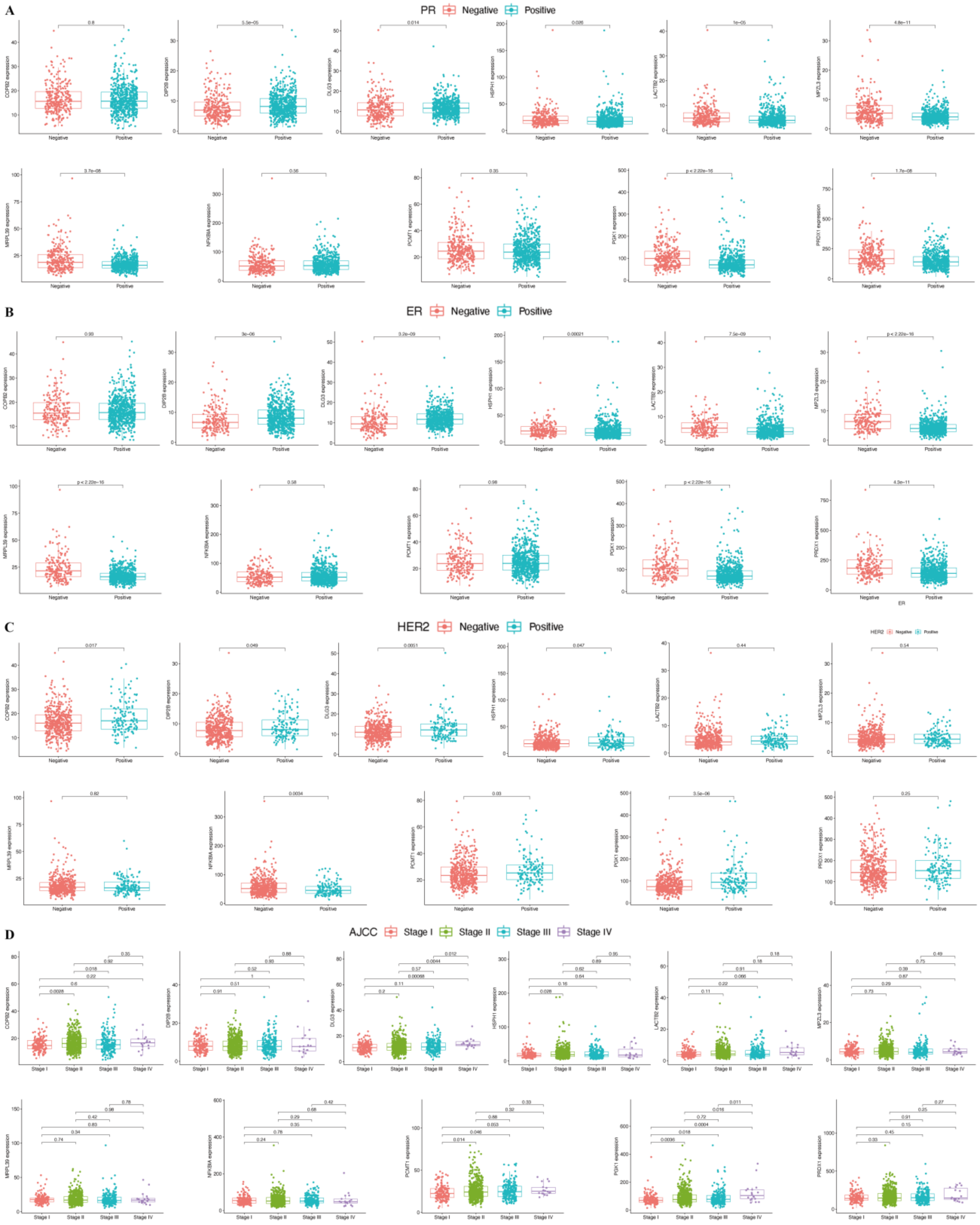

Supplement: Supplementary file 4 [file medi-101-e32301-s004.pdf]
